# Autophagic flux-lipid droplet biogenesis cascade sustains mitochondrial fitness in colorectal cancer cells adapted to acidosis

**DOI:** 10.1038/s41420-025-02301-6

**Published:** 2025-01-25

**Authors:** Xiaojie Liu, Xue Sun, Wenqing Mu, Yanan Li, Wenqing Bu, Tingting Yang, Jia Zhang, Rui Liu, Jiayu Ren, Jin Zhou, Peishan Li, Yufang Shi, Changshun Shao

**Affiliations:** 1 The Third Affiliated of Soochow University, State Key Laboratory of Radiation Medicine and Protection, Institutes for Translational Medicine, Soochow University Medical College, Suzhou, Jiangsu China; 2 Biochip Laboratory, Yantai Yuhuangding Hospital Affiliated to Medical College of Qingdao University, Yantai, China; 3 Department of General Surgery, The First Affiliated Hospital of Soochow University, Suzhou, Jiangsu China

**Keywords:** Cancer metabolism, Cancer metabolism, Oncogenesis

## Abstract

Cancer development is associated with adaptation to various stressful conditions, such as extracellular acidosis. The adverse tumor microenvironment also selects for increased malignancy. Mitochondria are integral in stress sensing to allow for tumor cells to adapt to stressful conditions. Here, we show that colorectal cancer cells adapted to acidic microenvironment (CRC-AA) are more reliant on oxidative phosphorylation than their parental cells, and the acetyl-CoA in CRC-AA cells are generated from fatty acids and glutamine, but not from glucose. Consistently, CRC-AA cells exhibit increased mitochondrial mass and fitness that depends on an upregulated autophagic flux-lipid droplet axis. Lipid droplets (LDs) function as a buffering system to store the fatty acids derived from autophagy and to protect mitochondria from lipotoxicity in CRC-AA cells. Blockade of LD biogenesis causes mitochondrial dysfunction that can be rescued by inhibiting carnitine palmitoyltransferase 1 α (CPT1α). High level of mitochondrial superoxide is essential for the AMPK activation, resistance to apoptosis, high autophagic flux and mitochondrial function in CRC-AA cells. Thus, our results demonstrate that the cascade of autophagic flux and LD formation plays an essential role in sustaining mitochondrial fitness to promote cancer cell survival under chronic acidosis. Our findings provide insight into the pro-survival metabolic plasticity in cancer cells under microenvironmental or therapeutic stress and imply that this pro-survival cascade may potentially be targeted in cancer therapy.

## Introduction

Cancer cells are metabolically plastic depending on the levels of nutrients, oxygen, metabolites and growth factors in their microenvironment [1]. The enhanced utilization of glucose and the excessive production of lactate creates an acidic microenvironment even in the presence of oxygen, which is also known as extracellular acidosis [2–4]. Emerging evidence suggests that extracellular acidosis may select cancer cells with unique metabolic phenotypes, such as the conversion from glycolysis to OXPHOS [5, 6]. Glutamine metabolism is preferred to glucose metabolism in cancer cells exposed to acidosis to limit H^+^ generation from glycolysis [7, 8]. Fatty acids β-oxidation (FAO), a main source of acetyl-CoA, supports TCA cycle in response to ambient acidosis [9–11].

As hubs for metabolic reactions, mitochondria produce TCA cycle intermediates that serve as substrates for de novo synthesis of lipids and nonessential amino acids as well as NADH and FADH2 that sustains electron transport chain to generate ATP. Although cancer cells engage “Warburg” metabolism under favorable propagation conditions, they rely on mitochondria in some cases when experiencing various types of stress [12–14]. Lactic acidosis with glucose deprivation induces mitochondrial biogenesis to support survival and proliferation in lung adenocarcinoma cells [15]. However, the role of mitochondria in tumor cells under chronic acidosis remains to be determined.

Autophagy is an evolutionarily conserved, catabolic process in which cytosolic components and organelles sequestered in double membrane-bound vesicles are delivered to lysosomes for degradation and recycling [16]. Autophagy is considered an adaptive response for survival during stress, and as a result can compromise the efficacy of anti-cancer treatments [17]. A growing body of evidence suggests that autophagy can lead to metabolic rewiring [18, 19]. During periods of high autophagy, lipid droplets (LDs) emerge in great amounts to protect mitochondria from various cytotoxic lipid species [20, 21]. Autophagy-related protein ATG9A depletion blocks transfer of fatty acids from lipid droplets to mitochondria and utilization of fatty acids in mitochondrial respiration [22].

In this study, we demonstrated a crucial role of mitochondrial homeostasis in sustaining cancer cell survival during chronic acidosis. Colorectal cancer cells adapted to acidic microenvironment become more relied on mitochondria, which are increased in mass and produce more ATP. FA derived from autophagy is channeled into LDs to prevent lipotoxicity to mitochondria. While mitochondrial function is disrupted when the formation of LDs is inhibited, it can be rescued by the blockade of FA transport to mitochondria. Thus, the cascade of autophagy, LD formation and mitochondrial fitness that sustains cancer cell survival under adverse conditions can be targeted for cancer management associated with malignancy progression and chemoresistance.

## Results

### Increased mitochondrial mass and fitness in colorectal cancer cells under acidic microenvironment

To evaluate possible metabolism remodeling in tumor cells under acidic environment, we performed RNA sequencing (RNA-seq) to identify the pathways that are enriched in acidic-adapted colorectal cancer cells (Supplementary Table 1). The data revealed that CRC-AA cells exhibit several distinct features when compared to controls (Supplementary Fig. 1A). Genes participating in oxidative phosphorylation (OXPHOS) were highly enriched in HCT15-AA cells. On the contrary, glycolysis was more active in HCT15 than in HCT15-AA cells. Moreover, fatty acid metabolism and cholesterol homeostasis were also upregulated in HCT15-AA cells.

**Fig. 1:**
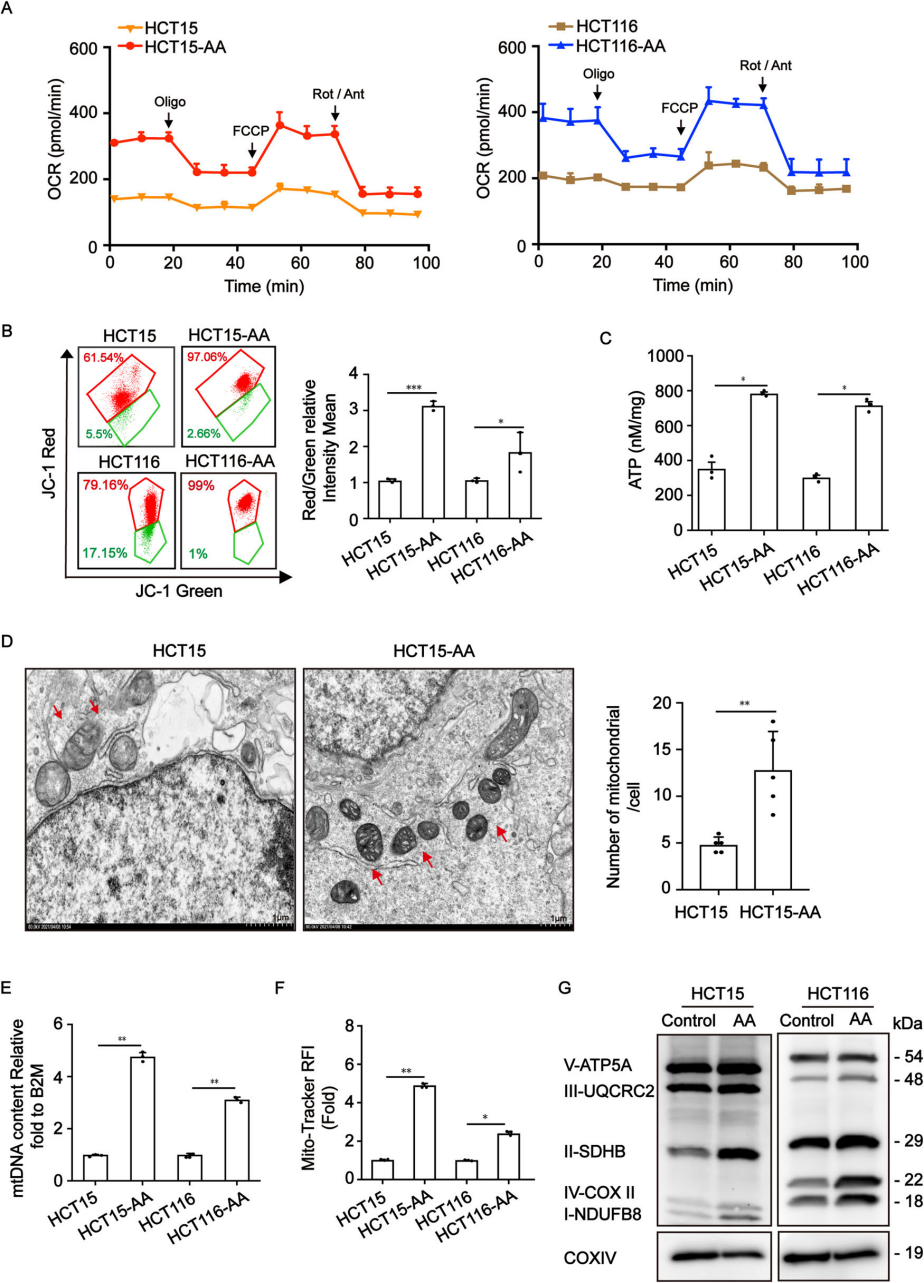
Increased mitochondrial mass and fitness in colorectal cancer cells under acidic microenvironment. **A** Measurements of Oxygen consumption rate (OCR) in CRC and CRC-AA cells. **B** Measurements of MMP (by JC-1) in CRC and CRC-AA cells. **C** Steady-state cellular ATP levels were measured using an ATP luciferase Kit in CRC and CRC-AA cells. **D** Mitochondria in CRC and CRC-AA cells examined with TEM (arrows: mitochondria). **E** mtDNA copy number quantified in CRC and CRC-AA cells. **F** Mitochondrial content was quantified by flow cytometry in CRC and CRC-AA cells. **G** Western blot analysis of OXPHOS protein level in CRC and CRC-AA cells. COX IV was used as a loading control. *p < 0.05, **p < 0.01, ***p < 0.001, ns indicates *p* > 0.05.

To verify that OXPHOS is upregulated in CRC-AA cells, we measured oxygen consumption rate (OCR). The maximal OCR was significantly increased in HCT15-AA cells and HCT116-AA cells (Fig. 1A). JC-1 aggregates in normal mitochondria and emits red fluorescence, but emits green fluorescence when mitochondrial membrane potential (MMP) was depolarized. The ratios of red to green fluorescence were much higher in CRC-AA than in CRC parental cells (Fig. 1B). Consistent with the high OCR and MMP in CRC-AA cells, the intracellular ATP levels were also increased (Fig. 1C), suggesting that colorectal cancer cells under acidic microenvironment possess more robust mitochondrial function.

We next determined whether the increased OXPHOS in CRC-AA cells was associated with high number of mitochondria. As shown in Fig. 1D, the numbers of mitochondria were indeed higher in HCT15-AA cells than in HCT15 cells, as revealed by transmission electron microscope (TEM). Simultaneously, mitochondrial DNA (mtDNA) was detected in higher copy numbers in CRC-AA cells than CRC cells (Fig. 1E). Furthermore, CRC-AA cells showed a significantly increase mitochondrial content, as quantified by flow cytometry of cells stained with Mito-tracker (Fig. 1F). We also measured mitochondrial respiratory complex components in CRC and CRC-AA cells using Western blot. As shown in Fig. 1G, the components of complex I - complex IV were upregulated in CRC-AA cells when compared to those in CRC cells. All these results indicated that colorectal cancer cells under acidic microenvironment had increased mitochondrial mass and fitness.

### Colorectal cancer cells adapted to acidic microenvironment are more reliant on oxidative phosphorylation

**Fig. 2:**
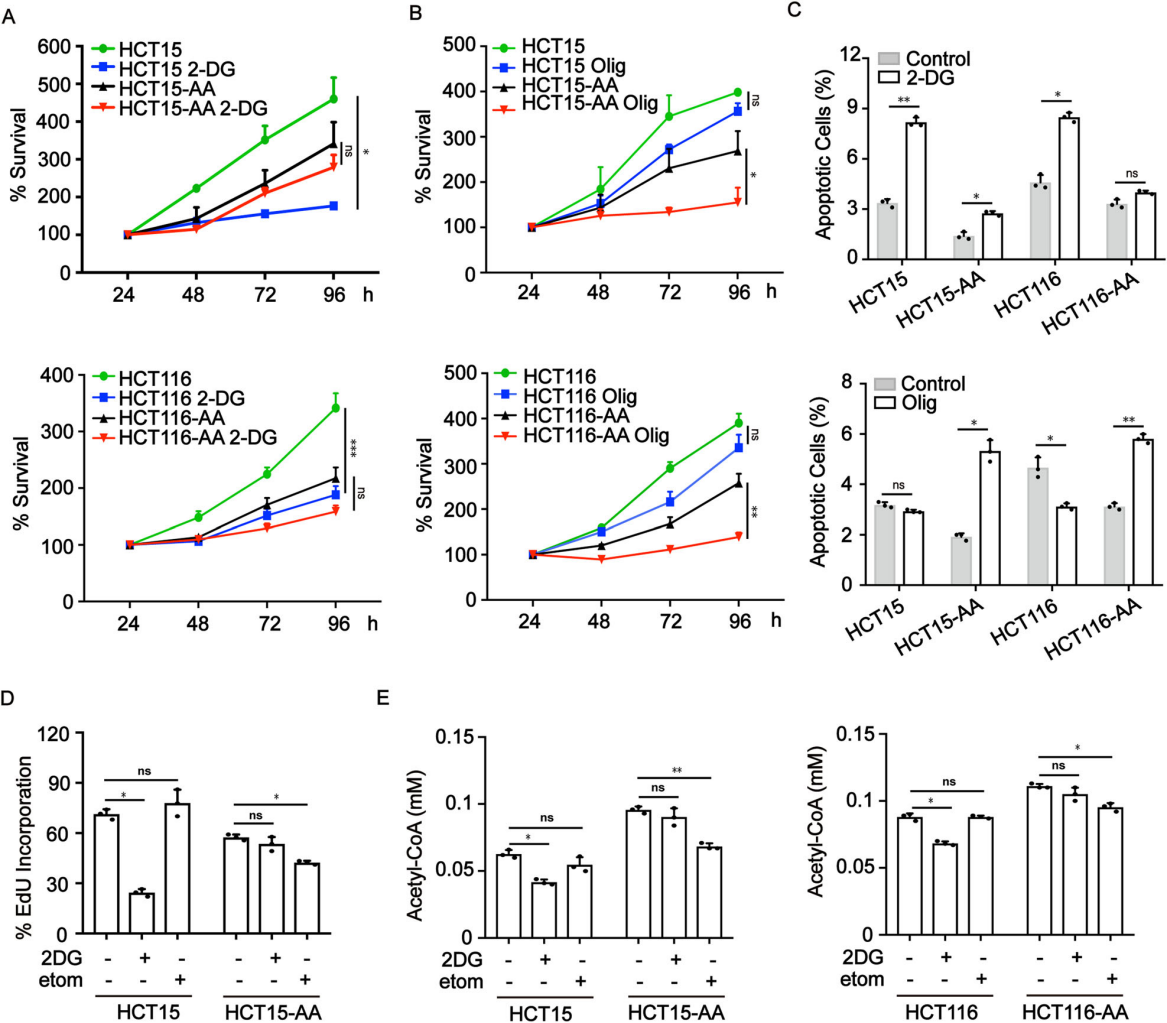
Colorectal cancer cells rely more on oxidative phosphorylation under acidic microenvironment. **A** Viability of CRC and CRC-AA cells treated with 2-DG (5 mM) measured by MTT assay. **B** Viability of CRC and CRC-AA cells treated with oligomycin (Olig, 1 μM) checked by MTT assay. **C** Apoptotic levels of CRC and CRC-AA cells treated with 2-DG (5 mM) or oligomycin (1 μM) checked by flow cytometry. **D** Proliferation of CRC and CRC-AA cells treated with 2-DG (5 mM), etomoxir (etom, 50 μM) for 48 h were determined by EdU assay. **E** The content of acetyl-CoA was detected in CRC and CRC-AA cells treated with 2-DG (5 mM) or etomoxir (50 μM) for 48 h. *p < 0.05, **p < 0.01, ***p < 0.001, ns indicates *p* > 0.05.

We next explored whether CRC-AA cells more rely on mitochondrial function for their survival in acidic milieu. We treated cancer cells with 2-Deoxy-D-glucose (2-DG), an inhibitor of glycolysis, or oligomycin A (Olig), an inhibitor of oxidative phosphorylation. While 2-DG dramatically inhibited the viability of HCT15 and HCT116 cells, as measured by MTT assay, it had no adverse effect on HCT15-AA and HCT116-AA cells (Fig. 2A). On the contrary, the viability of HCT15-AA and HCT116-AA cells was significantly inhibited after being treated with oligomycin A (Fig. 2B). The two types of cancer cells also exhibited differential sensitivity to 2-DG and Oligomycin A when measured by apoptosis assay. The level of apoptosis increased by 2-3 times in CRC cells treated with 2-DG, in comparison to mild or no increase in CRC-AA cells (Fig. 2C). Consistently, oligomycin A induced more apoptosis in CRC-AA cells (Fig. 2C). These results further supported that colorectal cancer cells more relied on OXPHOS, but not glycolysis, for survival under acidic microenvironment.

Since CRC-AA cells did not rely on glucose for their bioenergetic need (Fig. 2A–C), it is possible that they may generate acetyl-CoA, an important intermediate metabolite of tricarboxylic acid cycle, by FA oxidation in the mitochondria [23]. To test this, we used etomoxir, an inhibitor of mitochondrial carnitine palmitoyltransferase-1 (CPT-1), or 2-DG to treat CRC and CRC-AA cells and then assessed their proliferation and their production of acetyl-CoA. As expected, 2-DG drastically reduced the percentage of EdU positive cells in HCT15 cells but had no effect on HCT15-AA cells (Fig. 2D), indicating the high dependence on glycolysis for proliferation by HCT15 cells. Etomoxir, on the contrary, had no effect on HCT15 cells but significantly reduced the percentage of EdU positive cells in HCT15-AA cells (Fig. 2D). Correspondingly, 2-DG and etomoxir significantly reduced the production of acetyl-CoA in CRC and CRC-AA cells, respectively (Fig. 2E). These results indicated that the proliferation of CRC-AA cells was more dependent on FAO, as was the production of acetyl-CoA. This result is consistent with the enrichment of fatty acid metabolism pathway revealed by RNA-seq analysis (Supplementary Fig. 1A).

### Glutamine promotes cancer cell survival and proliferation under acidic microenvironment

**Fig. 3:**
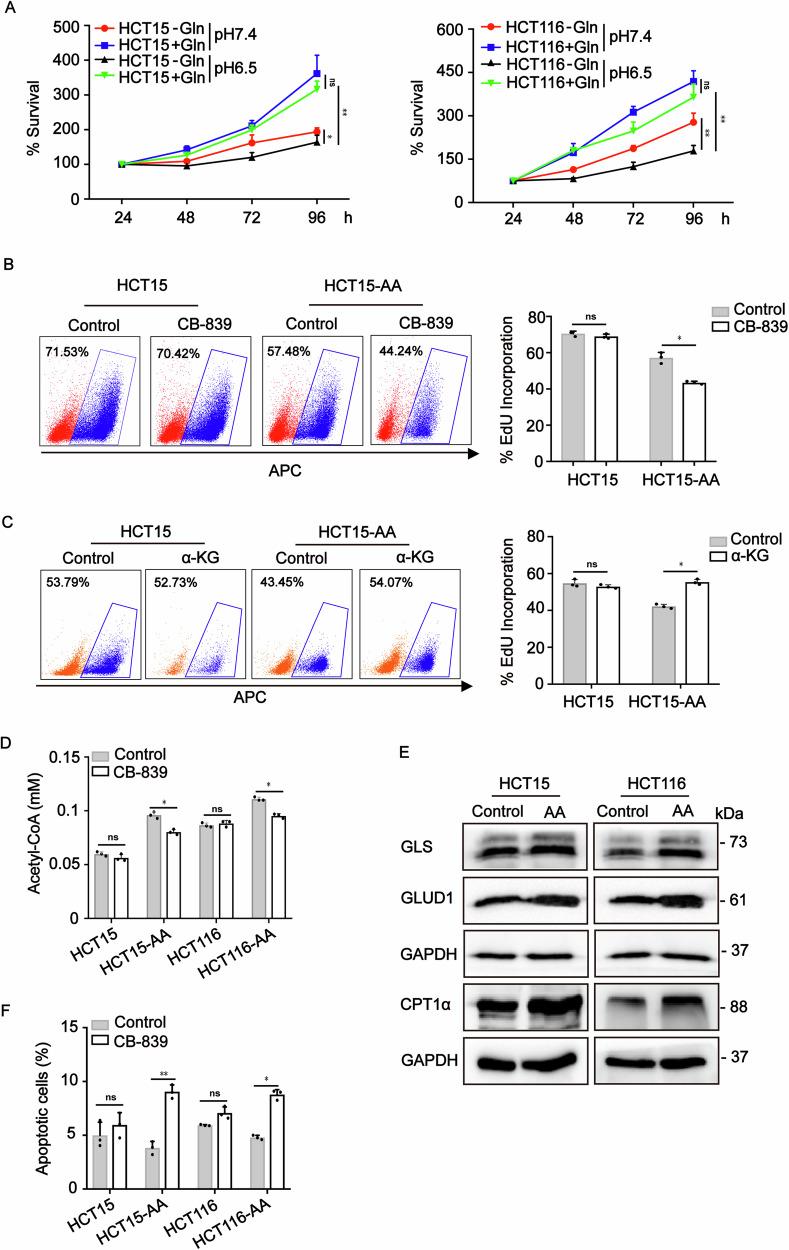
Colorectal cancer cells rely on glutamine to survival under acidic microenvironment. **A** Cells were cultured in glutamine-free medium (pH = 6.5 or pH = 7.4). Viability of CRC and CRC-AA cells checked by MTT assay. **B** Proliferation of CRC and CRC-AA cells treated with CB-839 (10 μM) for 48 h were determined by EdU assay. **C** Proliferation of CRC and CRC-AA cells treated with α-KG (1 μM) for 48 h were determined by EdU assay. **D** The content of acetyl-CoA was detected in CRC and CRC-AA cells treated with CB-839 (10 μM) for 48 h. **E** Western blot analysis of GLS, GLUD1 and CPT1 protein level in CRC and CRC-AA cells. GAPDH was used as a loading control. **F** Apoptotic levels of CRC and CRC-AA cells treated with CB-839 (10 μM) for 48 h checked by flow cytometry. *p < 0.05, **p < 0.01, ns indicates *p* > 0.05.

Glutamine is preferred to glucose when cancer cells are maintained at pH 6.5 and serves as an alternate source of acetyl-CoA [8]. It was also proposed to confer cancer cells acid resistance by releasing ammonia [24]. We observed that deprivation of glutamine in the medium could indeed reduce the viability of CRC-AA cells (Fig. 3A). Glutaminase inhibitor CB-839 also significantly reduced the incorporation of EdU in HCT15-AA, but not in the parental HCT15 cells (Fig. 3B). Consistently, adding α-ketoglutarate, a downstream product of glutamine, to the medium enhanced the proliferation of CRC-AA cells (Fig. 3C). CB-839 also inhibited the production of acetyl-CoA in CRC-AA, but not in the parental CRC cells (Fig. 3D). Notably, we observed that the expression of GLS and GLUD1 upregulated in CRC-AA cells when compared to CRC cells (Fig. 3E). Furthermore, CB-839 led to a more pronounced induction of apoptosis in CRC-AA than in parental CRC cells (Fig. 3F). These results indicate that α-ketoglutarate derived from glutamine metabolism may also support cancer cell survival and proliferation by serving as a source of acetyl-CoA.

### Mitochondria-derived reactive oxygen species participate in maintaining mitochondrial content in colorectal cancer cells under acidic microenvironment

**Fig. 4:**
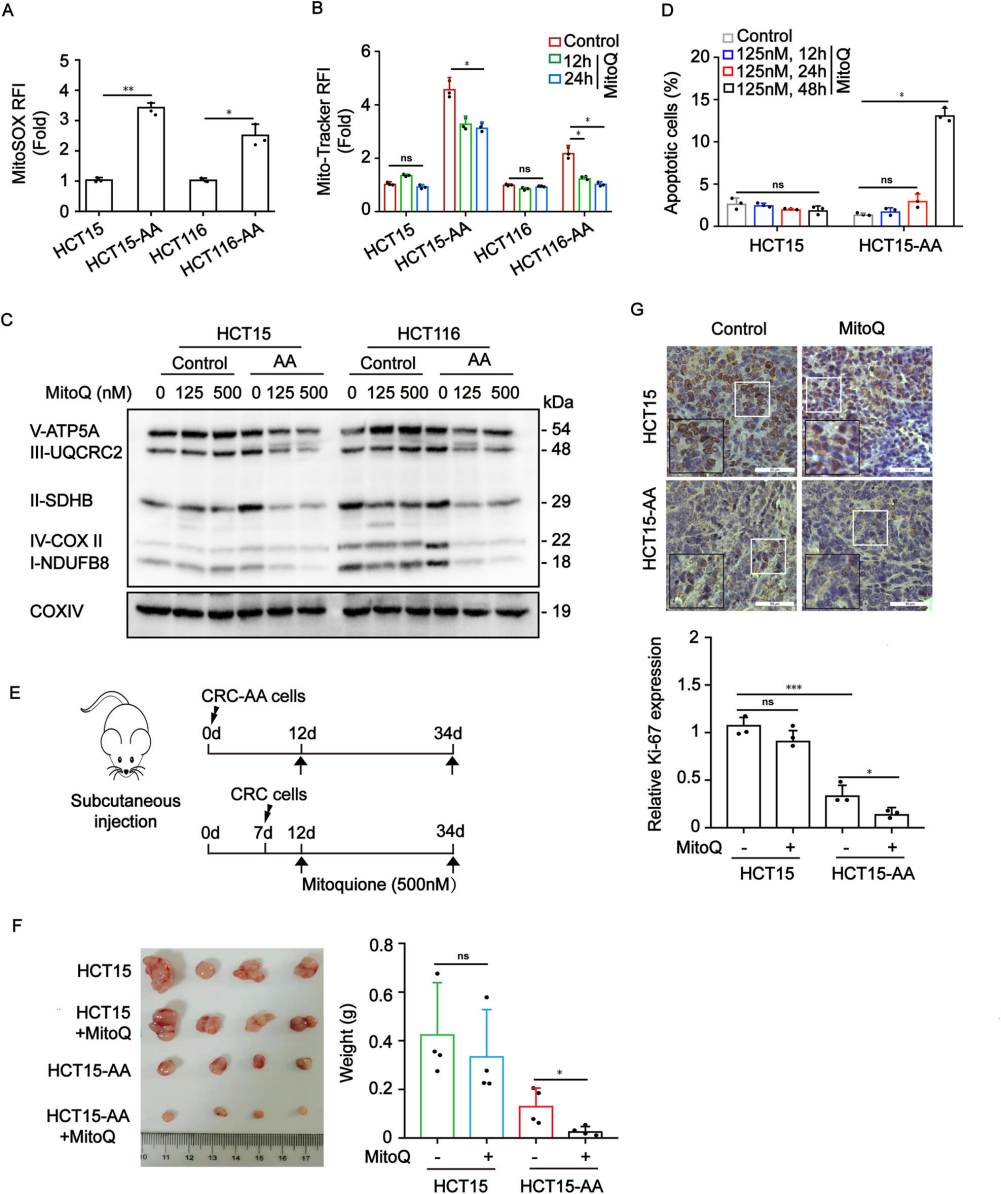
mtROS are essential for maintaining mitochondrial mass in colorectal cancer cells under acidic microenvironment. **A** MitoSOX distribution was measured by flow cytometry in CRC and CRC-AA cells. **B** Mitochondrial content was quantified by flow cytometry in CRC and CRC-AA cells treated with mitoQ (125 nM) from 12 h to 24 h detected by Mito-tracker. **C** Western blot analysis of OXPHOS protein level in CRC and CRC-AA cells treated with mitoQ (125 nM or 500 nM) from 24 h. COX IV was used as a loading control. **D** Apoptotic levels of CRC and CRC-AA cells after treatment with mitoQ (125 nM) from 12 h to 48 h. **E** Scheme for treatment paradigm of subcutaneous tumor xenografts. Because HCT15-AA cells are less proliferative than their parental cells, they were injected into nude mice 7 days earlier than their parental cells. 12 days later, the tumor-bearing mice were randomized into two groups: vehicle only (DMSO, *n* = 4), mitoQ only (500 nM, *n* = 4, po.). Tumor weights were measured at day 34. **F** Tumor xenografts formed by HCT15-AA are more sensitive to mitoQ. **G** Representative images of immunohistochemistry staining using Ki-67 antibody in tumors for each group. Scale bar: 50 μm. *p < 0.05, **p <0.01, ***p < 0.001, ns indicates *p* > 0.05.

Considering that mitochondrial superoxide (O_2_·^−^) serves as an important signal for mitochondrial biogenesis, we measured the levels of O_2_·^−^ in CRC-AA and their parental cells by using MitoSOX Red as a probe. We observed elevated mtROS levels in CRC-AA cells when compared to their parental cells (Fig. 4A). To determine whether the elevation in mtROS contributes to mitochondrial accumulation in CRC-AA cells, we applied mitochondrial antioxidants-mitoquinone (MitoQ) to quench O_2_·^−^ and then measured mitochondrial mass in CRC-AA cells. Interestingly, MitoQ (125 nM) significantly decreased the mitochondrial mass in CRC-AA cells, as measured by Mito-tracker at 12 and 24 h after treatment (Fig. 4B). Moreover, the components of OXPHOS complexes were also reduced in HCT15-AA and HCT116-AA cells treated with MitoQ at 125 or 500 nM for 24 h (Fig. 4C). The percentage of apoptotic cells increased several folds when CRC-AA cells were treated with MitoQ for 48 h (Fig. 4D and Supplementary Fig. 2B). However, increasing MitoQ concentration to 1 μM failed to decrease mitochondrial mass (Supplementary Fig. 2A), which was associated with massive cell death. In addition, we found that quenching O_2_·^−^ impaired the autophagic flux in HCT15-AA cells (Supplementary Fig. 2C). Next, we determined whether MitoQ could reduce the proliferation of colorectal cancer cells under acidic microenvironment in vivo. We established a xenograft tumor model in nude mice with HCT15-AA and their parental cells (Fig. 4E). The results showed that tumors formed by HCT15-AA cells were more responsive to MitoQ than those by the HCT15 cells (Fig. 4F). Consistently, there was a significant decrease in the abundance of Ki-67 positive cells in HCT15-AA tumors treated with MitoQ (Fig. 4G). These results strongly suggest that mitochondria-derived reactive oxygen species participate in maintaining mitochondrial mass and sustain tumor cell proliferation.

AMPKα, a cell energy sensor, regulates mitochondrial function and biogenesis by upregulating PGC-1α (Peroxisome proliferator-activated receptor gamma coactivator) expression. PGC-1α functions as a co-activator of transcription to drive mitochondrial biogenesis [25, 26]. We observed that AMPKα phosphorylation and PGC-1α expression were increased in CRC-AA cells (Supplementary Fig. 2D). Furthermore, CRC and CRC-AA cells treated with MitoQ (125 nM) exhibited a remarkable decrease in the phosphorylation of AMPK in HCT15-AA cells (Supplementary Fig. 2E), indicating that mitochondrial reactive oxygen species may sustain the AMPK activity and thereby promote mitochondrial biogenesis.

### Autophagy-derived lipid droplets sustain mitochondrial mass and fitness

**Fig. 5:**
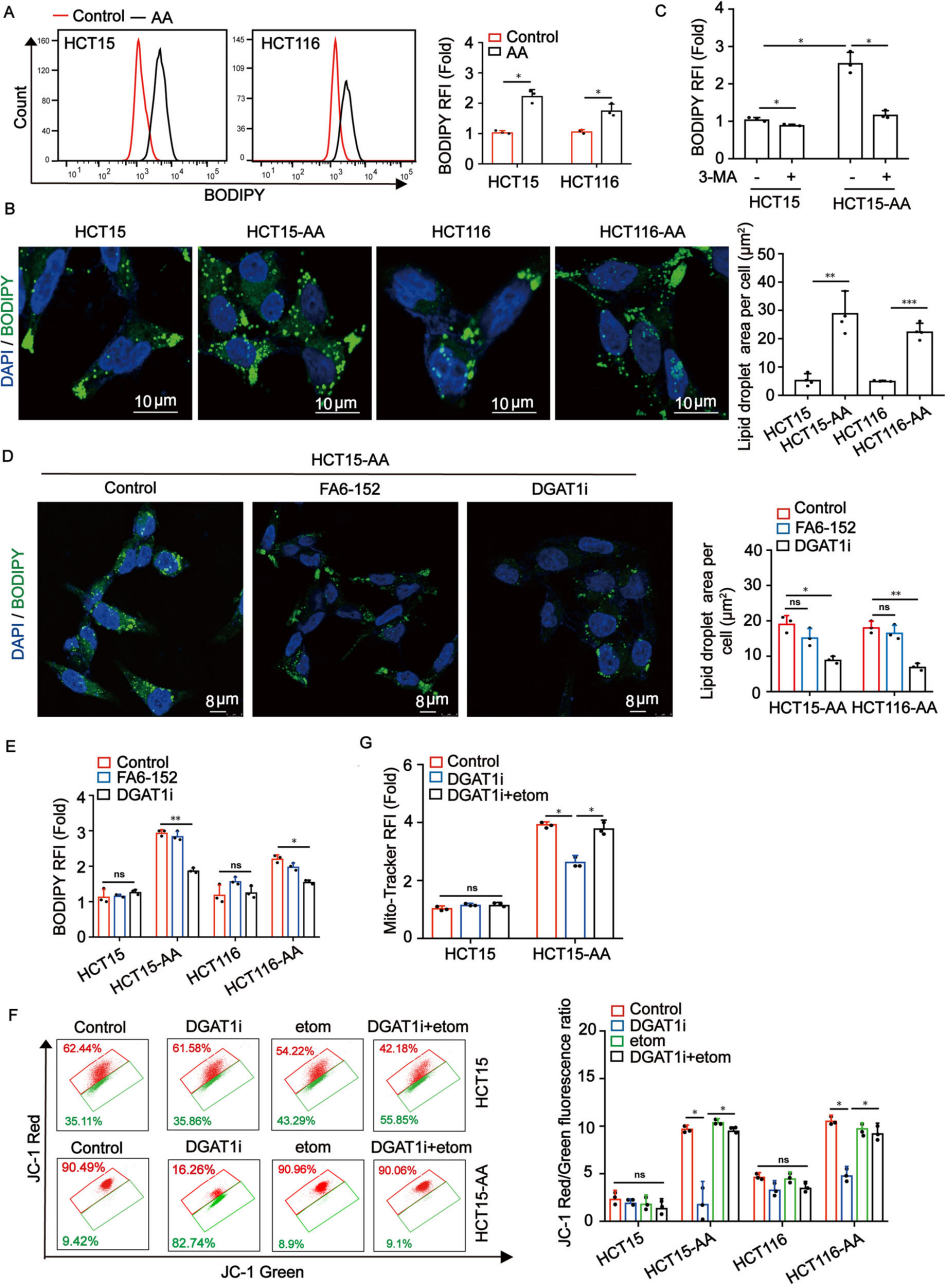
Autophagy-dependent lipid droplets maintain mitochondrial mass and fitness in colorectal cancer cells under acidic microenvironment. **A** BODIPY (493/503) staining was used to detect lipid droplets content in CRC and CRC-AA cells by flow cytometry. **B** BODIPY staining was used to detect lipid droplets content in CRC and CRC-AA cells by immunofluorescence. Scale bar: 10 μm. **C** BODIPY (493/503) staining was used to detect lipid droplets content in CRC-AA cells treated with 3-MA by flow cytometry. **D** BODIPY staining was used to detect lipid droplets content in CRC-AA cells treated with DGAT1 inhibitor (DGAT1i, 10 μM) or anti-CD36 antibody (FA6-152) by immunofluorescence. Scale bar: 8 μm. **E** BODIPY staining was used to detect lipid droplets content in CRC-AA cells treated with DGAT1 inhibitor or FA6-152 by flow cytometry. **F** Measurements of MMP (by JC-1) in CRC and CRC-AA cells treated with DGAT1 inhibitor or etomoxir (20 μM) for 24 h. **G** Mitochondrial content was quantified by flow cytometry in CRC and CRC-AA cells treated with DGAT1 inhibitor or etomoxir for 24 h. * *p* < 0.05, **p < 0.01, ***p < 0.001, ns indicates *p* > 0.05.

Lipid droplets (LDs) function as a lipid buffering system to prevent acylcarnitine accumulation and lipotoxicity to mitochondria during the autophagic degradation of membranous organelles [20]. Considering that there is a high autophagic flux in CRC-AA cells [27], we therefore determined whether CRC-AA cells also harbor more LDs than their parental cells. We used BODIPY (493/503) to stain LDs in CRC and CRC-AA cells and observed that CRC-AA cells exhibited approximately 2-fold more LDs than in CRC cells (Fig. 5A, B). To determine whether the autophagic flux contributes to LD formation, we applied 3-MA, an inhibitor of autophagy, to CRC-AA cells and then measured the amounts of LDs. The fluorescence intensity of LDs indeed decreased in CRC-AA cells in the presence of 3-MA (Fig. 5C). Furthermore, when lipid uptake was blocked using an antibody against CD36, which is responsible for lipid uptake, the level of LDs was not affected (Figs. 5D, 5E and Supplementary Fig. 3A), suggesting that the LDs were not formed from external sources. Since the synthesis of triglycerides, the main component of lipid droplets, is catalyzed by DGAT1/2 (diacylglycerol O-acyltransferase homolog 1/2), we examined the mRNA expression of DGAT1 and DGAT2 in CRC-AA and their parental cells (Supplementary Fig. 3B). As a positive control, the LDs were remarkably reduced when CRC-AA cells were treated with a DGAT1 inhibitor, A922500 (Fig. 5D, E and Supplementary Fig. 3A).

We next determined whether the LDs may sustain mitochondrial function by supplying fatty acids. Inhibition of FA release from LDs by blocking the activity of adipose triglyceride lipase (ATGL) with atglistatin reduced ATP level in CRC-AA cells (Supplementary Fig. 3C). These data showed that LDs may serve as fuel source for mitochondrial oxidative respiration in CRC-AA cells.

We further characterized mitochondrial fitness in CRC-AA cells in which LD biogenesis is blocked by the DGAT1 inhibitor A922500. We observed a significant decrease in mitochondrial membrane potential (MMP) in CRC-AA cells, but not in CRC cells, when treated with A922500 (Fig. 5F). Meanwhile, inhibition of DGAT1 strongly reduced mitochondrial content, as revealed by Mito-tracker staining in CRC-AA cells (Fig. 5G and Supplementary Fig. 3D). In contrast, ATGL inhibitors had no effect on mitochondrial content in CRC-AA cells (Supplementary Fig. 3D). We speculated that when DGAT1-mediated formation of LDs is inhibited in CRC-AA, the free fatty acids in cytoplasm would be in excess and be toxic to mitochondria. To test this, we applied Etomoxir to block the entry of fatty acid derivatives to mitochondria. Etomoxir applied alone had no effect on mitochondrial membrane potential in CRC-AA cells (Fig. 5F). However, depolarized MMP and decreased mitochondrial content caused by DGAT1 inhibitor were rescued by Etomoxir (Fig. 5F, G and Supplementary Fig. 3E). Thus, our results indicated that LDs provided a lipid buffering system to reduce lipotoxicity during enhanced autophagy to maintain mitochondrial mass and fitness in CRC-AA cells.

### Positive correlation of mitochondrial contents and lipid droplets in vivo

**Fig. 6:**
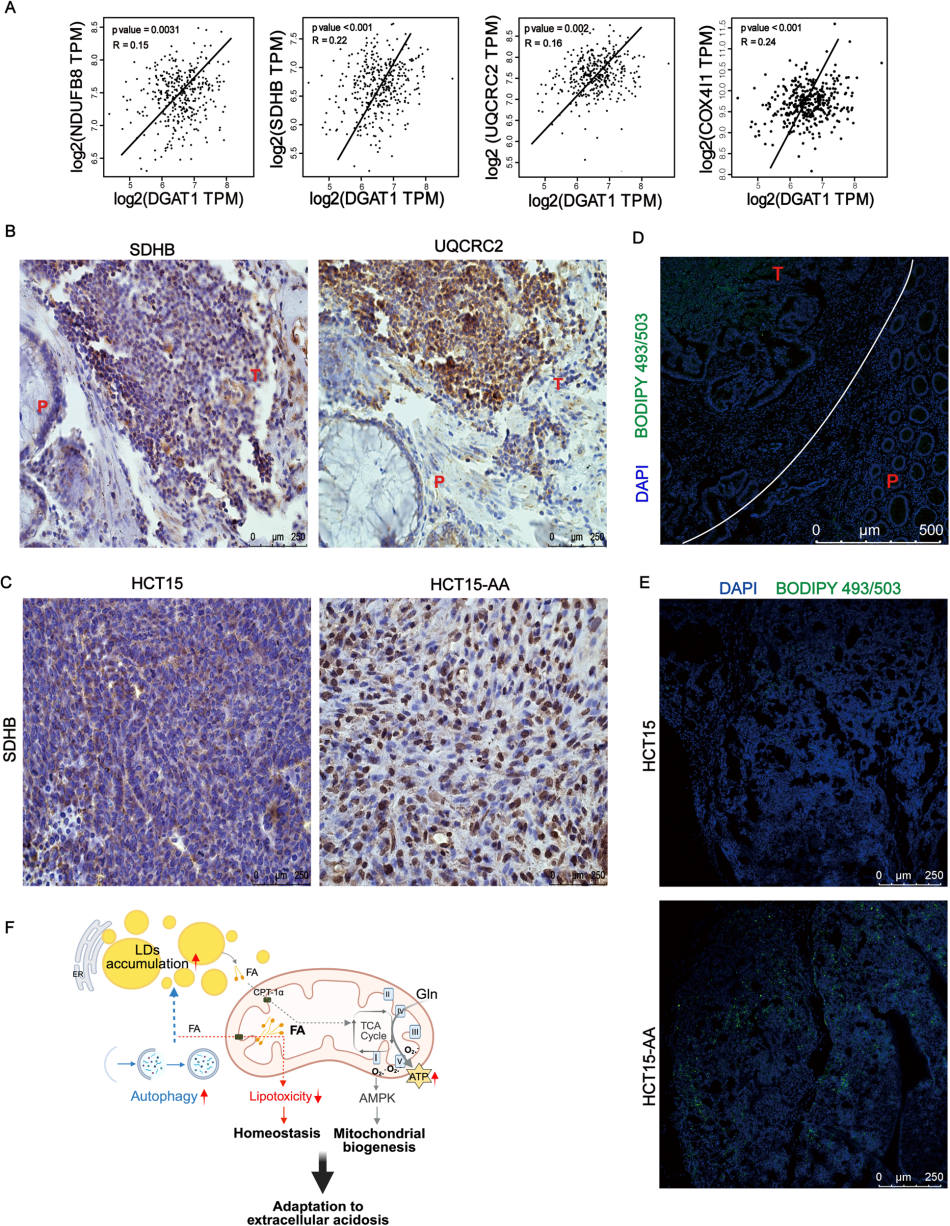
Mitochondria supports tumor growth in vivo. **A** Correlation between OXPHOS pathway genes and DGAT1 in colon adenocarcinoma samples. **B** Representative IHC images showing the SDHB and UQCRC2 in colon adenocarcinomas (T, tumor; P, para-carcinoma). Scale bar: 250 μm. **C** Representative IHC images showing the SDHB in HCT15 and HCT15-AA tumors. Scale bar: 250 μm. **D** Representative immunofluorescent pictures of BODIPY (493/503) and DAPI staining in colon adenocarcinomas (T, tumor; P, para-carcinoma). Scale bar: 500 μm. **E** Representative immunofluorescence images of BODIPY (493/503) and DAPI staining in HCT15 and HCT15-AA tumor grafts. Scale bar: 250 µm. **F** A schematic model showing the relationship between mitochondria and LDs in CRC-AA cells.

We next analyzed TCGA database (http://gepia.cancer-pku.cn/index.html) to seek information on possible association between mitochondrial content and the abundance of lipid droplets in colorectal cancer in vivo. Among various solid tumors, DGAT1 has the highest expression level in colorectal adenocarcinoma (supplementary Fig. 4A). The OXPHOS pathway genes-NDUFB8, SDHB, UQCRC2, COX4I1 are positively correlated with DGAT1 (Fig. 6A). Next, we examined the expressions of genes encoding mitochondrial components (SDHB, UQCRC2) and LDs in tumor tissues of colorectal cancer patients or in tumor grafts. Immunohistochemistry showed that the levels of SDHB and UQCRC2 in tumor region (T) were higher than those in para-carcinoma (P) region in patients (Fig. 6B). Similarly, the levels of SDHB and UQCRC2, as examined by immunohistochemistry, were also increased in HCT15-AA tumor grafts compared with HCT15 tumor (Fig. 6C and supplementary Fig. 4B). In tumor tissues derived from patients, BODIPY (493/503) staining results showed that the content of LDs in tumor region (T) was higher than that in para-carcinoma region (P) (Fig. 6D). HCT15-AA tumor grafts also accumulated much more LDs as detected using BODIPY (493/503) staining (Fig. 6E). All these results indicated a positive correlation between mitochondria and LDs in tumor cells in vivo.

## Discussion

Compared with normal cells, tumor cells possess high metabolic flexibility and are usually reprogrammed to support the proliferation or survival under stressful conditions [28]. Acidic microenvironment profoundly changes the transcriptomic characteristics of tumor cells that are related to proliferation, metastasis and survival [29]. Cancer cells tend to rely more on mitochondria under adverse conditions. Lactic acid triggers morphological reorganization of mitochondria, thereby reconfiguring mitochondrial bioenergetics to allow effective oxidative phosphorylation [5]. Acidosis can even override oxygen deprivation to maintain mitochondrial function and cell survival [14]. Dormant breast cancer cells exhibit upregulated AMPK to promote mitochondrial biogenesis and FAO [30]. Consistently, we here show that colorectal cancer cells exposed to chronic acidosis acquire an increased mitochondrial mass and fitness. Importantly, the increased mitochondrial fitness in CRC-AA cells requires a buffering system of LDs. LDs are derived from the endoplasmic reticulum, a phospholipid monolayer surrounding neutral lipids (triglycerides and cholesterol esters) [31]. As dynamic hubs of intracellular lipid metabolism LDs prevent excessive polyunsaturated fatty acids (PUFA) from causing lipotoxicity to cells [21]. CPT1α catalyzes the transfer of the long-chain acyl group in acyl-CoA ester to carnitine and thus allows fatty acids to enter the mitochondrial matrix for FAO. We observed that while DGAT1 inhibition damages mitochondria, presumably due to increased uptake of free fatty acids that are in excess in the absence of LD-mediated buffering capacity, this mitochondrial defect can be rescued by Etomoxir, which inhibits CPT1α. This finding provides further support to the notion that LDs function as a buffering system that protects mitochondria from lipotoxicity. In addition, CRC-AA cells also rely more on FAs as the source of acetyl-CoA, which is consistent with the reports that LDs are formed to store the autophagic products and are mobilized to provide “on-demand” supply of FA to maintain cell viability through FAO During nutrient deficiency [20, 32, 33]. Our finding is in contrast to the report that TGF-β2 increases CD36 translocation to promote FA uptake under acidic microenvironment [10]. Considering that CRC-AA cells exhibit a high autophagic flux and accumulate abundant LDs, we are tempted to believe that they may not need to uptake more exogenous FAs. While free FAs in excess may increase the likelihood of lipid peroxidation and cell death, we did not observe significantly increased lipid peroxidation when LD biogenesis was blocked (Supplementary Fig. 4C). We previously report that GPX4, a critical inhibitor of lipid peroxidation and ferroptosis, is highly expressed in CRC-AA cells [34], which might have protected them from lipid peroxidation. It has been shown that intratumoral lipid metabolic reprogramming acts as a pro-tumoral regulator in the tumor milieu. Alterations in lipid metabolism, lipogenesis, and cholesterol synthesis contribute to the generation of signaling molecules, such as phosphatidylinositols, which may alter the activities of the immune components [35], revealing the potential for developing new drugs based on FA metabolism in cancer treatment.

As in many cases where ROS are necessary to maintain cell proliferation and function [36], we found that mitochondrial ROS were elevated in CRC-AA cells and were essential for AMPK activation and mitochondrial fitness. Quenching of mtROS did not only decrease MMP but also inhibited tumorigenic ability of CRC-AA cells. This essential role of mtROS in maintaining mitochondrial homeostasis and fitness in CRC-AA cells is similar to that of mtROS in mediating the anti-inflammatory polarization of macrophages induced by calorie-restriction mimetics spermidine [37]. Mitochondrial superoxide and H_2_O_2_ were markedly elevated in macrophages treated with spermidine and were responsible for the activation of AMPK, increase of mitochondrial mass and the induction of autophagy, which are all required for spermidine to exert its anti-inflammatory function. Adipogenesis and thermogenesis are also driven by mtROS [38, 39]. These results suggest that mtROS play a critical role in sustaining mitochondrial fitness and metabolism in a broad spectrum of biological contexts.

Acidic tumor microenvironment does not only enhance the plasticity of tumor cells, but also affects the fate and function of immune cells within the microenvironment. It is recognized that acidic microenvironment can hinder the maturation of antigen-presenting cells and the activation of lymphocytes, thereby forming an immunosuppressive microenvironment [40]. PGE2 were recently shown to inhibit the expansion of tumor-infiltrating lymphocytes [41, 42]. Therefore, reshaping acidic microenvironment may alleviate immune suppression. Interestingly, lipid droplet serves as the site of PGE2 synthesis in colon cancer cells [43]. Therefore, further exploration of the impact of acidic microenvironment on lipid droplet formation in cancer cells and immune response is of great significance for developing effective anti-tumor imunotherapeutics.

In short, this study reveals a cascade of autophagy, LD biogenesis and mitochondrial fitness that is essential for cancer cell survival under chronic acidic microenvironment. Strategies to interfere with this cascade may help eliminate malignant cancer cells adapted to acidosis.

## Materials and methods

### Cell culture

Human colorectal cancer (CRC) cell lines HCT15 and HCT116 were obtained from the Cell Bank of Chinese Academy of Sciences (Shanghai). The cells were cultured in RPMI-1640 (pH 7.4), supplemented with 10% fetal bovine serum (FBS), 100 U/mL penicillin, 100 μg/mL streptomycin. Cells were maintained in a 5% CO_2_/95% air incubator at 37 °C. Acidic medium was prepared by adjusting the pH adjusted to 6.5 with 25 mmol/L each of PIPES and HEPES. CRC-AA cells (HCT15-AA and HCT116-AA) were obtained by continuously culturing and passing the CRC cells in acidic medium for at least three months. During this period, cells were passaged 24 times. Cells used in the experiments are of the same passage number.

### Reagents and antibodies

Oligomycin A (S1478), CB-839 (S7655), Etomoxir (S824405), DGAT1 inhibitor (A922500, S2674) were purchased from Selleck. 2-Deoxy-D-arabino-hexose (HY-13966), Mitoquinone mesylate (HY-100116A), MitoSOX™ Red Mitochondrial Superoxide Indicator (M36008), BODIPY 581/591 (D3861) was purchased from Invitrogen Life Technologies. Mitochondria isolation kit (C3601), Mito-tracker Green (C1048), Enhanced ATP Assay Kit (S0027), Mitochondria staining kit (JC-1) (C2003s), EdU Cell Proliferation Kit (C0081S), DAPI (C1002) were purchased from Beyotime (China). Atglistatin (SML1075) was purchased from Sigma-Aldrich. Antibody against total OXPHOS (ab110411), PGC-1α (ab77210), Ki-67 (ab15580) were purchased from Abcam. Anti-phospho-AMPKα (Ser485) (2537), Anti-AMPKα (5831), Anti-GAPDH (5174 s), Anti-rabbit IgG (7074S) were from Cell Signaling Technology. Anti-SDHB (sc-271548), Anti-UQCRC2 (sc-390378) were from Santa Cruze. Anti-GLUD1 (14299-1-AP), Anti-GLS (12855-1-AP), Anti-CPT1α (15184-1-AP) were from Proteintech. BODIPY 493/503 (25893) was purchased from Cayman.

### RNA-seq analysis

RNA-seq analysis of HCT15 and HCT15-AA cells, three replicates for each sample, was performed by Biomarker (Beijing, China). The data were analyzed by gene set enrichment analysis (GSEA).

### Measurement of oxygen consumption rate

The real-time measurement of oxygen consumption rate (OCR) in live cultured cells was performed using the Seahorse XF24 Analyzer (Seahorse Bioscience, North Billerica, MA, USA) according to the manufacturer’s instructions. ATP synthase inhibitor oligmycin (1 μM), uncoupler FCCP (4 μM) and complex I inhibitors rotenone (1 μM) and antimycin A (1 μM) were injected to the wells, followed by measurement cycles for OCR. The oxygen consumption rates were calculated from the continuous average slope of the O_2_ decrease using a compartmentalization model.

### Determination of mitochondrial membrane potential (MMP)

The mitochondrial membrane potential (MMP) was determined by Mitochondria Staining Kit. Briefly, cells were harvested and loaded with JC-1 (2 μM) at 37 °C, 5% CO_2_ for 20 min, then analyzed by flow cytometry excited at 488 nm and the emitted fluorescence was collected at 530 nm and 590 nm. MMP was expressed as the emitted fluorescence ratio (590/530 nm) in percentage to the initial level.

### Electron microscopy

Cells were harvested and fixed for 24 h at 4 °C in 3% pentanediol. Cells were washed with phosphate buffer and subsequently post-fixed in 1% (v/w) osmium tetroxide (Merck). Samples were dehydrated by successive passages in increasing concentrated ethanol baths (30, 50, 70, 85, and 100%). After embedding in epon resin LX 112 (Ladd Research Industries), ultra-thin sections of cell-covered filters were prepared using an 8800 ultrotome III (LKB). TEM analysis used TEM grids (Agar Scientific) covered with non-porous formvar.

### Measurement of mitochondrial mass

Mitochondrial mass was measured using Mito-tracker green probe (Beyotime, China) according to the manufacturer’s protocols. Cells were harvested and washed with PBS, and then stained with 100 nM Mito-tracker green probe for 20 min at 37 °C and 5% CO_2_ in the dark. After treatments, cells were washed three times with PBS and measured by flow cytometry (BD Biosciences, CA). At least 10,000 cells were collected.

### Determination of mtDNA copy number by real-time quantitative PCR

Total genomic DNA was extracted from cells with a genomic DNA extraction kit (Vazyme, China). For the determination of mtDNA copy number relative to nucleus DNA in cells, the ND1 gene was used as a marker of mtDNA and the B2M gene for nucleus DNA [44]. ND1, forward: 5′-TCGCCATCATATTCGTAGGAG-3′, reverse: 5′-GTAGCGTCGTGGTATTCCTGA-3′. B2M, forward: 5′-TTAACGTCCTTGGC TGGGTC-3′, reverse: 5′-ACTGGAAGACAAAGGGCTCG-3′.

### Western blot analysis

Cells were harvested and lysed in Cell Lysis Buffer (Beyotime, China) for 30 min. Protein concentrations of the lysates were determined by the BCA protein assay system (Invitrogen Life Technologies). Equal amounts of protein were separated by 10% SDS-PAGE, transferred to PVDF membrane (Millipore, Billerica, MA), and blocked with 5% nonfat dry milk in TBS-Tween 20 (0.1%, v/v) for 1 h at room temperature. The membrane was incubated with primary antibody overnight at 4 °C. After washing, the membrane was incubated with the appropriate horseradish peroxidase secondary antibody for 1 h. Following three washes, the blots were developed by ECL kit (NCM Biotech, China). The full length uncropped original western blots were shown in Supplementary Materials.

### Measurement of MitoSOX

Mitochondrial ROS were measured using MitoSOX ™ Red mitochondrial superoxide indicator (Invitrogen) according to the manufacturer’s instructions. Cells were washed and harvested in PBS, and then stained with 1 μM MitoSOX Red for 20 min at 37 °C and 5% CO_2_ in the dark. Samples were subsequently washed using ice-cold PBS and centrifuged for 5 min at 1500 rpm before being resuspended in ice-cold PBS and kept on ice until analysis. Flow cytometry was performed using a BD Biosciences FACScan II cytometer. At least 10,000 cells were collected.

### Cell viability assay

Cells were plated in 96-well cell culture plates at the concentration of 2000–3000 cells/well. After 24 h, the medium was removed and replaced with fresh medium with or without drugs. Cell viability was measured by MTT assay kit (Beyotime, China) following manufacturer’s instructions. The absorbance of converted dye is measured at the wavelength of 490 nm and the absorbance is proportional to cell viability.

### Flow cytometry analysis of apoptosis

Apoptotic cells were determined using the Annexin V/Dead Cell Apoptosis Kit (Invitrogen). Cells were harvested using 0.25% Trypsin-EDTA, centrifuged (300 g), and washed twice in PBS. Cells were resuspended in 100 μL of 1× binding buffer at a density of 1 × 10^6^ cells/mL and incubated in the dark with APC annexin-V and 7-AAD. Cell fluorescence was assessed in a FACScan flow cytometer (Becton Dickinson, San Jose, CA, USA).

### Tumor xenograft

Four-weeks-old male nude mice were purchased from Nanjing Experimental Animal Center and kept in pathogen-free conditions and handled in accordance with the requirements of the Guideline for Animal Experiments. Half of mice were subcutaneously inoculated with 8 × 10^6^ CRC-AA cells (suspended in 100 µL PBS). After 10 days, the other mice were subcutaneously inoculated with 8 × 10^6^ CRC cells (suspended in 100 µL PBS). Mice were randomized into two treatment groups: vehicle only (DMSO, *n* = 4, po.), Mitoquinone only (500 nM, *n* = 4, po.). Tumor weights were measured at day 34. The animal studies were approved by the Animal Ethics Committee of Soochow University. All methods were performed in accordance with the relevant guidelines and regulations.

### Immunohistochemistry

Formalin-fixed and paraffin-embedded tumor xenografts or tissue were sectioned at 4 μm. After deparaffinizayion and rehydration, the sections were boiled in citrate sodium buffer (pH 6.0) for 15 min for antigen recovery and immersed in 3% H_2_O_2_ for 10 min to quench endogenous peroxidase. Nonspecific binding was blocked by 10% donkey serum at room temperature for 1 h. Then sections were incubated with the primary antibodies overnight at 4 °C. The signal was developed by DAB detection system and counterstained with hematoxylin.

### Acetyl-CoA measurements

Acetyl-CoA concentration in cellular extracts was determined by using the PicoProbe Acetyl-CoA assay kit from Abcam (ab87546), according to manufacturer’s instructions. Briefly, protein was removed in the sample using the perchloric acid protocol and the supernatant was neutralized with 3 M KHCO_3_. The CoASH quencher and quencher remover were added into the sample to correct the background generated by free CoASH and succ-CoA. The sample was then diluted with the reaction mix, and the fluorescence signal was measured at Ex/Em = 535/589 nm. The relative acetyl-CoA concentration was normalized with the mitochondrial protein.

### EdU proliferation assay

Cells proliferation was evaluated using 5-ethynyl-2′-deoxyuridine (EdU) cell proliferation assay kit (Beyotime, China). Briefly, the cells were incubated with 50 μM EdU for 2 h before fixation, permeabilization and EdU staining according to the manufacturer’s protocol. The proportion of EdU positive cells was determined by flow cytometry.

### Immunofluorescence staining of BODIPY

Cells grown on cover slips in 6-well plate were washed in PBS twice and were fixed in 4% paraformaldehyde for 15 min at room temperature. Cells were added at a dilution of BODIPY 493/503 (1:5000) in PBS and incubated for 20 min. Cells were washed four times in PBS. The nuclei were counterstained in DAPI and were mounted with nail polish. Slides were then examined under a fluorescence microscope. Fluorescence microscopy images were quantified using ImageJ software. The area of BODIPY stained LDs were quantified from three independent experiments.

### Quantitative RT-PCR

Total RNA was collective by using TRIzol reagent (Invitrogen, China) according to the manufacturer’s protocol. cDNA was synthesized by reverse transcription of 1 μg of total RNA with random hexamers. Real-time quantitative PCR was performed using the Light Cycler® 480 sequence Detection System (Roche Applied Science, Germany) with SYBR-Green (Invitrogen). The primer sequences are listed in Supplementary Table 3. Each assay was normalized to the level of GAPDH mRNA.

### Tissue samples

All colorectal cancer tissues were collected in the First Affiliated Hospital of Soochow University from September 2021 to December 2021. All participants gave written informed consent. The studies were approved by the Ethics Committee of Soochow University. All methods were performed in accordance with the relevant guidelines and regulations. Details were listed in Supplementary Table 2.

### Immunofluorescence

Tissue samples were embedded in OCT, and then was sectioned at 8 μM. The slides were fixed in 4% paraformaldehyde for 10 min at room temperature and were washed in PBS twice. BODIPY (493/503) was added at a dilution of 1:100 in PBS and incubated for 20 min. Cells were washed three times in PBS. The nuclei were counterstained in DAPI and were mounted with nail polish. Slides were then examined under a fluorescence microscope.

### Analysis of TCGA data

The results in Fig. 6A were generated by using GEPIA (http://gepia.cancer-pku.cn/index.html).

### Statistical analysis

All statistical data were calculated and graphed using GraphPad Prism 8 and data were presented as mean ± standard deviation (SD). Statistical significance was tested using two-sided Student’s t-test. *p* < 0.05 was considered statistically significant. Statistical significance was also taken as *p < 0.05, **p < 0.01 and ***p < 0.001, ns stands for no significant.

## Supplementary information

Supplementary Figures

Supplementary Figure Legends

Supplementary Table 1, 2, 3

Original Western Blot

## Data Availability

The raw data used to support the findings of this study are available from the corresponding author upon request.
