# Supplementary material for: Autophagic flux-lipid droplet biogenesis cascade sustains mitochondrial fitness in colorectal cancer cells adapted to acidosis
Source: Cell Death Discov. 2025 Jan 25;11:21. doi: 10.1038/s41420-025-02301-6 (PMC11761495; doi:10.1038/s41420-025-02301-6)

Supplementary Figure 1

A

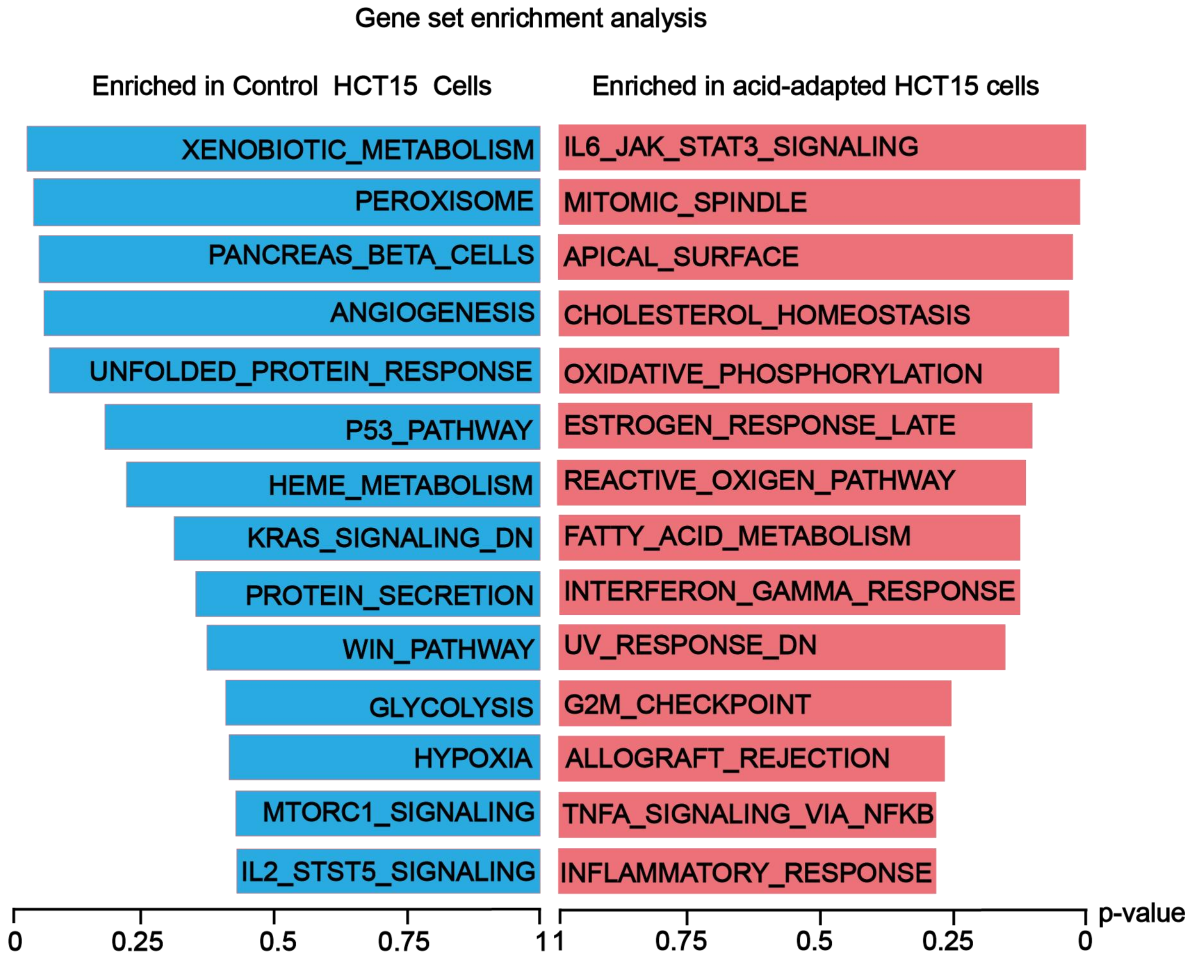

Supplementary Figure 2

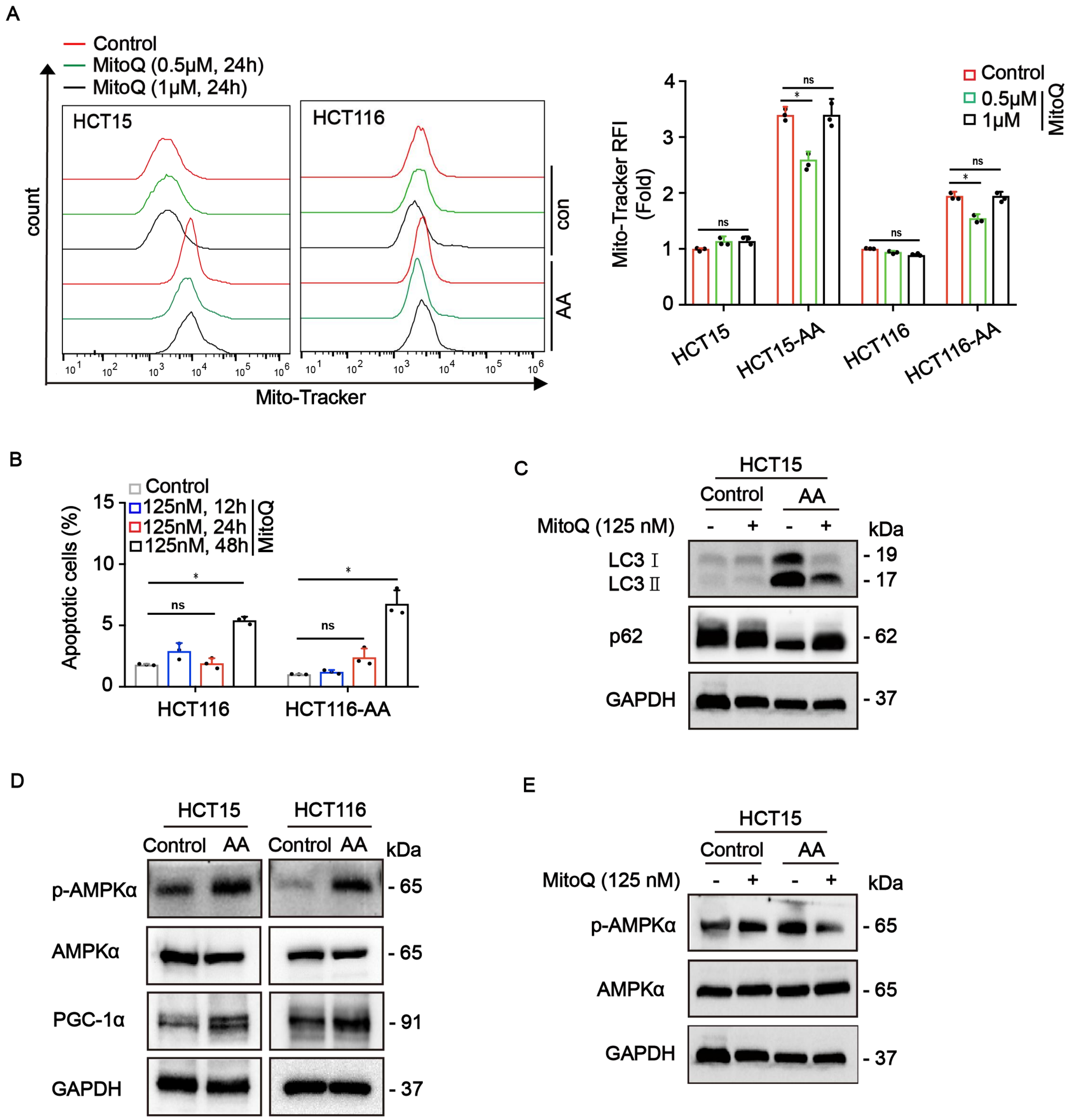

Supplementary Figure 3

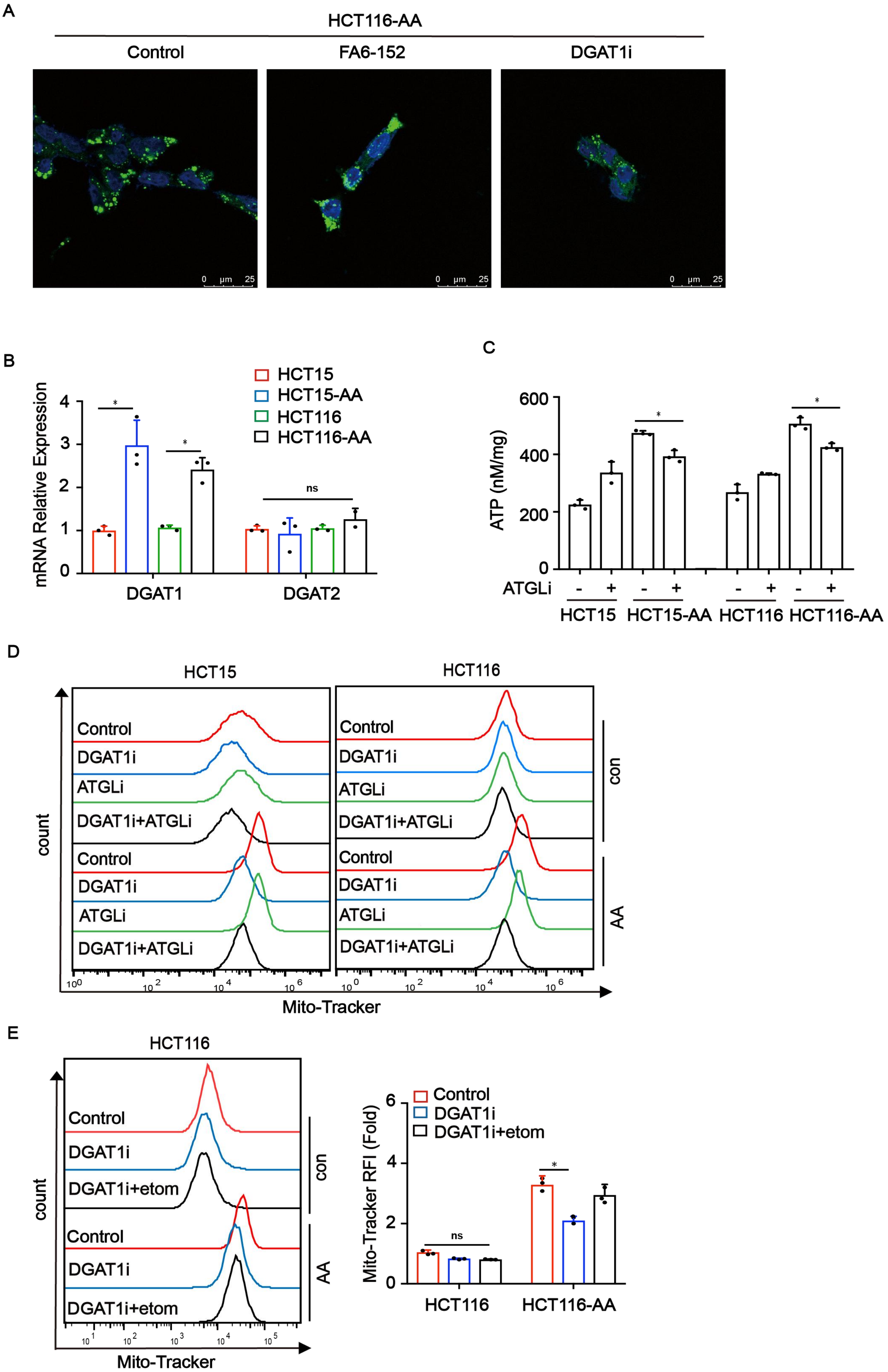

Supplementary Figure 4

A

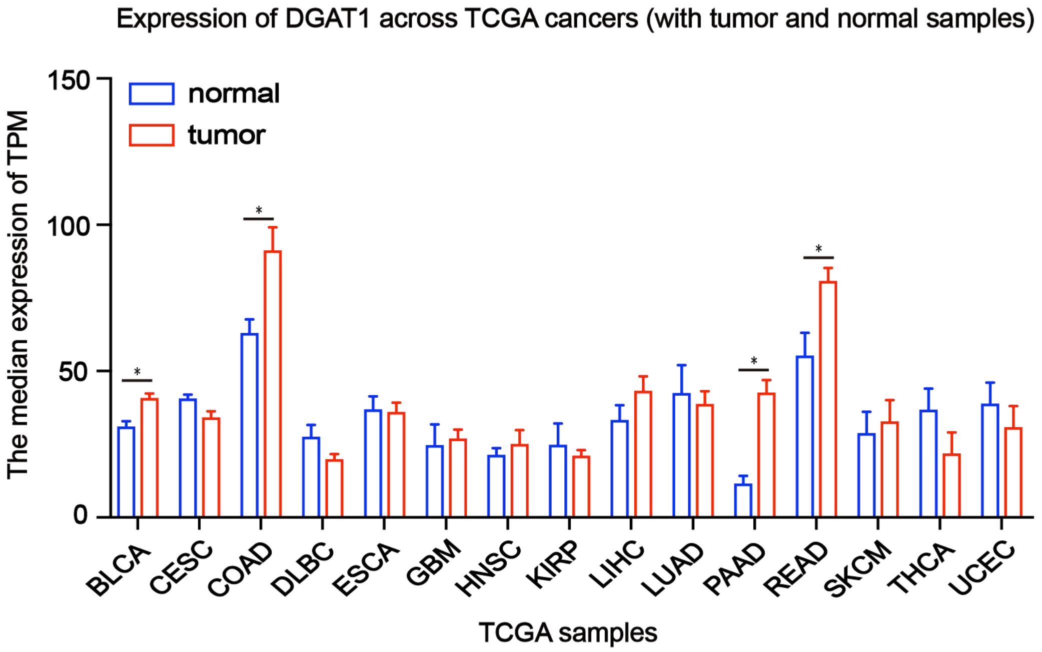

B

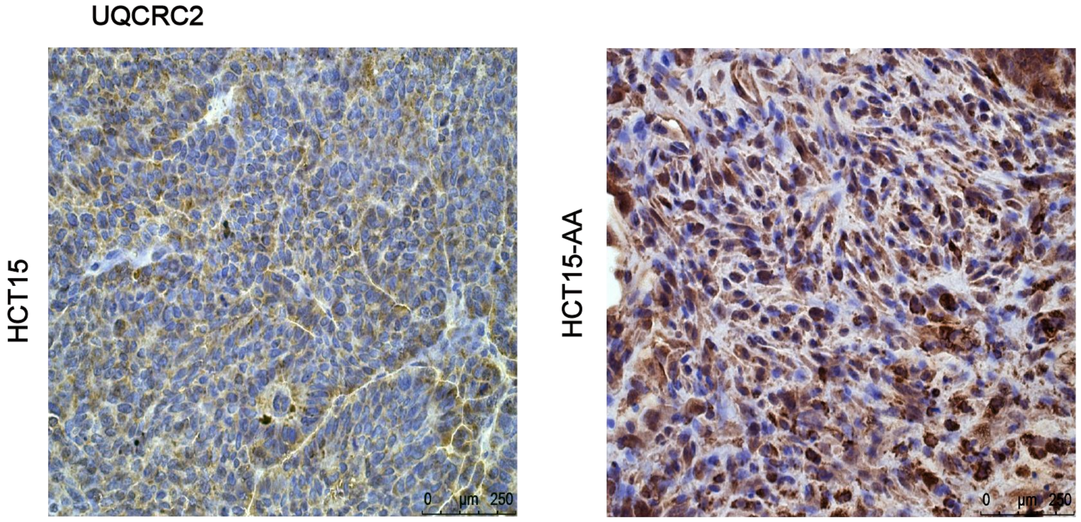

C

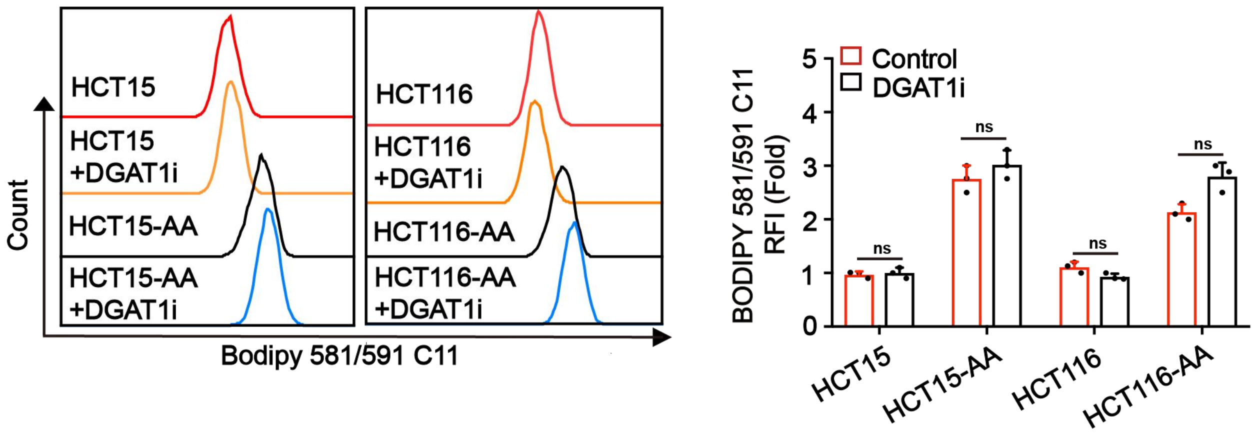

Supplement: Supplementary file 1 — Supplementary Figures [file 41420_2025_2301_MOESM1_ESM.pdf]
