# Supplementary material for: Autophagic flux-lipid droplet biogenesis cascade sustains mitochondrial fitness in colorectal cancer cells adapted to acidosis
Source: Cell Death Discov. 2025 Jan 25;11:21. doi: 10.1038/s41420-025-02301-6 (PMC11761495; doi:10.1038/s41420-025-02301-6)
Supplement: Supplementary file 2 — Supplementary Figure Legends [file 41420_2025_2301_MOESM2_ESM.docx]

**Supplementary Figure Legends**

**Supplementary Figure 1. Enrichment analysis of differentially expressed genes。**

1. Hallmark pathways enriched in control cells or acid-adapted cells by gene set enrichment analysis (GSEA). The complete lists of differentially expressed genes were shown in Supplementary Table 1.

**Supplementary Figure 2. mtROS participated in maintaining mitochondrial mass in colorectal cancer cells under acidic microenvironment.**

(A) Mitochondrial content was quantified by flow cytometry in CRC and CRC-AA cells treated with mitoQ (500 nM or 1 μM) from 24 h detected by Mito-tracker. (B) Apoptotic levels of CRC and CRC-AA cells after treatment with mitoQ (125 nM) from 12 h to 48 h. (C) Western blot analysis of LC3B, p62 protein level in CRC and CRC-AA cells treated with mitoQ (125 nM) from 24 h. GAPDH was used as a loading control. (D) Western blot analysis of p-AMPKα, AMPKα, PGC-1α protein level in CRC and CRC-AA cells. GAPDH was used as a loading control. (E) Western blot analysis of p-AMPKα, AMPKα protein level in CRC and CRC-AA cells treated with mitoQ (125 nM) from 24 h. GAPDH was used as a loading control. * *p*＜0.05, ns indicates *p* > 0.05.

**Supplementary Figure 3. Autophagy-dependent lipid droplets maintain mitochondrial mass and fitness in colorectal cancer cells under acidic microenvironment.**

(A) BODIPY staining was used to detect lipid droplets content in CRC-AA cells treated with DGAT1 inhibitor (A922500, 10 μM) or anti-CD36 antibody by immunofluorescence. Scale bar: 25 μm. (B) mRNA levels of DGAT1 and DGAT2 genes measured by qPCR. (C) Steady-state cellular ATP levels were measured using an ATP luciferase Kit in CRC and CRC-AA cells treated with or without ATGL inhibitor (10 μM). (D) Mitochondrial content was quantified by flow cytometry in CRC and CRC-AA cells treated with DGAT1 inhibitor or ATGL inhibitor for 24 h. (E) Mitochondrial content was quantified by flow cytometry in CRC and CRC-AA cells treated with DGAT1 inhibitor or etomoxir (20 μM) for 24 h. * *p*＜0.05, ***p*＜0.01, ns indicates *p* > 0.05.

**Supplementary Figure 4. Mitochondria supports tumor growth *in vivo***

(A) DGAT1 expression in a variety of human solid tumors in the TCGA database. COAD: colon adenocarcinoma, READ: rectal cancer. (B) Representative IHC images showing the UQCRC2 in HCT15 and HCT15-AA tumors. Scale bar: 250 μm. (C) BODIPY (581/591 C11) staining was used to detect lipid peroxidation in CRC and CRC-AA cells treated with DGAT1 inhibitor for 72 h by flow cytometry. * *p*＜0.05, ns indicates *p* > 0.05.
