# Supplementary material for: Autophagic flux-lipid droplet biogenesis cascade sustains mitochondrial fitness in colorectal cancer cells adapted to acidosis
Source: Cell Death Discov. 2025 Jan 25;11:21. doi: 10.1038/s41420-025-02301-6 (PMC11761495; doi:10.1038/s41420-025-02301-6)
Supplement: Supplementary file 3 — Supplementary Table 1, 2, 3 [file 41420_2025_2301_MOESM3_ESM.docx]

**Supplementary Table 1. List of changed genes in cells adapted to pH6.5 medium vs. control cells**

| Gene_ID | Gene_Name | read count  _control 01 | read count _control 02 | read count _control 03 | read count  _Adapted 01 | read count _Adapted 02 | read count  _Adapted 03 | FDR | log2  FoldChange |
| --- | --- | --- | --- | --- | --- | --- | --- | --- | --- |
| ENSG00000005001 | PRSS22 | 45.2064 | 36.4985 | 45.4745 | 106.237 | 65.819 | 206.016 | 0.04896712 | 1.581257031 |
| ENSG00000005189 | AC004381 .6 | 6.42488 | 5.35286 | 5.61371 | 11.6078 | 13.0259 | 11. 1679 | 7.56E-06 | 1.089870054 |
| ENSG00000005884 | ITGA3 | 60.9131 | 67.587 | 65.3925 | 210. 197 | 153.241 | 370.855 | 0.0032857 | 1.926793023 |
| ENSG00000006062 | MAP3K14 | 3. 14843 | 4.06814 | 3.73738 | 6.90861 | 8.0467 | 8.50596 | 5.08E-08 | 1. 126270424 |
| ENSG00000006210 | CX3CL1 | 6.21704 | 4.38854 | 3.68654 | 15.8082 | 14.501 | 18. 1346 | 1.78E-17 | 1.761230364 |
| ENSG00000006327 | TNFRSF1A | 127.524 | 90.6075 | 95.2949 | 492.517 | 238.445 | 416.084 | 2.35E-06 | 1.634276805 |
| ENSG00000008394 | MGST1 | 68.712 | 47.3041 | 42.0202 | 165.234 | 150.608 | 162.072 | 2.62E-17 | 1.612819957 |
| ENSG00000008405 | CRY1 | 8.9631 | 9.01461 | 7.63628 | 18.8481 | 21. 1862 | 20.8138 | 1.61E-12 | 1.388473342 |
| ENSG00000011426 | ANLN | 23.9687 | 16.0034 | 13.023 | 63. 1848 | 69.6877 | 63.2496 | 9.93E-26 | 1.967647379 |
| ENSG00000012061 | ERCC1 | 89.4159 | 75.5482 | 81.7636 | 280.872 | 201.869 | 307.097 | 1.81E-10 | 1.696973812 |
| ENSG00000013588 | GPRC5A | 8.22447 | 10.686 | 6.38289 | 60.26 | 41.4551 | 106.707 | 0.00462848 | 3.280017417 |
| ENSG00000019186 | CYP24A1 | 0.494706 | 0. 121146 | 0.21909 | 7.87192 | 4.82524 | 16.2985 | 0.00066068 | 5. 171044816 |
| ENSG00000023445 | BIRC3 | 4.56849 | 3.3873 | 2.66168 | 8.44436 | 9.08929 | 6.32836 | 4.76E-06 | 1. 104052288 |
| ENSG00000026103 | FAS | 9.98584 | 7.04669 | 5.97256 | 17.7954 | 19.5479 | 14.2736 | 2.89E-07 | 1. 106304468 |
| ENSG00000028137 | TNFRSF1B | 8.65612 | 8.69614 | 7.95934 | 21.8492 | 15.6552 | 31.4862 | 0.0019643 | 1.458557033 |
| ENSG00000029534 | ANK1 | 0.048542 | 0.0281204 | 0.0250728 | 0. 1391 | 0.366638 | 0. 192667 | 2.05E-05 | 2.778703435 |
| ENSG00000033170 | FUT8 | 11.8961 | 14.8942 | 13.7193 | 29.0157 | 30.0224 | 32.8082 | 1.75E-08 | 1.054876423 |
| ENSG00000033867 | SLC4A7 | 6.98732 | 9.50843 | 6.95598 | 24. 1223 | 24.8928 | 14.8556 | 1.08E-10 | 1.410040486 |
| ENSG00000034152 | MAP2K3 | 24.4473 | 28.7473 | 20.0537 | 50.3304 | 42.3885 | 69.8664 | 4.43E-05 | 1.362829343 |
| ENSG00000035664 | DAPK2 | 0.413281 | 0.514467 | 0.868198 | 1.21935 | 1. 15041 | 0.854407 | 0.02797927 | 1. 110632699 |
| ENSG00000035862 | TIMP2 | 43.3281 | 52.4723 | 54.6225 | 114.818 | 93.2094 | 136. 152 | 1.72E-05 | 1.208545955 |
| ENSG00000048740 | CELF2 | 1. 10663 | 1.4001 | 0.741404 | 6.63914 | 7. 12307 | 4.74206 | 4.36E-32 | 2.66200951 |
| ENSG00000049130 | KITLG | 9. 16512 | 4.33922 | 2.90573 | 43.6804 | 53.8137 | 19.241 | 0.00024714 | 2.789391188 |
| ENSG00000052802 | MSMO1 | 36.0875 | 27.3716 | 28.995 | 112.985 | 108.599 | 59.3579 | 1.38E-05 | 1.594267014 |
| ENSG00000053524 | MCF2L2 | 1.70768 | 1.83553 | 1.80078 | 3.50862 | 3.66431 | 4.54593 | 0.00094198 | 1. 10255851 |
| ENSG00000053747 | LAMA3 | 3.20566 | 2.78603 | 3. 13212 | 9.06739 | 7.92101 | 8.77047 | 1.48E-14 | 1.444156493 |
| ENSG00000054967 | RELT | 7.00263 | 4.62788 | 4.70181 | 10.2997 | 8.90584 | 13.8114 | 0.00026357 | 1.044513326 |
| ENSG00000055813 | CCDC85A | 0.07406 | 0.0603971 | 0.0360095 | 1. 19011 | 0.997246 | 0.566262 | 3.88E-08 | 4. 15324908 |
| ENSG00000115594 | IL1R1 | 1.40994 | 0.887955 | 0.809394 | 2.34967 | 2.25803 | 2.54076 | 0.00087402 | 1. 158556033 |
| ENSG00000057019 | DCBLD2 | 25.9142 | 16.9834 | 11.9869 | 64.3212 | 70.6435 | 61.6253 | 2.32E-06 | 1.589280406 |
| ENSG00000058091 | CDK14 | 0.370182 | 0. 110647 | 0.023168 | 0.243483 | 0.345787 | 0.324233 | 0.00024475 | 2. 12795578 |
| ENSG00000058335 | RASGRF1 | 0.979414 | 0.916938 | 0.729479 | 1.78233 | 1.20181 | 1.31758 | 0.01377795 | 1.003337592 |
| ENSG00000058668 | ATP2B4 | 2.30179 | 2.57239 | 2.49342 | 7.80333 | 7.38417 | 5.7834 | 1.72E-13 | 1.435780436 |
| ENSG00000059915 | PSD | 0.522591 | 0.299266 | 0.412952 | 1.5262 | 1.08473 | 2.32826 | 2.59E-05 | 1.948220498 |
| ENSG00000064042 | LIMCH1 | 2.88252 | 3.45334 | 3.7921 | 9.78287 | 9.99871 | 9.50381 | 4.24E-13 | 1.518986522 |
| ENSG00000064607 | SUGP2 | 16. 1479 | 18.0782 | 14.0505 | 34.7544 | 37. 1521 | 41. 1624 | 6.86E-11 | 1.217962223 |
| ENSG00000064787 | BCAS1 | 0.013994 | 0 | 0.0660304 | 0.438762 | 0.522766 | 0.638395 | 6.86E-11 | 4.392412901 |
| ENSG00000064932 | SBNO2 | 27. 1298 | 22. 1651 | 23.3249 | 50.8309 | 44.3143 | 74.3474 | 2.30E-07 | 1.206533779 |

| ENSG00000065618 | COL17A1 | 0.673658 | 0.739945 | 1.26854 | 3.51949 | 2.40333 | 6.98268 | 6.47E-08 | 1.753937256 |
| --- | --- | --- | --- | --- | --- | --- | --- | --- | --- |
| ENSG00000066248 | NGEF | 6.728 | 6. 10801 | 6.74979 | 22.2996 | 16. 1818 | 23.859 | 7.59E-11 | 1.716105388 |
| ENSG00000067064 | IDI1 | 23.9813 | 10.0829 | 9.5398 | 57.2667 | 62.9118 | 37.8057 | 4.71E-06 | 1.803551662 |
| ENSG00000269335 | IKBKG | 11.7609 | 15.0592 | 13.6756 | 12.9608 | 11.6607 | 20.2044 | 0. 12710175 | 0.725667447 |
| ENSG00000067798 | NAV3 | 0.595283 | 0. 173957 | 0.0507438 | 0.315624 | 0.623783 | 0. 150232 | 0.00716229 | 1.998435479 |
| ENSG00000069424 | KCNAB2 | 8.01185 | 10.9611 | 12.8437 | 24.4818 | 22.7942 | 19.2553 | 2.52E-06 | 1.022517285 |
| ENSG00000069696 | DRD4 | 0.431878 | 0. 163052 | 0.57598 | 2.62266 | 1. 18344 | 5.90538 | 0.02255597 | 3. 117030699 |
| ENSG00000070731 | ST6GALNAC2 | 5.38241 | 4.38416 | 4.8017 | 12.4986 | 11.5295 | 11.4022 | 4. 11E-07 | 1.254635789 |
| ENSG00000072786 | STK10 | 3.49151 | 3.31972 | 3.25733 | 7.25841 | 5.41344 | 10.4038 | 0.0023454 | 1. 102661194 |
| ENSG00000072952 | MRVI1 | 0. 100325 | 0. 106371 | 0.0707479 | 0.359264 | 0.915931 | 0.353803 | 1.48E-05 | 2.298876392 |
| ENSG00000072954 | TMEM38A | 6. 19134 | 6.3254 | 7.37986 | 18.7682 | 15.3767 | 21.9916 | 2.97E-11 | 1.455869072 |
| ENSG00000073146 | MOV10L1 | 0.02894 | 0. 100517 | 0.227864 | 0.87331 | 0.626047 | 2.0856 | 0.00966738 | 3.393299217 |
| ENSG00000073331 | ALPK1 | 0.422298 | 0.539475 | 0.536901 | 3.06037 | 2.95051 | 2.85271 | 1.21E-14 | 2.549410839 |
| ENSG00000073670 | ADAM11 | 0.938554 | 0.949002 | 1.37447 | 2.4808 | 1.96394 | 2.83166 | 0.00038255 | 1. 169246147 |
| ENSG00000074416 | MGLL | 3.4745 | 2.44126 | 2.29775 | 18.7515 | 12.8916 | 24.0848 | 5.79E-08 | 2.781316303 |
| ENSG00000074660 | SCARF1 | 0.300544 | 0.373296 | 0.265643 | 0.661989 | 0.475773 | 0.807032 | 0.02321663 | 1. 189996791 |
| ENSG00000075218 | GTSE1 | 16.755 | 13.6754 | 10.8298 | 29.0149 | 34.7518 | 26.8924 | 2.36E-07 | 1. 13825771 |
| ENSG00000075702 | WDR62 | 7.20196 | 4.09034 | 3.28322 | 15. 1 161 | 15.5344 | 22.6073 | 2.39E-07 | 1.857860132 |
| ENSG00000076382 | SPAG5 | 15.9159 | 10.6182 | 11.9437 | 31.7688 | 36.0407 | 52.5449 | 2.23E-17 | 1.552090722 |
| ENSG00000077152 | UBE2T | 41.2383 | 27.92 | 25.7238 | 66.9586 | 69.8772 | 91.3958 | 0.00013927 | 1.24854217 |
| ENSG00000077514 | POLD3 | 7.77814 | 7.71511 | 6.52243 | 17.5824 | 17.9444 | 14.8648 | 4.21E-11 | 1.271749837 |
| ENSG00000079337 | RAPGEF3 | 0.411481 | 0.482397 | 0.588105 | 2.37311 | 1.07377 | 2.69051 | 0.00155011 | 1.875389105 |
| ENSG00000079459 | FDFT1 | 122.494 | 71. 1907 | 70.2349 | 278.38 | 282.36 | 239.279 | 5.73E-15 | 1.594186583 |
| ENSG00000082497 | SERTAD4 | 0.244147 | 0.219246 | 0.209618 | 2.41091 | 2.20623 | 1.69417 | 2.34E-15 | 3.248142335 |
| ENSG00000082781 | ITGB5 | 30.8198 | 26. 1099 | 26.6658 | 91.741 | 74.3741 | 107.264 | 3.77E-07 | 1.746567186 |
| ENSG00000084710 | EFR3B | 0.514956 | 0.251564 | 0.353872 | 2.21122 | 2.33716 | 3. 1 1544 | 2.54E-16 | 2.848727319 |
| ENSG00000085999 | RAD54L | 13.7069 | 8.28342 | 6.46721 | 20.2242 | 22.0804 | 18.6601 | 0.00011541 | 1.051751244 |
| ENSG00000086015 | MAST2 | 22.9985 | 19.3241 | 20.8771 | 43.5715 | 36.5539 | 56.3052 | 1. 10E-06 | 1.097547767 |
| ENSG00000086061 | DNAJA1 | 28.9138 | 18.4304 | 18.5705 | 46.7135 | 46.744 | 47.3539 | 7.50E-07 | 1.049295002 |
| ENSG00000087586 | AURKA | 30.4542 | 23.5316 | 20.2651 | 74.5092 | 79.3833 | 85.7452 | 3.42E-10 | 1.687851715 |
| ENSG00000087589 | CASS4 | 0. 194838 | 0. 183167 | 0. 183736 | 0.728663 | 0.705026 | 1. 11094 | 2.75E-05 | 2.262828711 |
| ENSG00000088325 | TPX2 | 39.5948 | 29.6252 | 23.0828 | 66.8868 | 70.8858 | 66.3426 | 1.03E-08 | 1. 101687672 |
| ENSG00000088726 | TMEM40 | 1. 17887 | 0.244156 | 0.350322 | 10.57 | 7.72582 | 9.52121 | 4.92E-12 | 3.830261438 |
| ENSG00000089159 | PXN | 72.9706 | 72.6154 | 71.7702 | 143.935 | 117.035 | 206.387 | 0.00242799 | 1. 159398066 |
| ENSG00000090975 | PITPNM2 | 4.43889 | 3.6898 | 3.91036 | 8. 10182 | 6.94236 | 10.5022 | 3.63E-07 | 1. 187832949 |
| ENSG00000091137 | SLC26A4 | 0.207563 | 0.232274 | 0.221043 | 0.576823 | 0.717463 | 0.355852 | 0.01284283 | 1.296600091 |
| ENSG00000091622 | PITPNM3 | 3. 16644 | 3.4788 | 3.46475 | 8.36896 | 7.46251 | 8.38359 | 7.82E-11 | 1.268400241 |
| ENSG00000091651 | ORC6 | 20. 1671 | 13.0419 | 12.0367 | 35.8444 | 35.9407 | 40.7083 | 4.64E-09 | 1.295159993 |
| ENSG00000092140 | G2E3 | 6.68716 | 7.02477 | 5.81491 | 13.0993 | 14.7824 | 12.2326 | 7.91E-08 | 1.052370357 |
| ENSG00000092295 | TGM1 | 1.5745 | 1.38257 | 0.781731 | 1.98949 | 3.35903 | 5.45991 | 0.02152234 | 1.657602748 |
| ENSG00000092470 | WDR76 | 10. 1816 | 7.41755 | 5.86218 | 17.9181 | 18.5298 | 12.62 | 1.77E-05 | 1. 141490925 |
| ENSG00000092820 | EZR | 117.014 | 128.6 | 127.536 | 305.803 | 238.385 | 495.372 | 0.00529185 | 1.452652668 |
| ENSG00000096968 | JAK2 | 0.881254 | 1.08693 | 0.840074 | 2.03853 | 2.24067 | 1.81371 | 0.00190592 | 1.097775337 |

| ENSG00000099194 | SCD | 80.0683 | 56.9956 | 56.6713 | 232.047 | 234.042 | 122.379 | 0.00028983 | 1.585891135 |
| --- | --- | --- | --- | --- | --- | --- | --- | --- | --- |
| ENSG00000099377 | HSD3B7 | 47. 139 | 29. 1654 | 31.6624 | 78.4464 | 81.4915 | 80.8141 | 7.20E-10 | 1. 14298846 |
| ENSG00000099994 | SUSD2 | 0.414811 | 0.741366 | 1.00814 | 3.67411 | 2.60033 | 4.64203 | 1.01E-08 | 2.34082794 |
| ENSG00000100003 | SEC14L2 | 5.51648 | 7.53971 | 5.72178 | 11.3552 | 9.25099 | 15.9163 | 0.00030393 | 1.419205215 |
| ENSG00000100055 | CYTH4 | 0.357699 | 0. 166067 | 0. 196286 | 0.695799 | 0.768152 | 1.0082 | 1.97E-05 | 2. 188339121 |
| ENSG00000100092 | SH3BP1 | 13.4336 | 9.91237 | 9.91151 | 22.6286 | 22.2716 | 30.0327 | 1.81E-10 | 1. 195354249 |
| ENSG00000100234 | TIMP3 | 0.064934 | 0.239386 | 0.497879 | 3.26337 | 3.68211 | 4.68375 | 9.28E-18 | 3.907706019 |
| ENSG00000100342 | APOL1 | 0.695422 | 0.409384 | 0.782698 | 5.06801 | 5.77244 | 6.72005 | 7.72E-21 | 3. 188931312 |
| ENSG00000100490 | CDKL1 | 1.00484 | 1. 17344 | 1.23981 | 1.96368 | 2.65817 | 2.29882 | 0.03271926 | 1.00604252 |
| ENSG00000100504 | PYGL | 10.5515 | 8.94664 | 8. 10027 | 19. 1639 | 16.85 | 23.601 | 1.59E-06 | 1.05281896 |
| ENSG00000100711 | ZFYVE21 | 26. 1967 | 20. 129 | 21.723 | 49.315 | 42.0926 | 54.0425 | 8.88E-09 | 1. 11795552 |
| ENSG00000100739 | BDKRB1 | 0.376726 | 1.02732 | 0.93751 | 3.69419 | 3. 13647 | 6.50303 | 0.00029167 | 2.432807151 |
| ENSG00000100749 | VRK1 | 13. 1519 | 10.4955 | 9.62832 | 20. 1009 | 21.4826 | 28.9456 | 0.00023761 | 1.017484472 |
| ENSG00000101003 | GINS1 | 15.769 | 10.8064 | 11.7518 | 31.9619 | 32.4445 | 24.4923 | 1.87E-10 | 1.20067531 |
| ENSG00000101187 | SLCO4A1 | 34.0423 | 28.3287 | 32.7571 | 132.079 | 89.351 | 257.092 | 0.00142955 | 2.371392761 |
| ENSG00000101670 | LIPG | 2.43579 | 0.538763 | 0.91268 | 5.85897 | 6.44791 | 3.41134 | 1.91E-11 | 2.584314175 |
| ENSG00000101871 | MID1 | 11.2412 | 10.2519 | 6.94112 | 31.7184 | 37.0905 | 14.4647 | 3.54E-06 | 1.398777744 |
| ENSG00000102057 | KCND1 | 0.34522 | 0.381243 | 0.56749 | 1.57567 | 1.39151 | 2.75759 | 0.02654274 | 2.056284296 |
| ENSG00000102243 | VGLL1 | 0.343932 | 0 | 0.049688 | 7.34509 | 4.01762 | 9.99524 | 3.54E-23 | 7.995836763 |
| ENSG00000102362 | SYTL4 | 1.63755 | 1.97323 | 1.82881 | 4.99884 | 4.99483 | 5.94851 | 1.73E-08 | 1.516403232 |
| ENSG00000102780 | DGKH | 2.64456 | 2.42429 | 1.78005 | 4.23266 | 4.27161 | 3.73884 | 1.00E-07 | 1.076391417 |
| ENSG00000102854 | MSLN | 1.0802 | 1.48757 | 1.97273 | 5.81421 | 3.45241 | 6.22296 | 5.45E-08 | 1.965228637 |
| ENSG00000102962 | CCL22 | 0 | 0.0780436 | 0 | 0.655088 | 0.438754 | 1. 13908 | 0.00020144 | 4.997982791 |
| ENSG00000103056 | SMPD3 | 0.550038 | 0.332072 | 0.607255 | 0.753667 | 0.713774 | 1.03482 | 0.00960985 | 1. 151047828 |
| ENSG00000103121 | CMC2 | 38.0394 | 23.4814 | 28.0184 | 63.8716 | 55.7237 | 86.8993 | 0.00032583 | 1.00048189 |
| ENSG00000103241 | FOXF1 | 0.326516 | 0.208874 | 0.357031 | 1. 18982 | 1. 10248 | 1.6754 | 3.57E-06 | 2.229279048 |
| ENSG00000103647 | CORO2B | 1.04189 | 1. 10471 | 0.961148 | 2.29841 | 1.83984 | 3.61603 | 0.00483504 | 1.314204604 |
| ENSG00000103995 | CEP152 | 3.49979 | 3.89859 | 2.88478 | 8.88355 | 9.25062 | 9. 13096 | 8. 15E-12 | 1.338531708 |
| ENSG00000104267 | CA2 | 9.8891 | 7.46031 | 8.01096 | 39.2347 | 35.9548 | 41.287 | 5.93E-28 | 2. 195495156 |
| ENSG00000104368 | PLAT | 6.75138 | 4.616 | 4.28683 | 63.5147 | 45.0998 | 151.636 | 0.00273869 | 4. 105921462 |
| ENSG00000104375 | STK3 | 6.32291 | 7.59673 | 5.81579 | 15.2697 | 13.4231 | 13. 181 1 | 5.42E-07 | 1. 102374858 |
| ENSG00000104450 | SPAG1 | 3. 1931 | 3.3002 | 2.8708 | 6.2067 | 7.23123 | 5.4998 | 1.61E-05 | 1. 1 13340588 |
| ENSG00000104549 | SQLE | 26.3936 | 20.0604 | 17.4787 | 61.2906 | 68.2151 | 44.6844 | 3.39E-05 | 1.39807388 |
| ENSG00000104783 | KCNN4 | 1. 10366 | 1.54175 | 1.27287 | 30.2088 | 17. 1 145 | 54.0888 | 2.65E-05 | 4.727017206 |
| ENSG00000104881 | PPP1R13L | 56.0122 | 58.7699 | 56.3884 | 110.218 | 74.7442 | 171.71 | 0.00218119 | 1. 10874017 |
| ENSG00000104894 | CD37 | 0.704322 | 1.25629 | 0.542131 | 1.47557 | 2. 12253 | 1.53082 | 0.0162306 | 1.251514522 |
| ENSG00000104901 | DKKL1 | 1. 161 | 1.21393 | 1.63562 | 2.31138 | 2.8155 | 4.50197 | 0.03143057 | 1. 168153288 |
| ENSG00000104951 | IL4I1 | 1.48937 | 0.735948 | 0.999497 | 1.86585 | 1.51692 | 2.70045 | 0.0114353 | 1. 193555502 |
| ENSG00000105011 | ASF1B | 45.7518 | 28.2891 | 28.262 | 63.6723 | 69.6171 | 64.7834 | 1.59E-07 | 1.008963439 |
| ENSG00000105339 | DENND3 | 13.0145 | 14.5758 | 14.8083 | 28.5614 | 23.2113 | 39. 1493 | 0.00019882 | 1. 169446196 |
| ENSG00000105426 | PTPRS | 0.961555 | 0.866162 | 0.826367 | 6.69509 | 5.00798 | 13.0547 | 2.25E-05 | 3.222822927 |
| ENSG00000105767 | CADM4 | 6.66642 | 8.54862 | 10.2484 | 24.8335 | 24.3826 | 33.3637 | 3.65E-13 | 1.716573322 |
| ENSG00000105771 | SMG9 | 16.8452 | 18.5654 | 15.6509 | 36.7626 | 35.0764 | 37.9664 | 1.41E-10 | 1.216322781 |

| ENSG00000105808 | RASA4 | 1.97752 | 1.38403 | 2. 17878 | 5.61973 | 5.04192 | 7.79219 | 1.52E-12 | 1.969594946 |
| --- | --- | --- | --- | --- | --- | --- | --- | --- | --- |
| ENSG00000105835 | NAMPT | 45.9017 | 47.7831 | 35.579 | 99.3684 | 83.266 | 102. 101 | 0.00187391 | 1. 128086262 |
| ENSG00000105971 | CAV2 | 10.5139 | 9.01259 | 8.98465 | 27.6601 | 24.0731 | 29.0555 | 8.77E-10 | 1.582872677 |
| ENSG00000105974 | CAV1 | 6.37042 | 4.85394 | 5.22645 | 30.3118 | 29.683 | 27.2622 | 5.47E-33 | 2.385511695 |
| ENSG00000105976 | MET | 9.22759 | 8.83172 | 7.6128 | 37.3234 | 33.2486 | 38.3811 | 2.89E-28 | 2.090249992 |
| ENSG00000106351 | AGFG2 | 11.5818 | 13. 1747 | 10.7746 | 23.8291 | 21.7885 | 28.5261 | 4.21E-11 | 1.252043087 |
| ENSG00000107738 | C10orf54 | 0.938583 | 2.31697 | 3.89165 | 10.726 | 8. 15577 | 13.291 | 4.20E-06 | 2.452623591 |
| ENSG00000107796 | ACTA2 | 7.64726 | 4.23652 | 4.55918 | 13.4587 | 12.2328 | 11.9879 | 6.54E-06 | 1.235087168 |
| ENSG00000107984 | DKK1 | 12. 1929 | 6.36386 | 5.34662 | 436.426 | 373. 169 | 453.721 | 2.59E-76 | 5.724493807 |
| ENSG00000108106 | UBE2S | 154.679 | 95.8968 | 120.221 | 249.619 | 217.946 | 393.293 | 0.00057708 | 1.269514883 |
| ENSG00000108602 | ALDH3A1 | 68.4986 | 50.7689 | 54.3462 | 233.364 | 216. 146 | 255.788 | 9.52E-25 | 1.999630426 |
| ENSG00000108622 | ICAM2 | 6.81148 | 4.73235 | 5.2875 | 11.8441 | 9. 13595 | 12.9474 | 0.00338708 | 1.017840389 |
| ENSG00000104365 | IKBKB | 16.9107 | 12.2901 | 15. 1518 | 27.5713 | 25.9995 | 34.9531 | 1.35E-05 | 0.906479791 |
| ENSG00000108960 | MMD | 8.70071 | 8.50733 | 7.48988 | 18.5661 | 15.7528 | 16.8525 | 3.91E-07 | 1. 124806607 |
| ENSG00000109107 | ALDOC | 10.4921 | 4. 15019 | 8.43653 | 19.9896 | 13.5603 | 20.2552 | 0.00271129 | 1.232183747 |
| ENSG00000109944 | C11orf63 | 0.650723 | 0.608972 | 0.919467 | 1.38816 | 1.58885 | 1.61062 | 0.00476139 | 1.268165279 |
| ENSG00000110080 | ST3GAL4 | 9.72849 | 6.97829 | 8.20911 | 18.8953 | 17.9377 | 21.6133 | 5.77E-09 | 1.285300107 |
| ENSG00000110092 | CCND1 | 111.496 | 107.411 | 101. 165 | 246.317 | 236.445 | 240.723 | 1.37E-07 | 1.200207023 |
| ENSG00000110427 | KIAA1549L | 0.477326 | 0.248647 | 0.271774 | 2. 14041 | 2.30447 | 1.90964 | 2.92E-22 | 2.908356346 |
| ENSG00000110888 | CAPRIN2 | 7.72211 | 7.29869 | 7.66103 | 20.0534 | 15.6888 | 23.5882 | 0.00055285 | 1.371712951 |
| ENSG0000011 1 145 | ELK3 | 10.206 | 8.61288 | 8.57657 | 22.4614 | 19.5684 | 25. 1463 | 2.87E-08 | 1.428251268 |
| ENSG00000111319 | SCNN1A | 72.3414 | 75. 1503 | 85.5672 | 180.858 | 155. 198 | 169.268 | 5.79E-08 | 1. 134393575 |
| ENSG0000011 1664 | GNB3 | 3.06707 | 2.39246 | 3.30594 | 4.57124 | 5.08496 | 8.23802 | 0.01857377 | 1.024448366 |
| ENSG00000173039 | RELA | 30.9897 | 34.0729 | 34. 1272 | 40.6671 | 37.7249 | 42.0578 | 0. 13183614 | 0.313108661 |
| ENSG0000011 1665 | CDCA3 | 23.3127 | 15.3427 | 13.6192 | 40.2099 | 39.2594 | 45.2892 | 1.92E-10 | 1.284641856 |
| ENSG00000112033 | PPARD | 10.4442 | 9.48439 | 10. 1196 | 23.6772 | 17.4004 | 32.7855 | 0.00062992 | 1.299919638 |
| ENSG00000112139 | MDGA1 | 0.481102 | 0.235222 | 0.254054 | 1. 18825 | 0.949307 | 0.871207 | 2.58E-11 | 2.423275565 |
| ENSG00000112183 | RBM24 | 3.35117 | 4.07042 | 2.7051 | 10.5297 | 11.2599 | 13.279 | 2. 10E-12 | 1.747874409 |
| ENSG00000112559 | MDFI | 4.03361 | 3.20409 | 4.80031 | 11.8105 | 6.55131 | 15.0751 | 0.01068825 | 1.451353095 |
| ENSG00000112599 | GUCA1B | 1.52778 | 2.81494 | 2.92966 | 6.39629 | 3.86596 | 7.93693 | 0.0083162 | 1.336225985 |
| ENSG00000112742 | TTK | 10.3469 | 8.36979 | 7.08599 | 18.6488 | 18.5444 | 21.4212 | 1.36E-05 | 1. 15067033 |
| ENSG00000112773 | FAM46A | 0.543196 | 0.605975 | 0.573791 | 2.63145 | 2. 11609 | 1.68677 | 1.49E-07 | 1.882656271 |
| ENSG00000112972 | HMGCS1 | 21.5415 | 14.7825 | 12.2622 | 44.215 | 52.2417 | 27.351 | 0.00069395 | 1.287497015 |
| ENSG00000112984 | KIF20A | 16.565 | 9.62937 | 7.45581 | 25.7221 | 27.9606 | 28.9932 | 5.92E-07 | 1.263716948 |
| ENSG00000113296 | THBS4 | 0. 18533 | 0. 142609 | 0. 160427 | 0.443271 | 0.740223 | 0.639694 | 0.00031717 | 1.946554453 |
| ENSG00000113810 | SMC4 | 17.3821 | 12.5097 | 7.29147 | 30.9442 | 32.8394 | 24.3289 | 1.21E-06 | 1.07409615 |
| ENSG00000115221 | ITGB6 | 0.769481 | 0.715879 | 0.336474 | 1.73717 | 1.84382 | 1.38621 | 0.0001936 | 1.505122028 |
| ENSG00000115363 | EVA1A | 3.58312 | 2.54239 | 3.85361 | 8.84018 | 6.52598 | 11.8737 | 0.01656723 | 1.55007121 |
| ENSG00000100324 | TAB1 | 14.0276 | 15.9704 | 16.3609 | 15.0442 | 15.5544 | 17.0893 | 0.04623081 | 0.863630282 |
| ENSG00000115520 | COQ10B | 12.8333 | 12.2759 | 10.958 | 22.5945 | 19.9387 | 28.5171 | 0.00384664 | 1.025383948 |
| ENSG00000115525 | ST3GAL5 | 4. 10546 | 2.86463 | 3.51887 | 9.61643 | 6.0331 | 6.0558 | 0.00644265 | 1.029098255 |
| ENSG00000115541 | HSPE1 | 265.948 | 109.33 | 117.315 | 289.725 | 286.028 | 408.82 | 0.02144899 | 1.012295741 |
| ENSG00000115594 | IL1R1 | 1.40994 | 0.887955 | 0.809394 | 2.34967 | 2.25803 | 2.54076 | 0.00087402 | 1. 158556033 |

| ENSG00000115641 | FHL2 | 43.6031 | 50.9271 | 43. 1431 | 92.6494 | 74.6573 | 142.34 | 0.01629 | 1. 1 18641341 |
| --- | --- | --- | --- | --- | --- | --- | --- | --- | --- |
| ENSG00000115648 | MLPH | 5.73864 | 5.38546 | 8.33357 | 24.022 | 14.3738 | 36. 1407 | 0.00148227 | 2.048561477 |
| ENSG00000115963 | RND3 | 3.57204 | 2.20195 | 2.56686 | 14. 1957 | 12.2537 | 16.0982 | 2.51E-11 | 2.334193991 |
| ENSG00000116191 | RALGPS2 | 6. 10316 | 7.42489 | 6.89842 | 16. 1224 | 14.8197 | 11.8718 | 2.28E-08 | 1.057945337 |
| ENSG00000184216 | IRAK1 | 88.8278 | 64.891 | 73.8696 | 98.2259 | 100.941 | 108.372 | 0.03038035 | 0.475609999 |
| ENSG00000116396 | KCNC4 | 2.34455 | 2.25081 | 2.29331 | 3. 13341 | 3.29282 | 2.67453 | 3.42E-05 | 1.072878728 |
| ENSG00000116871 | MAP7D1 | 32.775 | 25.4246 | 26.2318 | 54.3719 | 50.6396 | 76.5819 | 2.20E-08 | 1.060363937 |
| ENSG00000117115 | PADI2 | 0.523076 | 0.441616 | 0.842809 | 2.0784 | 1.29272 | 2.9589 | 0.00380468 | 1.898303283 |
| ENSG00000117226 | GBP3 | 5.54825 | 2.62127 | 1.62575 | 15.689 | 12.6494 | 11.3434 | 2.02E-23 | 2.297061662 |
| ENSG00000117399 | CDC20 | 105.456 | 87.5278 | 66.3777 | 158. 1 18 | 157.227 | 243.291 | 0.00259258 | 1.085051142 |
| ENSG00000117407 | ARTN | 5.50976 | 5.0872 | 6.46822 | 17.9306 | 13. 1159 | 38.614 | 0.01433445 | 1.947125238 |
| ENSG00000117525 | F3 | 4.22437 | 4.84044 | 5.59294 | 37.3863 | 26.2302 | 76.7776 | 0.00870902 | 3.299058447 |
| ENSG00000117650 | NEK2 | 17.2341 | 15.3119 | 12.3459 | 33.8239 | 33.9049 | 39. 1467 | 2.68E-08 | 1.226300352 |
| ENSG00000117877 | CD3EAP | 4.35262 | 4.27548 | 3.84947 | 10.864 | 8.97463 | 12.8965 | 4.25E-08 | 1.295365857 |
| ENSG00000118193 | KIF14 | 3.8688 | 2.57151 | 2.40431 | 7.36315 | 7.71205 | 7.36876 | 1.39E-10 | 1.273830633 |
| ENSG00000118276 | B4GALT6 | 2.88388 | 2.03971 | 2.32879 | 11. 1243 | 11.3528 | 8. 16795 | 9.27E-17 | 2.03678582 |
| ENSG00000118515 | SGK1 | 2.90301 | 1.28878 | 1.43793 | 5.78317 | 4.67418 | 3.67367 | 0.00041426 | 1.309724798 |
| ENSG00000118777 | ABCG2 | 1.56491 | 1.22293 | 1.20748 | 5.60906 | 4.91097 | 1.24768 | 0.04188301 | 1.273982833 |
| ENSG00000119125 | GDA | 15.9615 | 14.9906 | 7.68829 | 40.5333 | 42.2315 | 42.0397 | 4.72E-14 | 1.735217616 |
| ENSG00000119326 | CTNNAL1 | 57. 1544 | 42.8751 | 47.5939 | 116.904 | 91.9345 | 154.655 | 3.71E-10 | 1. 18390556 |
| ENSG00000119403 | PHF19 | 49.2164 | 35.472 | 41.08 | 64.9805 | 65.4009 | 75. 1821 | 7.03E-09 | 1.082945505 |
| ENSG00000119640 | ACYP1 | 17.9519 | 11.8028 | 10.075 | 45.4415 | 34.9927 | 58.2145 | 2.93E-05 | 1.811446245 |
| ENSG00000119714 | GPR68 | 0.534347 | 0.536212 | 0.744301 | 1.05439 | 1.27833 | 1.81344 | 0.03384256 | 1.053889094 |
| ENSG00000119917 | IFIT3 | 0.958404 | 0.770726 | 0.542981 | 2.7186 | 1.83094 | 2.06883 | 0.00037755 | 1.509550423 |
| ENSG00000120217 | CD274 | 0.496135 | 0.319057 | 0.294548 | 1.34071 | 0.812546 | 1.41966 | 0.00124365 | 1.660556688 |
| ENSG00000120318 | ARAP3 | 1.79748 | 1.74948 | 1.75577 | 7.04792 | 6.98364 | 9.3268 | 4.59E-21 | 2. 151874309 |
| ENSG00000120437 | ACAT2 | 16.4819 | 7.33421 | 6.69109 | 24.6858 | 30.4681 | 22.8622 | 0.00018507 | 1.339396134 |
| ENSG00000120526 | NUDCD1 | 10.0524 | 10. 1661 | 9.78447 | 19.4907 | 18.0422 | 25.5334 | 0.00229366 | 1.038432737 |
| ENSG00000120647 | CCDC77 | 3.64194 | 2.96629 | 2.59045 | 7.04599 | 7. 11717 | 7.8479 | 1.38E-05 | 1.258890255 |
| ENSG00000120694 | HSPH1 | 22.6424 | 14.7266 | 12.401 | 35.7377 | 36.8125 | 46.415 | 5.48E-07 | 1.233151012 |
| ENSG00000120875 | DUSP4 | 2.53459 | 2.90919 | 3.05927 | 8.3418 | 5.91087 | 12.7512 | 0.00683138 | 1.695739387 |
| ENSG00000120885 | CLU | 35.2298 | 40.5767 | 33.3144 | 97.3377 | 87.4073 | 111.502 | 2.68E-15 | 1.462679344 |
| ENSG00000120937 | NPPB | 0 | 0 | 0. 194188 | 5.65738 | 3.75312 | 43.9024 | 0.03228267 | 8.904188409 |
| ENSG00000121797 | CCRL2 | 0.38476 | 0.651572 | 0.492571 | 0.818509 | 0.836882 | 1.02358 | 0.04332916 | 1. 168069149 |
| ENSG00000122786 | CALD1 | 0.903258 | 0.730475 | 1. 19939 | 1.46657 | 1.85091 | 1.02732 | 0.01789354 | 1.003127244 |
| ENSG00000122861 | PLAU | 22.3512 | 11.5258 | 10.3385 | 61.5616 | 42.9385 | 77.5948 | 1.96E-05 | 2.038827617 |
| ENSG00000122966 | CIT | 8.89974 | 6.64919 | 5.21939 | 17.6066 | 17.2428 | 17.029 | 1. 10E-12 | 1.314897542 |
| ENSG00000123219 | CENPK | 13.6224 | 11.8832 | 12.767 | 31. 1033 | 37.552 | 35.523 | 3.25E-11 | 1.397578334 |
| ENSG00000123374 | CDK2 | 27.2371 | 19.6414 | 17.2479 | 55.6542 | 54.3109 | 48.2383 | 1.41E-12 | 1.314986574 |
| ENSG00000123485 | HJURP | 21.86 | 15.2895 | 11.67 | 49.4841 | 43.6998 | 64.6392 | 5.27E-06 | 1.680761639 |
| ENSG00000123843 | C4BPB | 4.97607 | 8. 14167 | 6.62947 | 11.235 | 11.2583 | 19.0769 | 0.03762906 | 1.071433912 |
| ENSG00000123975 | CKS2 | 207.574 | 117.942 | 124.033 | 431. 182 | 401.407 | 466.493 | 2.80E-12 | 1.512801286 |
| ENSG00000123983 | ACSL3 | 14.6254 | 15.0721 | 12.0878 | 29.327 | 32.0485 | 21.4487 | 3.35E-08 | 1.03980487 |

| ENSG00000125148 | MT2A | 886.321 | 373.758 | 349.728 | 4787.42 | 2872.27 | 8397. 16 | 0.00024549 | 3.377665797 |
| --- | --- | --- | --- | --- | --- | --- | --- | --- | --- |
| ENSG00000125885 | MCM8 | 6.01171 | 4.74878 | 3.86711 | 12.3253 | 13.5744 | 11.2763 | 2.78E-10 | 1.234843455 |
| ENSG00000126010 | GRPR | 0.039107 | 0.0420292 | 0.067393 | 2. 18256 | 1.54166 | 3.60091 | 7.43E-07 | 5.985205924 |
| ENSG00000126453 | BCL2L12 | 99.9689 | 58.8517 | 67.7996 | 146.758 | 117.48 | 200.499 | 0.00206627 | 1.013083221 |
| ENSG00000126778 | SIX1 | 0.446377 | 0.617441 | 0.399403 | 1.09437 | 1.4167 | 1.01027 | 0.01322305 | 1. 166234683 |
| ENSG00000126787 | DLGAP5 | 6.31743 | 5.68324 | 4.64711 | 14. 134 | 16.7183 | 11.7199 | 5.63E-10 | 1.28022474 |
| ENSG00000127129 | EDN2 | 0.501905 | 0. 168529 | 0.42495 | 1.33344 | 1.66499 | 4.03463 | 0.01616938 | 2.764616786 |
| ENSG00000127586 | CHTF18 | 34.979 | 26.0853 | 22.2091 | 51.8644 | 49.247 | 84. 1338 | 0.00060539 | 1. 182826609 |
| ENSG00000128271 | ADORA2A | 0.496625 | 0.660065 | 0.279621 | 2.23386 | 1.67319 | 4.30953 | 0.00025697 | 2.718908151 |
| ENSG00000128274 | A4GALT | 3.87027 | 3.93105 | 4.81709 | 9.09553 | 5.90171 | 12.4795 | 0.01134538 | 1. 131587951 |
| ENSG00000128284 | APOL3 | 0.217495 | 0. 105175 | 0. 161312 | 1.21054 | 1.38468 | 1.58533 | 2.23E-09 | 3.211205372 |
| ENSG00000128342 | LIF | 20.6544 | 8.94758 | 10.401 | 32. 1 183 | 24.2832 | 49.8701 | 0.02476993 | 1.506775295 |
| ENSG00000128482 | RNF112 | 0.22455 | 0.62177 | 0. 115596 | 0.716487 | 0.637827 | 3. 16252 | 0.00149574 | 2.517379699 |
| ENSG00000128510 | CPA4 | 0.807138 | 0.280544 | 0.410858 | 16.3096 | 12. 1618 | 25.4216 | 2.06E-11 | 5.94412294 |
| ENSG00000128567 | PODXL | 15.774 | 22.0298 | 16.9713 | 51.8581 | 46.6119 | 35.5444 | 7.55E-05 | 1.282571818 |
| ENSG00000128578 | STRIP2 | 1.68399 | 1.41945 | 1.09143 | 3.43537 | 3.45897 | 3.62965 | 7.95E-06 | 1.336045939 |
| ENSG00000128944 | KNSTRN | 34.9895 | 30.2584 | 24.6838 | 65.4956 | 65.2951 | 88.6554 | 1.08E-05 | 1.250434113 |
| ENSG00000129226 | CD68 | 4.26551 | 5.86229 | 5.08771 | 46.2595 | 31.4555 | 54.6509 | 5.03E-12 | 3. 131484607 |
| ENSG00000129355 | CDKN2D | 6.96281 | 6.26464 | 6.83113 | 17. 1417 | 15.3799 | 17.4939 | 8.34E-07 | 1.336391441 |
| ENSG00000129675 | ARHGEF6 | 0.220009 | 0. 15311 1 | 0.245712 | 2.44712 | 2.2278 | 1.69989 | 7. 16E-17 | 3.408244426 |
| ENSG00000130164 | LDLR | 20.7981 | 8.79983 | 8.60889 | 58.613 | 61.3152 | 45.5441 | 6.56E-15 | 2.097781985 |
| ENSG00000130589 | HELZ2 | 9.29165 | 9.8956 | 8. 1 1805 | 17.2247 | 16.4644 | 23. 1197 | 7.03E-09 | 1.081477035 |
| ENSG00000130695 | CEP85 | 11.7435 | 9. 1478 | 8.27766 | 21.6306 | 18.2012 | 23.2845 | 8.41E-09 | 1.094263167 |
| ENSG00000130702 | LAMA5 | 31.4863 | 35. 1241 | 34.0647 | 60.9909 | 48.7855 | 116.467 | 0.04681419 | 1. 164333299 |
| ENSG00000131016 | AKAP12 | 7.02132 | 5.32506 | 2.95828 | 40.4019 | 34.2969 | 52.7779 | 2.03E-13 | 2.99891567 |
| ENSG00000131061 | ZNF341 | 3.45572 | 2.94182 | 3.49006 | 6.88766 | 5.35193 | 9.92548 | 0.00266667 | 1. 155318952 |
| ENSG00000131069 | ACSS2 | 19.7667 | 16.9409 | 17.6171 | 41.6516 | 37.2018 | 35.0925 | 9. 14E-09 | 1.073653181 |
| ENSG00000131094 | C1QL1 | 4.58478 | 1.91868 | 3.2511 | 7.21315 | 5.01912 | 7.3714 | 0.00555409 | 1.068948027 |
| ENSG00000131459 | GFPT2 | 0.676143 | 0.343421 | 0.81268 | 1.04157 | 1.29091 | 1.59082 | 0.00713689 | 1.276990953 |
| ENSG00000131462 | TUBG1 | 38.9949 | 22.6413 | 26.0516 | 58.8337 | 51.8193 | 80.7953 | 0.00050196 | 1. 120270645 |
| ENSG00000131480 | AOC2 | 0.863046 | 0.725321 | 1. 19566 | 2. 19925 | 1.55337 | 3. 18909 | 0.01184123 | 1.344072907 |
| ENSG00000131620 | ANO1 | 0.780531 | 0.610194 | 0.817902 | 10.8704 | 6.57131 | 11.084 | 9.06E-09 | 3.732981388 |
| ENSG00000131652 | THOC6 | 48.3235 | 45.2746 | 50. 1 155 | 101. 135 | 95.4469 | 122.812 | 3.20E-10 | 1. 162047892 |
| ENSG00000131746 | TNS4 | 57.3308 | 32.3732 | 29.9147 | 118.079 | 89.8712 | 114.258 | 6.09E-08 | 1.417070825 |
| ENSG00000131876 | SNRPA1 | 53.2002 | 41.4099 | 34.5116 | 105.969 | 95.8018 | 137.477 | 2.52E-05 | 1.410424235 |
| ENSG00000132170 | PPARG | 4.97057 | 5.61403 | 5.49286 | 13.9936 | 11.0363 | 17.9882 | 0.00096949 | 1.372057952 |
| ENSG00000132334 | PTPRE | 1.00195 | 0.68724 | 0.784166 | 2.7994 | 3.61612 | 2.48021 | 4.83E-06 | 1.543729484 |
| ENSG00000132470 | ITGB4 | 81.5452 | 73.5723 | 93. 1267 | 203.959 | 127. 138 | 444.58 | 0.01142636 | 1.385878703 |
| ENSG00000132613 | MTSS1L | 38.6936 | 36.6129 | 35.3526 | 55.3664 | 62.3554 | 68.6502 | 8.57E-05 | 1.05501326 |
| ENSG00000132702 | HAPLN2 | 0. 114889 | 0.255417 | 0.0918864 | 1.35736 | 0.65262 | 2.02124 | 0.00042979 | 3.208740646 |
| ENSG00000133083 | DCLK1 | 0 | 0.0921461 | 0.0594998 | 0.572617 | 0.261066 | 1.30036 | 0.0302662 | 4.009088226 |
| ENSG00000133226 | SRRM1 | 39.9825 | 39.2305 | 34.9087 | 78.81 | 76.2607 | 87. 1 145 | 8. 17E-08 | 1.056980818 |
| ENSG00000133321 | RARRES3 | 1. 11859 | 0.589889 | 1. 17699 | 4.38282 | 2.79387 | 2.90919 | 0.00084922 | 1.847176667 |

| ENSG00000134013 | LOXL2 | 0. 179787 | 0.281677 | 0.335307 | 1.76414 | 1.35087 | 5.53655 | 0.02752274 | 3.482436092 |
| --- | --- | --- | --- | --- | --- | --- | --- | --- | --- |
| ENSG00000134057 | CCNB1 | 77.6283 | 53.646 | 40.4934 | 122.463 | 129. 1 1 1 | 158.079 | 0.00064627 | 1.254034868 |
| ENSG00000134107 | BHLHE40 | 8.70053 | 7. 10479 | 9.60528 | 21.348 | 16.0868 | 22.6692 | 2.71E-07 | 1.256676093 |
| ENSG00000134222 | PSRC1 | 24.8172 | 20.9128 | 19. 1271 | 41.5853 | 41.7086 | 46.4374 | 8.44E-08 | 1.025845749 |
| ENSG00000134470 | IL15RA | 5.78629 | 6.34314 | 8.31723 | 15.5299 | 15.4056 | 26.2484 | 0.00064535 | 1.443951227 |
| ENSG00000134531 | EMP1 | 2. 13454 | 2.02643 | 1.25074 | 12.6854 | 10.6171 | 13.0975 | 4.02E-10 | 3.400588471 |
| ENSG00000134954 | ETS1 | 3.26853 | 3.05565 | 2.31061 | 10.8195 | 9.69752 | 9. 19541 | 1.47E-17 | 1.842223167 |
| ENSG00000134955 | SLC37A2 | 0.260574 | 0.613254 | 0.26 | 2.76801 | 1.6413 | 4.43103 | 0.00012401 | 3.514795699 |
| ENSG00000135045 | C9orf40 | 13.085 | 8.65994 | 9.34862 | 20.641 | 20.4964 | 23.3666 | 7.41E-07 | 1.072920114 |
| ENSG00000135074 | ADAM19 | 0.397643 | 0.514443 | 0.452605 | 6.5003 | 4.84015 | 6.63483 | 2.61E-27 | 3.659215505 |
| ENSG00000135127 | CCDC64 | 4.75359 | 3.00214 | 3.34097 | 10.981 | 9.09461 | 16.5808 | 1.89E-05 | 1.78610006 |
| ENSG00000135205 | CCDC146 | 0.525313 | 0.33832 | 0.376595 | 2.90105 | 2.40116 | 2.66588 | 1.99E-12 | 2.645894757 |
| ENSG00000135318 | NT5E | 2.39972 | 2.26661 | 2.46431 | 7.63107 | 5.55752 | 9.63987 | 0.00309884 | 1.677292976 |
| ENSG00000135378 | PRRG4 | 2.7774 | 3.21696 | 3.20857 | 7.042 | 8.29399 | 6.29573 | 2.61E-08 | 1.222983542 |
| ENSG00000135476 | ESPL1 | 12.7707 | 10.2034 | 7.89476 | 18.8498 | 22.3245 | 23.246 | 6.41E-08 | 1.081606332 |
| ENSG00000135605 | TEC | 1. 19276 | 1.29175 | 1.66857 | 2.63812 | 2.28397 | 3.58994 | 0.01189735 | 1.030485631 |
| ENSG00000135636 | DYSF | 0.251696 | 0.268889 | 0.223698 | 1.08483 | 0.743149 | 1.96594 | 0.00368053 | 2.350432603 |
| ENSG00000135678 | CPM | 0.22529 | 0.376839 | 0.531423 | 37.8116 | 14.9088 | 1.5128 | 2.61E-12 | 2.892447421 |
| ENSG00000135702 | CHST5 | 0.649215 | 0.84317 | 0.943829 | 2.09922 | 2.78703 | 3.03917 | 0.00760477 | 1.219401461 |
| ENSG00000135750 | KCNK1 | 16.8743 | 20. 1932 | 18.2524 | 38.7665 | 36.8319 | 47.2461 | 0.00022977 | 1.086492737 |
| ENSG00000135960 | EDAR | 1.83567 | 1.76003 | 1.2098 | 6.46537 | 5.90527 | 2.30738 | 0.02431049 | 1.596159094 |
| ENSG00000136108 | CKAP2 | 20.6614 | 18.7499 | 16.8933 | 38.6192 | 40.9934 | 35.7201 | 8.63E-08 | 1.00394094 |
| ENSG00000136153 | LMO7 | 5. 11099 | 6.2583 | 4. 15126 | 20. 1213 | 19.9291 | 19.9362 | 5. 19E-22 | 1.790817855 |
| ENSG00000136383 | ALPK3 | 0.659371 | 0.471542 | 0.518639 | 1.5205 | 1.49379 | 1.31035 | 7.00E-07 | 1.394849177 |
| ENSG00000136542 | GALNT5 | 0.972397 | 0.771884 | 0.788606 | 4.42333 | 3.85946 | 5.28265 | 1.26E-10 | 2.409964999 |
| ENSG00000136689 | IL1RN | 0.276192 | 0.277812 | 0.448618 | 1.76379 | 0.791053 | 0.938397 | 0.01573149 | 1.830631781 |
| ENSG00000136802 | LRRC8A | 19.0939 | 20. 1561 | 19.7282 | 38.6699 | 33.5458 | 52.6651 | 1.01E-05 | 1.072732404 |
| ENSG00000136999 | NOV | 1.97037 | 1.29468 | 0.953853 | 4.29427 | 2.83566 | 5.53479 | 0.00387459 | 1.579785005 |
| ENSG00000137203 | TFAP2A | 8.62148 | 8.49959 | 9.84384 | 17.7517 | 15.9523 | 29.8192 | 0.02665455 | 1.447283867 |
| ENSG00000137440 | FGFBP1 | 3.51024 | 1.33787 | 1.83096 | 10.5465 | 11.4346 | 8. 14512 | 1.22E-10 | 2. 144983809 |
| ENSG00000137449 | CPEB2 | 2.52875 | 2.68635 | 2.55851 | 6.77987 | 5.2812 | 6.94305 | 0.00073491 | 1.263612867 |
| ENSG00000137628 | DDX60 | 1.41971 | 0.329028 | 0.412375 | 1.22812 | 1.65972 | 2.77895 | 1.30E-06 | 1.947667243 |
| ENSG00000137699 | TRIM29 | 2. 16376 | 1.38581 | 1.53945 | 6.58209 | 5.21178 | 2.64936 | 0.00265021 | 1.613760339 |
| ENSG00000137726 | FXYD6 | 1.91032 | 1.90084 | 2.0835 | 7.41842 | 4.9846 | 6.49235 | 1.02E-07 | 1.883027885 |
| ENSG00000137807 | KIF23 | 16.9843 | 11.4582 | 10.8166 | 28.9813 | 31.4684 | 31.0277 | 2. 14E-11 | 1.24560074 |
| ENSG00000137819 | PAQR5 | 14.769 | 14.9915 | 11.9097 | 29.9172 | 27.8592 | 19. 1684 | 2.62E-08 | 1.041946596 |
| ENSG00000137822 | TUBGCP4 | 8.07427 | 7.25601 | 7.47974 | 15.6866 | 16. 1216 | 10.2023 | 3.85E-07 | 1.00862811 |
| ENSG00000137825 | ITPKA | 6.94201 | 6.31864 | 6.45162 | 12.7712 | 13.8261 | 18.3081 | 6. 19E-07 | 1. 191351973 |
| ENSG00000137831 | UACA | 3.65968 | 4.29703 | 3.50995 | 12.6987 | 10.0639 | 16.9565 | 0.00021404 | 1.711304614 |
| ENSG00000137878 | GCOM1 | 0.062869 | 0.352433 | 4.03E-05 | 0.571202 | 1.00439 | 0.625457 | 0.00121569 | 2.318564212 |
| ENSG00000137936 | BCAR3 | 4.45367 | 5. 10077 | 5. 13255 | 24.813 | 19. 1576 | 42.0707 | 6.58E-06 | 2.672254733 |
| ENSG00000137962 | ARHGAP29 | 8.0658 | 10.0392 | 6.68192 | 24.4553 | 21.3601 | 25.7699 | 2.26E-08 | 1.55787625 |
| ENSG00000138166 | DUSP5 | 7.72974 | 8.92649 | 9.28563 | 97.2181 | 45.5619 | 202.263 | 0.00765876 | 3.729100148 |

| ENSG00000138180 | CEP55 | 16.7988 | 14.4323 | 11.704 | 28.8878 | 31.8598 | 34.5236 | 7.67E-08 | 1.094434766 |
| --- | --- | --- | --- | --- | --- | --- | --- | --- | --- |
| ENSG00000138182 | KIF20B | 6.05952 | 5. 19572 | 3.65363 | 13.4489 | 15.0727 | 12.8571 | 1.36E-13 | 1.377031971 |
| ENSG00000138193 | PLCE1 | 0.296565 | 0.258754 | 0.328625 | 1.64689 | 1.49081 | 1.91264 | 5.73E-15 | 2.359761024 |
| ENSG00000138316 | ADAMTS14 | 0.875727 | 0.794234 | 0.745835 | 3.56771 | 2.22639 | 3.54727 | 8.35E-09 | 1.967713945 |
| ENSG00000138346 | DNA2 | 7. 13592 | 5.09087 | 6.47341 | 11.4468 | 13.6358 | 12.5747 | 1.87E-06 | 1.011174283 |
| ENSG00000138376 | BARD1 | 4.5536 | 3.80267 | 3.48222 | 8.51453 | 11.3341 | 8.51673 | 3.22E-06 | 1. 155153682 |
| ENSG00000138594 | TMOD3 | 12.327 | 13.8508 | 9.4337 | 22.214 | 25.943 | 20.7261 | 1.93E-08 | 1.048193402 |
| ENSG00000138623 | SEMA7A | 1.75822 | 1.68477 | 2.06299 | 30.7652 | 19.2606 | 99.291 | 0.01399291 | 4.836823764 |
| ENSG00000138639 | ARHGAP24 | 0 | 0.0314871 | 0. 134186 | 1.51054 | 1.79217 | 1.33925 | 5.46E-20 | 6.283783343 |
| ENSG00000138658 | ZGRF1 | 4.66825 | 4.55476 | 3.62443 | 7.00986 | 8.29508 | 10.4159 | 0.00014055 | 1. 111254239 |
| ENSG00000138744 | NAAA | 10.4736 | 8.9596 | 8.71494 | 20.6996 | 21.2027 | 16.4575 | 1.01E-05 | 1.053975501 |
| ENSG00000138772 | ANXA3 | 52.6767 | 62.2468 | 55. 1664 | 144.564 | 117.54 | 253.549 | 0.01068357 | 1.540962547 |
| ENSG00000139354 | GAS2L3 | 3.78258 | 4.69428 | 2. 12959 | 12.318 | 12.2039 | 6.39014 | 9.35E-06 | 1.508861243 |
| ENSG00000139734 | DIAPH3 | 6.61969 | 5.34589 | 3.73649 | 10.5806 | 12.3606 | 9.53916 | 2.39E-07 | 1.032320894 |
| ENSG00000139926 | FRMD6 | 5.0405 | 3. 15851 | 3. 17524 | 6.89503 | 7.20147 | 5.48518 | 5.22E-06 | 1.078957051 |
| ENSG00000140326 | CDAN1 | 4.64219 | 4.5212 | 5.96275 | 10.9285 | 12.3417 | 13.3713 | 6.23E-12 | 1.340258276 |
| ENSG00000140416 | TPM1 | 27.6991 | 24.2506 | 24.4874 | 57.2611 | 48.4485 | 54.3484 | 2.09E-09 | 1. 123135147 |
| ENSG00000140678 | ITGAX | 1.05737 | 1.27383 | 1.4886 | 2.87562 | 1.54802 | 2.6189 | 0.00727148 | 1. 100178561 |
| ENSG00000140853 | NLRC5 | 3.43463 | 2.33322 | 1.96008 | 9. 10014 | 8.04056 | 10. 196 | 4.23E-19 | 1.844303328 |
| ENSG00000140859 | KIFC3 | 27.572 | 36.3204 | 38.2907 | 62.4489 | 50.8707 | 86.3963 | 0.01220808 | 1.016815377 |
| ENSG00000141232 | TOB1 | 11.6317 | 11.7206 | 12.399 | 29.9689 | 25.3532 | 32.5836 | 1.31E-06 | 1.311141099 |
| ENSG00000141458 | NPC1 | 7.04569 | 8.51871 | 7. 14183 | 15.8073 | 12.7862 | 14.2326 | 1.09E-07 | 1.07485586 |
| ENSG00000141738 | GRB7 | 27.5023 | 25.9278 | 27.5592 | 62.9479 | 45.2332 | 85.7689 | 0.00120372 | 1.284988683 |
| ENSG00000142046 | TMEM91 | 6.46337 | 5.99917 | 7.50179 | 17. 1607 | 13.5056 | 26.0651 | 0.01245256 | 1.499654832 |
| ENSG00000142227 | EMP3 | 14. 1426 | 8.56484 | 14.7365 | 106.046 | 77.6402 | 138. 162 | 6.05E-12 | 3.058345283 |
| ENSG00000142233 | NTN5 | 0.833329 | 0.466343 | 0.907384 | 3. 19133 | 2.34901 | 2.76418 | 1.03E-05 | 2.016852903 |
| ENSG00000142408 | CACNG8 | 0. 130861 | 0.0410476 | 0.0379302 | 0.23892 | 0. 10866 | 0.26016 | 0.04050142 | 1.559431188 |
| ENSG00000142623 | PADI1 | 0.385057 | 0.458125 | 0.559319 | 18.5639 | 6.02155 | 60.7338 | 0.02082518 | 5.944026125 |
| ENSG00000142627 | EPHA2 | 58.3871 | 39.8206 | 47.7127 | 184.422 | 140.97 | 268.927 | 3.61E-06 | 2.030549181 |
| ENSG00000142733 | MAP3K6 | 7. 19339 | 5.20712 | 5.38406 | 14.5909 | 16.6701 | 18.6157 | 1.90E-10 | 1.271855199 |
| ENSG00000142798 | HSPG2 | 7.35257 | 11.8791 | 11.7377 | 21.0489 | 16.9133 | 30.3119 | 0.00067236 | 1. 145354812 |
| ENSG00000142871 | CYR61 | 6.64549 | 6.77419 | 5.34757 | 37.2906 | 20.7282 | 54.9454 | 0.0018169 | 2.578734802 |
| ENSG00000142910 | TINAGL1 | 25.6525 | 34.0615 | 30.8885 | 120.515 | 82.3338 | 230.547 | 0.00324783 | 2.220172784 |
| ENSG00000142945 | KIF2C | 26.2215 | 23. 1872 | 19.0199 | 43.9942 | 44.8157 | 50. 1191 | 6. 10E-08 | 1.008575915 |
| ENSG00000143127 | ITGA10 | 0.683984 | 0.294628 | 0.283029 | 1.56627 | 1.47254 | 1.8214 | 4.07E-07 | 1.989603286 |
| ENSG00000143153 | ATP1B1 | 39.2351 | 34.9191 | 39.8112 | 84. 1539 | 66.5787 | 116.808 | 0.00792955 | 1.24998434 |
| ENSG00000143228 | NUF2 | 14.3056 | 11.8657 | 8.72013 | 23.5176 | 22.7147 | 30. 1351 | 0.00130906 | 1.068833293 |
| ENSG00000143369 | ECM1 | 1.6669 | 0.93465 | 0.651582 | 2.22766 | 1.92278 | 4.45435 | 0.03888538 | 1.452757918 |
| ENSG00000143476 | DTL | 8.503 | 5.93959 | 5.32113 | 14.589 | 14.9699 | 12.9486 | 2.65E-08 | 1.082651603 |
| ENSG00000143507 | DUSP10 | 0.813433 | 0.866414 | 0.90281 | 1.75741 | 1.46388 | 2.45399 | 0.01013821 | 1. 146620502 |
| ENSG00000144063 | MALL | 7.25292 | 3.42044 | 2.94409 | 58.7009 | 49. 1463 | 58.5415 | 1.04E-63 | 3.659676307 |
| ENSG00000144152 | FBLN7 | 1.62272 | 2. 18297 | 3.06019 | 7.97391 | 5.62047 | 7.01543 | 7.78E-06 | 1.489160566 |
| ENSG00000144559 | TAMM41 | 9.76812 | 7.60929 | 7.25178 | 15.2353 | 12.7546 | 18.0191 | 0.00088036 | 1.035313341 |

| ENSG00000144821 | MYH15 | 0.514367 | 0.353574 | 0. 189179 | 0.713427 | 0.713108 | 0.923894 | 0.00634835 | 1. 102104452 |
| --- | --- | --- | --- | --- | --- | --- | --- | --- | --- |
| ENSG00000144959 | NCEH1 | 2.75645 | 3.30986 | 1.81405 | 6.06659 | 5.07944 | 6.09142 | 5.03E-06 | 1.204569867 |
| ENSG00000145014 | TMEM44 | 9.07976 | 9.42707 | 10.7023 | 20.431 | 14.8088 | 28.8525 | 0.00024898 | 1. 176717253 |
| ENSG00000145107 | TM4SF19 | 7.66371 | 10. 1671 | 7.75388 | 17.6256 | 17.0387 | 27.3273 | 0.00051575 | 1.37326003 |
| ENSG00000145113 | MUC4 | 0. 174236 | 0.318775 | 0.315907 | 0.989388 | 0.591303 | 1.89726 | 0.00222345 | 2.305390183 |
| ENSG00000145220 | LYAR | 23.3144 | 15.5067 | 14.7586 | 47.989 | 44.335 | 71.8517 | 0.00020465 | 1.567535814 |
| ENSG00000145287 | PLAC8 | 8.5917 | 10.3485 | 16.6505 | 73.6292 | 44.2619 | 89.7741 | 2.07E-08 | 2.062596488 |
| ENSG00000145386 | CCNA2 | 37.8721 | 21.7924 | 20.0096 | 76.8052 | 84.6757 | 65. 168 | 2.03E-12 | 1.488140617 |
| ENSG00000145632 | PLK2 | 5.96554 | 3.83989 | 3.47456 | 17. 1588 | 12.3379 | 15.4325 | 1. 17E-09 | 1.74086865 |
| ENSG00000146005 | PSD2 | 0. 133504 | 0. 199559 | 0. 15234 | 0.554984 | 0.478512 | 0.504422 | 0.00105605 | 1.698883258 |
| ENSG00000146072 | TNFRSF21 | 16. 1301 | 16.7696 | 14.8723 | 34.0194 | 28.4934 | 44.615 | 0.00031229 | 1. 173903974 |
| ENSG00000146090 | RASGEF1C | 1. 1 1 182 | 1.03008 | 1.27174 | 2.39394 | 2.34638 | 3.07441 | 0.00324706 | 1.200415711 |
| ENSG00000146281 | PM20D2 | 15.2668 | 17.5458 | 14.5175 | 36.2933 | 40.3619 | 19.5473 | 0.00383786 | 1.008000811 |
| ENSG00000146918 | NCAPG2 | 18.7564 | 16.0319 | 11.8641 | 33.8966 | 36.864 | 24.6072 | 5.25E-05 | 1.022134558 |
| ENSG00000147168 | IL2RG | 0.956947 | 0.548243 | 1.27842 | 3.31996 | 2. 1 188 | 4.31545 | 0.00448514 | 1.840947369 |
| ENSG00000147394 | ZNF185 | 3.94307 | 4.47015 | 4.92076 | 13.3785 | 9.95412 | 14.2182 | 9.53E-13 | 1.424825471 |
| ENSG00000147437 | GNRH1 | 0.615856 | 0.098048 | 1.73573 | 2.46204 | 1.45092 | 2.97297 | 0.00538665 | 2.296357553 |
| ENSG00000147874 | HAUS6 | 11.3851 | 11.8437 | 8.84455 | 26. 174 | 26.4877 | 34.2907 | 5.41E-05 | 1.450630756 |
| ENSG00000148344 | PTGES | 64.7688 | 49.9577 | 45.9132 | 126.383 | 100.486 | 146. 157 | 1. 12E-05 | 1.234924199 |
| ENSG00000148384 | INPP5E | 13.9269 | 13. 1996 | 14.381 | 31.8492 | 29.4481 | 45.3194 | 6.22E-09 | 1.386697552 |
| ENSG00000148426 | PROSER2 | 56.5585 | 52.0363 | 59.3144 | 139.927 | 93.5142 | 219.737 | 0.00061921 | 1.439825667 |
| ENSG00000148483 | TMEM236 | 0.051804 | 0.0148582 | 0.0334758 | 0.394298 | 0.462443 | 0.538068 | 6.56E-10 | 3.934623604 |
| ENSG00000148516 | ZEB1 | 0.238542 | 0.0542621 | 0.0846324 | 0.385142 | 0.292828 | 0.519395 | 0.01813041 | 1.464133163 |
| ENSG00000148671 | ADIRF | 2.03636 | 3.63537 | 2.24887 | 4.65367 | 4.31463 | 7.77341 | 0.02388678 | 1.065119264 |
| ENSG00000148677 | ANKRD1 | 0.202249 | 0. 188312 | 0.274858 | 18.6697 | 8.38171 | 45.9387 | 0.00126872 | 6.857342004 |
| ENSG00000149403 | GRIK4 | 0. 111699 | 0.0830729 | 0. 148382 | 0.249953 | 0.298011 | 0.206263 | 0.03223575 | 1.22095743 |
| ENSG00000149591 | TAGLN | 2.34116 | 2.33821 | 2.07494 | 5.95032 | 6.23899 | 9.5044 | 0.00040367 | 1.461163865 |
| ENSG00000149596 | JPH2 | 0.04844 | 0.0351903 | 0.0384594 | 1.77674 | 1.49658 | 2. 14579 | 9.45E-28 | 5.569300491 |
| ENSG00000149639 | SOGA1 | 4.43989 | 4. 18076 | 4.2887 | 10.2972 | 11.5586 | 9.2931 | 2.68E-12 | 1.289540933 |
| ENSG00000150782 | IL18 | 22.0764 | 28. 1728 | 34.0071 | 74.805 | 57.9816 | 113.445 | 0.01250531 | 1.580770435 |
| ENSG00000150938 | CRIM1 | 13.2143 | 15.6239 | 10.6022 | 50.5996 | 43.915 | 41.0609 | 8.99E-21 | 1.727118126 |
| ENSG00000151136 | BTBD11 | 0.979536 | 1.54943 | 0.9088 | 4. 12367 | 3.85709 | 3.96765 | 1.08E-10 | 1.765272832 |
| ENSG00000151689 | INPP1 | 13.9175 | 9.95467 | 10. 1949 | 23. 1728 | 19.71 | 31.9407 | 8. 14E-05 | 1.045878303 |
| ENSG00000152253 | SPC25 | 11.6404 | 8.8717 | 5. 19823 | 20.8552 | 17.4414 | 18.9874 | 9.36E-05 | 1. 15530921 |
| ENSG00000152465 | NMT2 | 2.62042 | 2.45018 | 2.46159 | 5.54982 | 4.98424 | 5.97468 | 0.00012953 | 1. 132295241 |
| ENSG00000152689 | RASGRP3 | 0.914324 | 0.21497 | 0.202698 | 0.471155 | 0.491442 | 0.657041 | 0.01734684 | 1.246239086 |
| ENSG00000152784 | PRDM8 | 0.044489 | 0.0640351 | 0.0190736 | 1.22043 | 0.939352 | 1.87348 | 2.89E-10 | 5.411984092 |
| ENSG00000153157 | SYCP2L | 0.017427 | 0. 11509 | 0 | 0.469226 | 0.35318 | 0.821981 | 0.00060833 | 3.730292152 |
| ENSG00000153292 | ADGRF1 | 0.246527 | 0. 133708 | 0. 183 | 0.820532 | 0.589608 | 1.21707 | 0.04386613 | 2.048776313 |
| ENSG00000153391 | INO80C | 22.2 | 12.5941 | 14.7127 | 48.2132 | 45.8156 | 49. 1303 | 4.71E-12 | 1.493363016 |
| ENSG00000153815 | CMIP | 16.6361 | 18.3692 | 15.7016 | 36.8381 | 34.0124 | 44.6703 | 4.20E-10 | 1.214725068 |
| ENSG00000153902 | LGI4 | 0. 105479 | 0.0630725 | 0. 106254 | 1.09366 | 0.619869 | 1.52282 | 3. 1 1E-05 | 3.631014593 |
| ENSG00000153933 | DGKE | 4.31687 | 6.45761 | 5.78966 | 9.74248 | 8.66185 | 9.05672 | 3.88E-08 | 1.060809372 |

| ENSG00000154102 | C16orf74 | 25.6819 | 22.8405 | 26.2541 | 57. 1952 | 45.5707 | 88.7414 | 0.0011492 | 1.328366364 |
| --- | --- | --- | --- | --- | --- | --- | --- | --- | --- |
| ENSG00000154127 | UBASH3B | 5.27584 | 4.22552 | 3.23011 | 17.7102 | 20.0871 | 11.9251 | 5.89E-10 | 1.939317087 |
| ENSG00000154274 | C4orf19 | 0.543063 | 0.592056 | 0.618502 | 1.64963 | 1.57981 | 1.61125 | 0.00259237 | 1.502887864 |
| ENSG00000154447 | SH3RF1 | 3.23442 | 4.04325 | 3.78597 | 8.85526 | 8.37785 | 8. 19485 | 4.79E-09 | 1. 198850699 |
| ENSG00000155066 | PROM2 | 3.2872 | 3.79387 | 4.07345 | 9.71634 | 9.02779 | 5.73286 | 0.00134883 | 1. 176902426 |
| ENSG00000155324 | GRAMD3 | 4.65236 | 4.7058 | 4. 15415 | 10.9484 | 10.0174 | 18.3827 | 0.00737295 | 1.533951946 |
| ENSG00000156273 | BACH1 | 9.08348 | 10.3393 | 8.48086 | 21.7009 | 18.2336 | 19.3349 | 5.51E-09 | 1.087192113 |
| ENSG00000156381 | ANKRD9 | 29. 1626 | 26.3198 | 32.765 | 75.3284 | 54.6789 | 118.805 | 2.22E-05 | 1.59763288 |
| ENSG00000156500 | FAM122C | 1.23004 | 1.4358 | 1.2148 | 3. 13573 | 2.75586 | 3. 10062 | 0.0114353 | 1. 192684126 |
| ENSG00000156504 | FAM122B | 20.8425 | 20.4236 | 17.6381 | 39.8926 | 40.2351 | 34.6198 | 9.49E-08 | 1.000839203 |
| ENSG00000156802 | ATAD2 | 16.4045 | 11.8731 | 8.95161 | 29.9243 | 33.9628 | 24.6486 | 1.09E-07 | 1. 199427932 |
| ENSG00000156970 | BUB1B | 11.2536 | 8.00351 | 5.95195 | 27.0706 | 30.2338 | 28.9473 | 8.26E-21 | 1.733307843 |
| ENSG0000015711 1 | TMEM171 | 2.49001 | 1.92468 | 2.33735 | 5.09534 | 4.48653 | 9.80274 | 0.03281568 | 1.508174129 |
| ENSG00000157193 | LRP8 | 10.5215 | 9.20994 | 6.4075 | 23.7419 | 27.58 | 22.2425 | 2.36E-15 | 1.468902145 |
| ENSG00000157538 | DSCR3 | 16.037 | 13.0991 | 11. 1561 | 30.8135 | 29.4839 | 25.2131 | 8. 1 1E-08 | 1.011912072 |
| ENSG00000157617 | C2CD2 | 5.93287 | 6.09763 | 6.96163 | 15.7189 | 16.6088 | 15.344 | 3.28E-12 | 1.292122948 |
| ENSG00000158711 | ELK4 | 5.49092 | 5. 10977 | 3.2654 | 11.5255 | 12.5596 | 8.93694 | 8.88E-13 | 1.327774037 |
| ENSG00000159055 | MIS18A | 22.244 | 18.7328 | 15.551 | 37.8956 | 42.5314 | 41.6543 | 1. 14E-08 | 1. 11755476 |
| ENSG00000159086 | PAXBP1 | 10. 1767 | 9.30509 | 9.83262 | 20.9136 | 21. 123 | 21.5857 | 5.01E-09 | 1.09827756 |
| ENSG00000159147 | DONSON | 16.459 | 15.2729 | 12. 1527 | 45.5598 | 44.7439 | 45.0692 | 4.93E-18 | 1.621788002 |
| ENSG00000159259 | CHAF1B | 10.6809 | 8.96961 | 7.0902 | 17.5231 | 17.8986 | 19.2448 | 4.87E-08 | 1.033104196 |
| ENSG00000159784 | FAM131B | 6.03409 | 7.60279 | 8.58262 | 17.51 1 1 | 13.7941 | 17.7837 | 6.28E-09 | 1.20209615 |
| ENSG00000159871 | LYPD5 | 2.9646 | 3.03021 | 3.58004 | 11.0946 | 7.9708 | 10.4457 | 3.91E-10 | 1.756308735 |
| ENSG00000159899 | NPR2 | 1.31281 | 0.694396 | 1.864 | 2.22479 | 2.33963 | 2.00736 | 0.00122333 | 1.297446848 |
| ENSG00000160117 | ANKLE1 | 1.01 1 1 1 | 0.930444 | 1.3501 | 1.93791 | 1.93844 | 3.3004 | 0.00956414 | 1. 138006386 |
| ENSG00000160183 | TMPRSS3 | 0.897936 | 0.660291 | 0.802535 | 3.43461 | 2. 1366 | 4.00637 | 0.00039721 | 1.99453287 |
| ENSG00000160255 | ITGB2 | 0.234 | 0. 160064 | 0. 161677 | 1.30237 | 0.778627 | 0.855735 | 0.00112572 | 1.764643372 |
| ENSG00000160305 | DIP2A | 9.05592 | 9.54681 | 8.49009 | 17.3122 | 16.4458 | 22.9756 | 8.47E-06 | 1. 141598676 |
| ENSG00000160752 | FDPS | 185.964 | 117.241 | 115.73 | 313.444 | 283.726 | 408.092 | 3.58E-05 | 1.231606886 |
| ENSG00000161243 | FBXO27 | 0.261256 | 0.433969 | 0.627892 | 10.8542 | 3.5593 | 7.78278 | 0.00157144 | 3.626010795 |
| ENSG00000161509 | GRIN2C | 0.347348 | 0. 172126 | 0.277543 | 1.3861 | 1. 15278 | 3. 13306 | 0.00258836 | 3.004443195 |
| ENSG00000162148 | PPP1R32 | 1.3965 | 1. 16851 | 1.26545 | 3.69589 | 3.94343 | 8.85752 | 0.0113034 | 1.776482073 |
| ENSG00000162496 | DHRS3 | 7.23911 | 13.9823 | 17.3521 | 33.5507 | 27.2453 | 46.4977 | 0.00012518 | 1.488639239 |
| ENSG00000162552 | WNT4 | 0.215248 | 0.545995 | 0.536689 | 1.31764 | 0.966609 | 1. 19449 | 0.00034019 | 1.694245895 |
| ENSG00000162591 | MEGF6 | 4.05293 | 4.52331 | 7.42298 | 15.2474 | 9.77099 | 23. 13 | 0.02564776 | 1.61640725 |
| ENSG00000162616 | DNAJB4 | 4.3426 | 4.26932 | 4.01117 | 10.453 | 11.3062 | 11.9086 | 6.60E-09 | 1.46555011 1 |
| ENSG00000162745 | OLFML2B | 0.306232 | 0. 12553 | 0. 134604 | 0.624154 | 0.459047 | 0.757278 | 0.00166645 | 1.703946515 |
| ENSG00000162783 | IER5 | 12.0798 | 11. 1897 | 11.9845 | 32.7336 | 24. 1062 | 32.2747 | 2.06E-08 | 1.371358589 |
| ENSG00000162929 | KIAA1841 | 1.76001 | 2.29462 | 1.78783 | 4.7659 | 4.8344 | 3.79087 | 2.94E-05 | 1. 17156112 |
| ENSG00000163083 | INHBB | 0.424687 | 0.590583 | 0.787987 | 3.23123 | 1.87109 | 4.05825 | 5.80E-05 | 2.363032589 |
| ENSG00000163110 | PDLIM5 | 6.62064 | 8. 14899 | 5.85139 | 23.9215 | 13.3722 | 15. 1382 | 4.75E-06 | 1.322610025 |
| ENSG00000163132 | MSX1 | 18.7361 | 15.6284 | 19.7788 | 39.0706 | 35.545 | 53.046 | 7.96E-08 | 1.266991542 |
| ENSG00000163141 | BNIPL | 0.391405 | 0. 1 18364 | 0. 119167 | 0.834976 | 0.524203 | 0.877677 | 0.00078894 | 1.916039039 |

| ENSG00000163171 | CDC42EP3 | 5. 14023 | 7.94468 | 6.8587 | 13.8203 | 14.0168 | 12. 1692 | 2.71E-07 | 1.041003779 |
| --- | --- | --- | --- | --- | --- | --- | --- | --- | --- |
| ENSG00000163293 | NIPAL1 | 1.80915 | 1. 13882 | 0.856667 | 4.32291 | 4.45771 | 3.92248 | 4.68E-11 | 1.853148032 |
| ENSG00000163297 | ANTXR2 | 2.40174 | 2.3339 | 1.51405 | 2.68576 | 2.98181 | 2.29274 | 0.00070958 | 1. 129601195 |
| ENSG00000163507 | KIAA1524 | 6.20466 | 5.25774 | 2.9056 | 10.5954 | 12.0013 | 8.75009 | 5.92E-07 | 1. 142096458 |
| ENSG00000163535 | SGOL2 | 3.4839 | 2.93945 | 2.37501 | 5.39833 | 6.99489 | 6.00187 | 1.20E-05 | 1.053581741 |
| ENSG00000163918 | RFC4 | 36.5486 | 33.0412 | 28.8269 | 70.9773 | 71. 1604 | 65.5836 | 2.76E-08 | 1.051019314 |
| ENSG00000164038 | SLC9B2 | 6.63365 | 5.55523 | 3.88636 | 10.9767 | 12. 196 | 9.03457 | 1.99E-05 | 1.015014627 |
| ENSG00000164104 | HMGB2 | 200.093 | 144.61 | 137.77 | 313.064 | 310.569 | 381.968 | 1.42E-06 | 1.003106203 |
| ENSG00000164109 | MAD2L1 | 21.2637 | 16.0025 | 12.6372 | 35.5607 | 36.9696 | 29.6826 | 5.36E-08 | 1.014227007 |
| ENSG00000164236 | ANKRD33B | 0.746477 | 0.802092 | 0.66181 | 1.29215 | 1.61929 | 1.66751 | 0.00055463 | 1.06858327 |
| ENSG00000164362 | TERT | 0. 133945 | 0.29186 | 0.399089 | 0.999778 | 0.773818 | 1.27088 | 1.70E-05 | 2.025639786 |
| ENSG00000164400 | CSF2 | 0 | 0. 103159 | 0 | 5.75061 | 11.6066 | 10.0896 | 8. 18E-15 | 8. 156177388 |
| ENSG00000164611 | PTTG1 | 112. 122 | 71.267 | 62.4526 | 183.726 | 187.632 | 231.208 | 6.38E-06 | 1.281055067 |
| ENSG00000164674 | SYTL3 | 0.507557 | 0.734659 | 0.498868 | 2.84398 | 1.83226 | 6.96729 | 0.04116949 | 2.645221287 |
| ENSG00000164741 | DLC1 | 3.01148 | 2.92645 | 1.73438 | 5.82304 | 5.7963 | 5. 16092 | 6.48E-08 | 1. 174036836 |
| ENSG00000164744 | SUN3 | 0. 164304 | 0.519783 | 0.819868 | 3.29655 | 1.88833 | 4.58197 | 0.00164936 | 2.906942669 |
| ENSG00000164867 | NOS3 | 1.01458 | 0.630875 | 0.646792 | 1.55324 | 1. 13154 | 2.3702 | 0.00766463 | 1.228249256 |
| ENSG00000164877 | MICALL2 | 15.8164 | 16.0107 | 14.9886 | 28. 1 146 | 27.5218 | 38.7726 | 3.78E-08 | 1.030650739 |
| ENSG00000164949 | GEM | 1.51064 | 2.77244 | 2. 1699 | 4.4821 | 4.99757 | 7.54547 | 0.00076645 | 1.351463643 |
| ENSG00000165244 | ZNF367 | 8.89836 | 8. 12038 | 6.25958 | 20.6849 | 20.4019 | 15.443 | 3.07E-11 | 1.288416279 |
| ENSG00000165272 | AQP3 | 22. 1043 | 24.3695 | 19.3841 | 735.976 | 477.454 | 690.397 | 3.71E-30 | 4.872350604 |
| ENSG00000165810 | BTNL9 | 4.25222 | 3.51683 | 3.82597 | 8.07859 | 6.64149 | 9.24754 | 5.57E-05 | 1.007670033 |
| ENSG00000165887 | ANKRD2 | 0. 1 16548 | 0.0542268 | 0 | 1.53647 | 1. 14414 | 2.62241 | 7.76E-08 | 5. 140465552 |
| ENSG00000165891 | E2F7 | 5.37255 | 4.54813 | 3.42667 | 10.0628 | 11.5552 | 6.44012 | 0.00247911 | 1.043482343 |
| ENSG00000165923 | AGBL2 | 0.543275 | 0.552142 | 0.335988 | 0.72517 | 1.25682 | 0.98196 | 0.0180554 | 1.341020766 |
| ENSG00000166025 | AMOTL1 | 0.006311 | 0.0135283 | 0.0106842 | 0.611934 | 0.390704 | 0.549972 | 4.04E-20 | 5.970149535 |
| ENSG00000166147 | FBN1 | 0.055664 | 0.0796299 | 0.0197958 | 0.269656 | 0.246167 | 0.27783 | 3.54E-07 | 2.656320959 |
| ENSG00000166250 | CLMP | 0.657113 | 0.709074 | 0.841369 | 2.82406 | 2.94073 | 2.72936 | 2.42E-09 | 1.932645481 |
| ENSG00000166343 | MSS51 | 1.58326 | 1.0782 | 1.20791 | 3.03205 | 2.94308 | 3.98761 | 0.00066216 | 1.373466823 |
| ENSG00000166473 | PKD1L2 | 0.27234 | 0.583442 | 0.559957 | 2.98374 | 1.96823 | 4.54042 | 0.00010463 | 3.036372255 |
| ENSG00000166546 | BEAN1 | 1.02969 | 1.05917 | 2.23589 | 4.48109 | 3.75792 | 2.92604 | 0.00038529 | 1.379142844 |
| ENSG00000166578 | IQCD | 2.47093 | 2.92644 | 2.78062 | 5.70872 | 4. 10204 | 8.32156 | 0.00874483 | 1.096642076 |
| ENSG00000166813 | KIF7 | 10.7445 | 9.29865 | 8.52307 | 16.3472 | 14.7215 | 27.4303 | 0.00397218 | 1.031890029 |
| ENSG00000166819 | PLIN1 | 0. 109995 | 0.279266 | 0.685333 | 0.553028 | 0.682715 | 0.775338 | 0.02580808 | 1.231902446 |
| ENSG00000166823 | MESP1 | 2.7507 | 1.25708 | 2.09046 | 4.62838 | 4. 14097 | 5. 10529 | 0.00057968 | 1.230446732 |
| ENSG00000166833 | NAV2 | 1.32378 | 1.21232 | 0.982976 | 7.45513 | 6.23776 | 9. 10712 | 9.49E-15 | 2.795928251 |
| ENSG00000166845 | C18orf54 | 1.67747 | 2.04521 | 1.27747 | 3.65508 | 3.99029 | 3.51922 | 8.20E-05 | 1. 17030872 |
| ENSG00000166851 | PLK1 | 51.8305 | 32.7791 | 26.8815 | 71.4072 | 77.7394 | 90.611 1 | 2.22E-06 | 1. 12254952 |
| ENSG00000166924 | NYAP1 | 2.89636 | 4.72564 | 5.87297 | 8.05561 | 5.99441 | 13.2281 | 0.03997438 | 1.055295442 |
| ENSG00000166949 | SMAD3 | 13.6579 | 13.2931 | 10.7918 | 33.3419 | 29.6746 | 36.4528 | 1.94E-10 | 1.720448382 |
| ENSG00000166979 | EVA1C | 14.6815 | 14.9989 | 16.6107 | 62.5644 | 46.9665 | 77.4729 | 3.36E-09 | 2.015048617 |
| ENSG00000167081 | PBX3 | 2.03122 | 1.73355 | 1.63553 | 4. 10765 | 3.9858 | 5.43364 | 9. 19E-05 | 1.316956638 |
| ENSG00000167165 | UGT1A6 | 0.856936 | 0.434876 | 2.57E-15 | 13.3647 | 17.5016 | 3.03264 | 0.00017128 | 5.280098055 |

| ENSG00000167191 | GPRC5B | 2.29797 | 3.06013 | 1.94172 | 5.98721 | 5.37682 | 8.87491 | 0.01354658 | 1.405081442 |
| --- | --- | --- | --- | --- | --- | --- | --- | --- | --- |
| ENSG00000167264 | DUS2 | 8.98999 | 7.57367 | 8.09346 | 19.6479 | 17.3922 | 25.3009 | 1. 13E-05 | 1.241858679 |
| ENSG00000167653 | PSCA | 1.59922 | 0.911914 | 0.447885 | 6.21385 | 4.36113 | 13.7858 | 0.00636876 | 3.054904892 |
| ENSG00000102871 | TRADD | 22.2296 | 26.5264 | 32.4162 | 16.3194 | 14.5294 | 18.9 | 0.01135694 | -0.69885621 |
| ENSG00000213341 | CHUK | 7.31279 | 8.45093 | 6.77841 | 11.3883 | 12.2233 | 8.03668 | 0.04113217 | 0.45776853 |
| ENSG00000137275 | RIPK1 | 8.6476 | 7.42954 | 7.35554 | 8.2648 | 9.91698 | 9.0192 | 0.48674896 | 0. 171724893 |
| ENSG00000167656 | LY6D | 2.88577 | 1.67426 | 1.29198 | 11.5153 | 15.6091 | 10.6003 | 6.67E-11 | 2.751653447 |
| ENSG00000167766 | ZNF83 | 4.76816 | 6.48659 | 5.67513 | 11.51 1 1 | 9.60657 | 12.0848 | 1. 13E-05 | 1.000988152 |
| ENSG00000167889 | MGAT5B | 0. 132637 | 0.0758515 | 0. 10415 | 0.424316 | 0.400003 | 0.502964 | 3.57E-05 | 2.306748402 |
| ENSG00000167904 | TMEM68 | 11. 1171 | 14.6144 | 11.67 | 23.5709 | 20.6463 | 23.0414 | 1.88E-05 | 1.041825937 |
| ENSG00000167977 | KCTD5 | 30.0429 | 28. 1654 | 24.6005 | 56.9023 | 41.8781 | 70.3356 | 0.00110001 | 1.046534809 |
| ENSG00000168077 | SCARA3 | 13.3159 | 12.8986 | 13. 1261 | 40.584 | 36.3471 | 38.6101 | 2. 13E-16 | 1.515679644 |
| ENSG00000168078 | PBK | 27.4309 | 22.6917 | 15.3772 | 58.2767 | 59.6795 | 56.697 | 9.04E-15 | 1.432468261 |
| ENSG00000168490 | PHYHIP | 1.24531 | 0.609429 | 0.605543 | 1.65318 | 1.93954 | 3. 17809 | 0.01334296 | 1. 161527741 |
| ENSG00000168528 | SERINC2 | 64.9945 | 68.5118 | 82.4615 | 220.935 | 143.812 | 371.381 | 0.00941072 | 1.783887418 |
| ENSG00000168675 | LDLRAD4 | 0.416672 | 0. 170838 | 0.208603 | 3. 19625 | 1.67837 | 2.95366 | 1. 13E-08 | 3.033823912 |
| ENSG00000168779 | SHOX2 | 1.36521 | 1. 19371 | 1.28777 | 4. 10561 | 2.68115 | 4.05975 | 2.31E-05 | 1.597071426 |
| ENSG00000169035 | KLK7 | 13.2142 | 14. 122 | 16.361 | 34.8391 | 27.3834 | 29. 1223 | 2.36E-05 | 1.057864837 |
| ENSG00000169174 | PCSK9 | 1.53642 | 0.296483 | 0.606828 | 7.27051 | 6.87392 | 4.81898 | 5. 1 1E-11 | 3.011438914 |
| ENSG00000169188 | APEX2 | 16.3838 | 11.7566 | 10.0403 | 38.4831 | 32.9868 | 49.8281 | 1.30E-06 | 1.672015133 |
| ENSG00000169213 | RAB3B | 0.893427 | 1.08601 | 0.776965 | 9.02505 | 7.8164 | 8.61647 | 9.21E-62 | 3.200976781 |
| ENSG00000169247 | SH3TC2 | 0. 167568 | 0. 152669 | 0.0124811 | 0.855784 | 1.31239 | 1.37422 | 2.27E-10 | 3.33496569 |
| ENSG00000169330 | KIAA1024 | 0.260355 | 0. 190789 | 0. 128104 | 0.457307 | 0.408364 | 0.572562 | 0.00776625 | 1.312933081 |
| ENSG00000169403 | PTAFR | 0.650262 | 0.718522 | 0.588737 | 4. 12105 | 2.53371 | 1.6318 | 3.07E-05 | 2. 102272607 |
| ENSG00000169607 | CKAP2L | 4.68764 | 3.70149 | 2.7708 | 8.56121 | 9.31135 | 8. 13207 | 1.34E-08 | 1. 175979822 |
| ENSG00000169679 | BUB1 | 13.5359 | 10.319 | 7.53903 | 27.3697 | 30.6652 | 30.0019 | 9.06E-15 | 1.459587807 |
| ENSG00000169710 | FASN | 113.664 | 54. 1314 | 48.2892 | 148.334 | 159.607 | 110. 161 | 0.02321177 | 1.006851416 |
| ENSG00000169715 | MT1E | 374.908 | 188.405 | 248.086 | 643.308 | 412. 192 | 781.792 | 0.00507231 | 1.206085559 |
| ENSG00000169744 | LDB2 | 0.26884 | 0.0797945 | 0.702581 | 4.52369 | 2.614 | 4. 10659 | 5.66E-14 | 4.551350119 |
| ENSG00000169992 | NLGN2 | 23.9269 | 20.5491 | 18.6276 | 51.7943 | 49.3945 | 79.0455 | 3.26E-05 | 1.609433524 |
| ENSG00000170075 | GPR37L1 | 0.690555 | 0.422465 | 0.26181 | 1. 15929 | 0.944184 | 0.684297 | 0.02780908 | 1.038549866 |
| ENSG00000170312 | CDK1 | 58.5808 | 46.706 | 39.0531 | 127.077 | 120.088 | 140.733 | 1.90E-06 | 1.44445426 |
| ENSG00000175104 | TRAF6 | 1.82992 | 2.52316 | 2.76474 | 1.97518 | 2.52613 | 1.86062 | 0.7771791 | 0. 126512591 |
| ENSG00000170667 | RASA4B | 0.229704 | 0.0662496 | 0.22109 | 0.63166 | 0.499083 | 0.936196 | 0.00029197 | 2.000417756 |
| ENSG00000170734 | POLH | 2.42643 | 3.02957 | 2.5369 | 5.47227 | 6.41749 | 5.07681 | 9.87E-05 | 1.06591596 |
| ENSG00000170873 | MTSS1 | 2.44805 | 2.36533 | 2.68309 | 6.54202 | 5.72922 | 4.97979 | 6.63E-05 | 1.047046933 |
| ENSG00000171055 | FEZ2 | 14. 1348 | 11.7087 | 12.9939 | 28.9205 | 24.0935 | 37. 1249 | 0.00069122 | 1. 174042209 |
| ENSG00000171097 | CCBL1 | 4.64037 | 4.33431 | 4.2664 | 16.0436 | 13.3393 | 21.246 | 5.64E-09 | 1.833897397 |
| ENSG00000171320 | ESCO2 | 3.52193 | 3.72535 | 2.05831 | 6.76205 | 9.09403 | 4.63147 | 0.00053981 | 1.275577871 |
| ENSG00000171346 | KRT15 | 2.07781 | 1.80175 | 2. 11559 | 6.76623 | 5.3182 | 9.31002 | 1.40E-06 | 1.783908798 |
| ENSG00000171401 | KRT13 | 0. 168925 | 0.099071 | 0 | 6.97493 | 2.4766 | 20.5487 | 0.01129884 | 6.733577226 |
| ENSG00000171522 | PTGER4 | 0.260886 | 0.300221 | 0.237185 | 1.21528 | 1.22105 | 0.704217 | 2.34E-05 | 2.026381971 |
| ENSG00000171552 | BCL2L1 | 105.283 | 109.4 | 102.03 | 223.227 | 168.571 | 253.904 | 0.00043483 | 1.093557221 |

| ENSG00000171790 | SLFNL1 | 0.59849 | 0.793872 | 0.511167 | 1.26104 | 1.61214 | 1.41867 | 0.00036216 | 1.525766116 |
| --- | --- | --- | --- | --- | --- | --- | --- | --- | --- |
| ENSG00000171903 | CYP4F11 | 2.22544 | 4.40799 | 5. 13776 | 9.80549 | 7.65786 | 6. 13018 | 0.01073707 | 1.011708598 |
| ENSG00000171960 | PPIH | 61. 1084 | 30.6379 | 32.2913 | 89.7542 | 81. 101 | 80.7641 | 0.00055896 | 1.014407301 |
| ENSG00000172137 | CALB2 | 0. 149708 | 0.2047 | 0. 197286 | 2.06982 | 0.795895 | 3.71061 | 0.00758298 | 3.767842418 |
| ENSG00000172159 | FRMD3 | 1.37214 | 1.87208 | 2.31731 | 4.38956 | 4. 12615 | 3.02365 | 0.00023169 | 1.029572977 |
| ENSG00000172175 | MALT1 | 9.5946 | 10.9972 | 9.79322 | 23.8427 | 24.3991 | 19.2583 | 8.43E-09 | 1.078366018 |
| ENSG00000172731 | LRRC20 | 12.4357 | 13.8183 | 13.6124 | 27. 1 161 | 28.5716 | 25.6816 | 2.26E-08 | 1.071243957 |
| ENSG00000172789 | HOXC5 | 1.00046 | 0.880447 | 0.563918 | 1.79128 | 1.7792 | 2.50817 | 0.00600377 | 1.352008277 |
| ENSG00000172893 | DHCR7 | 70.2477 | 46.8981 | 42.6073 | 116.588 | 129.225 | 93.3291 | 2.32E-05 | 1. 126353808 |
| ENSG00000172927 | MYEOV | 5.2719 | 6.73176 | 7.68716 | 18.5285 | 13.0827 | 29.3444 | 0.01166328 | 1.654139313 |
| ENSG00000173209 | AHSA2 | 11.3517 | 10.9035 | 8.92646 | 21.9759 | 21.6442 | 26. 1223 | 7.42E-08 | 1.010327699 |
| ENSG00000173210 | ABLIM3 | 0. 194209 | 0.298666 | 0.371721 | 1. 1 148 | 0.556965 | 0.899314 | 0.00198944 | 1.761180915 |
| ENSG00000173267 | SNCG | 16.0882 | 14.8393 | 18.6381 | 207.653 | 155.679 | 264.544 | 3.39E-17 | 3.656091245 |
| ENSG00000173269 | MMRN2 | 1.77078 | 1.53525 | 1.85677 | 3.76406 | 4.02718 | 7.62041 | 1.34E-06 | 2.218240358 |
| ENSG00000173432 | SAA1 | 3.70168 | 2.08868 | 1.56027 | 14.9099 | 12.3952 | 40.0738 | 0.01976879 | 3.280215184 |
| ENSG00000173546 | CSPG4 | 0.059686 | 0. 1 1 1224 | 0.0653327 | 0. 193137 | 0.299599 | 0.498829 | 0.00619842 | 2.093648151 |
| ENSG00000173581 | CCDC106 | 0.82639 | 0.891373 | 1.75809 | 2.23646 | 2. 17294 | 3.6703 | 0.00891989 | 1.244478846 |
| ENSG00000173706 | HEG1 | 0.808749 | 1.04877 | 0.858941 | 2.82191 | 2.60335 | 1.90222 | 1.04E-06 | 1.358253036 |
| ENSG00000173926 | 3-Mar | 0. 193027 | 0.343927 | 0. 1 13234 | 0.372355 | 0.714401 | 0.283562 | 0.01439192 | 1.398799043 |
| ENSG00000174371 | EXO1 | 12.8743 | 7.97175 | 5.43944 | 16.3669 | 22.9003 | 16.5143 | 3.54E-08 | 1. 178309756 |
| ENSG00000175063 | UBE2C | 179.746 | 127.502 | 111.383 | 265.028 | 225.054 | 369.962 | 0.00520358 | 1.042444436 |
| ENSG00000175170 | FAM182B | 0.444141 | 0.676 | 0.366973 | 2.04571 | 2. 16678 | 1.49391 | 0.02154743 | 1.264025178 |
| ENSG00000175287 | PHYHD1 | 0.275202 | 0.302311 | 0.503231 | 5.02047 | 4.0993 | 14.4828 | 0.00219964 | 5.007896472 |
| ENSG00000175315 | CST6 | 9.97332 | 5. 14541 | 6.50647 | 147.769 | 101.672 | 243.68 | 1.87E-09 | 4.531000866 |
| ENSG00000175334 | BANF1 | 130.683 | 92.6738 | 105.712 | 252.562 | 218.012 | 327.832 | 1.88E-06 | 1.283015823 |
| ENSG00000175376 | EIF1AD | 12.2647 | 11.8168 | 12.7335 | 26.8059 | 24.6971 | 29. 1529 | 1.93E-08 | 1. 180902057 |
| ENSG00000175505 | CLCF1 | 5.9612 | 3.31631 | 4.48349 | 9.53742 | 9. 1306 | 17.6643 | 0.01694501 | 1.389069314 |
| ENSG00000175550 | DRAP1 | 147.255 | 118. 1 14 | 135.761 | 348.937 | 267.582 | 652.62 | 0.00527421 | 1.623200663 |
| ENSG00000175573 | C11orf68 | 24. 1 126 | 22.641 | 26.8025 | 59.8444 | 46.3423 | 79.565 | 2.43E-05 | 1.331346656 |
| ENSG00000175592 | FOSL1 | 18.2614 | 9.51354 | 9. 17806 | 317.52 | 196.099 | 575.778 | 6.46E-06 | 4.937108402 |
| ENSG00000175711 | B3GNTL1 | 6.42565 | 4.60413 | 5. 1 1566 | 11.375 | 12.2654 | 15. 1371 | 1.39E-05 | 1.263000985 |
| ENSG00000175745 | NR2F1 | 8.0602 | 6.08688 | 6.57257 | 18.9817 | 15.4144 | 32.4986 | 0.00060616 | 1.778081551 |
| ENSG00000175793 | SFN | 77.4626 | 56.5129 | 51.2217 | 258.306 | 214.617 | 379.353 | 7.83E-09 | 2.221135193 |
| ENSG00000176014 | TUBB6 | 65.2026 | 46.3039 | 43.972 | 135.06 | 127.895 | 221. 127 | 0.00037796 | 1.639867104 |
| ENSG00000176092 | AIM1L | 6.38725 | 5. 1623 | 5.73228 | 13.0392 | 11.396 | 15.4085 | 1.46E-07 | 1.046660657 |
| ENSG00000176170 | SPHK1 | 9.79364 | 4.95227 | 6.75076 | 21.387 | 16.2601 | 28.5114 | 1.53E-06 | 1.71425762 |
| ENSG00000176208 | ATAD5 | 2.58982 | 2. 14595 | 1.67982 | 7.93429 | 8.22066 | 7.04255 | 1. 18E-19 | 1.809683727 |
| ENSG00000176225 | RTTN | 4.23593 | 2.48929 | 2. 19548 | 10. 1355 | 7.09475 | 10.9599 | 4.73E-13 | 1.48166052 |
| ENSG00000176383 | B3GNT4 | 1.94101 | 1.78191 | 1.97691 | 5.3244 | 4.50274 | 5.32839 | 9.95E-05 | 1.40455253 |
| ENSG00000176387 | HSD11B2 | 4.67358 | 3.01688 | 2.61685 | 6.41569 | 7.26936 | 9.54763 | 0.00020507 | 1. 190937678 |
| ENSG00000176454 | LPCAT4 | 16.9193 | 16.0197 | 11.8907 | 39.2934 | 27.06 | 52.9366 | 0.00142955 | 1.472088466 |
| ENSG00000176533 | GNG7 | 0.285619 | 0.205864 | 0. 154333 | 1.33129 | 1. 16941 | 2. 14811 | 1.71E-06 | 2.908890583 |
| ENSG00000176678 | FOXL1 | 1.92321 | 1.06545 | 0.643085 | 20.8623 | 12.6065 | 26.2613 | 9.80E-08 | 4.204782042 |

| ENSG00000176697 | BDNF | 0. 149234 | 0. 130156 | 0. 133605 | 1.95294 | 2.09868 | 2.22713 | 1.48E-18 | 3.956961359 |
| --- | --- | --- | --- | --- | --- | --- | --- | --- | --- |
| ENSG00000176890 | TYMS | 88.0303 | 56.2492 | 50.4959 | 142.997 | 145.678 | 131.084 | 9.63E-09 | 1. 101190197 |
| ENSG00000176928 | GCNT4 | 0.038165 | 0.0296929 | 0 | 0.45036 | 0.263293 | 0.355276 | 8.79E-09 | 4.017453316 |
| ENSG00000177103 | DSCAML1 | 0.09768 | 0.0655851 | 0.267632 | 2.74038 | 5.87443 | 3.27626 | 1.97E-14 | 3.414508564 |
| ENSG00000177374 | HIC1 | 1.34165 | 0.616319 | 1.36943 | 2.70426 | 1.99209 | 4.77957 | 0.01859753 | 1.50874862 |
| ENSG00000177469 | PTRF | 37.6148 | 31. 1852 | 32.4399 | 66.7967 | 64.467 | 69.0083 | 2.68E-07 | 1.000957147 |
| ENSG00000177494 | ZBED2 | 2.22956 | 1. 10454 | 0.838431 | 40.9847 | 29.6654 | 58.5521 | 1.93E-10 | 4.937989173 |
| ENSG00000178038 | ALS2CL | 7.83974 | 10.0233 | 11.0492 | 40. 1284 | 30.4679 | 72. 1685 | 0.00059942 | 2.62861587 |
| ENSG00000178177 | LCORL | 3.65066 | 4.73144 | 4.38785 | 8.54408 | 9.70106 | 7.65697 | 1.05E-06 | 1.029215701 |
| ENSG00000178752 | FAM132B | 10.9716 | 9.22193 | 9.6426 | 22.4429 | 14.6378 | 24.048 | 5.71E-05 | 1. 108344605 |
| ENSG00000178999 | AURKB | 79.7981 | 45.9398 | 45.3713 | 130.265 | 116.895 | 148.856 | 1.73E-06 | 1.214716892 |
| ENSG00000179168 | GGN | 0. 125362 | 0.26025 | 0. 118904 | 0.897745 | 0.881326 | 2.05828 | 8.56E-06 | 2.526072598 |
| ENSG00000179348 | GATA2 | 22.6319 | 22.709 | 23.2718 | 48.7561 | 46.3168 | 52.6556 | 5.52E-10 | 1. 146789869 |
| ENSG00000179532 | DNHD1 | 2.06321 | 2.21091 | 1.60268 | 3.84542 | 4.0103 | 6.51412 | 0.0030395 | 1.366370618 |
| ENSG00000179546 | HTR1D | 1.21591 | 1.55544 | 1. 19396 | 6.67374 | 6.01682 | 4.65031 | 5.67E-11 | 2. 140747536 |
| ENSG00000179715 | PCED1B | 0. 172645 | 0.239894 | 0.218614 | 1.78102 | 1.01336 | 1.72449 | 7.76E-08 | 2.93093227 |
| ENSG00000179750 | APOBEC3B | 39.7964 | 32.2711 | 35.3763 | 69.9936 | 72. 1305 | 89.0014 | 5.70E-09 | 1. 110337638 |
| ENSG00000180667 | YOD1 | 3.685 | 4.08361 | 3.41721 | 9.39773 | 8.52367 | 10.6635 | 2.04E-05 | 1.372625641 |
| ENSG00000180739 | S1PR5 | 1.24527 | 1.41374 | 1.67896 | 3.28936 | 2.72503 | 2.32702 | 0.01340172 | 1.030944935 |
| ENSG00000180914 | OXTR | 1.46419 | 0.237849 | 0. 14014 | 0.849477 | 0.777624 | 1.98797 | 3.06E-06 | 2.260401033 |
| ENSG00000181381 | DDX60L | 1.33652 | 1.81231 | 1.54661 | 4.66071 | 3.2564 | 6.36691 | 6.95E-07 | 1.526819118 |
| ENSG00000181652 | ATG9B | 0.389503 | 0.511191 | 0.375065 | 2.4311 | 0.953393 | 3.92872 | 0.01700558 | 2.561683935 |
| ENSG00000181773 | GPR3 | 4.77876 | 4.0986 | 3.60753 | 9.09466 | 6.45092 | 11.2476 | 0.005124 | 1. 131308604 |
| ENSG00000182179 | UBA7 | 0.559204 | 0.489776 | 0.528474 | 7.23042 | 6.74203 | 7.71329 | 4.45E-39 | 3.801157775 |
| ENSG00000182326 | C1S | 0.636013 | 0.271341 | 0.214933 | 1.81428 | 1.85691 | 3.07836 | 0.00098922 | 2.543833511 |
| ENSG00000182481 | KPNA2 | 91.9034 | 59.0484 | 49.4833 | 134.721 | 140.45 | 165.94 | 0.00010548 | 1. 103631541 |
| ENSG00000182685 | BRICD5 | 3.91354 | 3. 19773 | 4.0801 | 11.3696 | 11.6021 | 15.7143 | 3.06E-11 | 1.897567045 |
| ENSG00000182732 | RGS6 | 0.059303 | 0.244156 | 0. 181547 | 0.90951 | 0.959261 | 0.382926 | 1. 19E-05 | 2.850857313 |
| ENSG00000182742 | HOXB4 | 4.90209 | 3.82574 | 3.51147 | 9.48376 | 8.25012 | 16.355 | 0.00855169 | 1.506023173 |
| ENSG00000182795 | C1orf116 | 1.83324 | 2.00279 | 1.96698 | 4.69157 | 4.62066 | 5.43653 | 1.23E-09 | 1.53935715 |
| ENSG00000182798 | MAGEB17 | 0.566883 | 0.345466 | 0.245373 | 11.7532 | 10.4481 | 23. 1453 | 3.90E-08 | 5.422647304 |
| ENSG00000182866 | LCK | 1.79195 | 1.86099 | 2.0807 | 4.21943 | 3. 11967 | 3.98708 | 0.00410594 | 1. 12428587 |
| ENSG00000182870 | GALNT9 | 0.423612 | 0.552064 | 0.677783 | 8.59069 | 4.65474 | 14.3246 | 4.76E-06 | 4. 111983499 |
| ENSG00000182963 | GJC1 | 0.439506 | 0. 134174 | 0.539655 | 0.968896 | 0.790732 | 1.86596 | 6.40E-05 | 3.334785792 |
| ENSG00000183638 | RP1L1 | 0.02841 | 0.0793316 | 0.0765866 | 0. 119604 | 0. 106992 | 0. 198941 | 0.03315438 | 1.202677251 |
| ENSG00000183773 | AIFM3 | 1.8479 | 1. 16857 | 1.54255 | 3.70182 | 3.22277 | 4.67071 | 6.65E-06 | 1.670890996 |
| ENSG00000183856 | IQGAP3 | 11.0438 | 8.06788 | 6.43077 | 18.7752 | 16.4613 | 26.4754 | 0.00043302 | 1.258840464 |
| ENSG00000183891 | TTC32 | 2.99149 | 3.03893 | 3.36616 | 5.26137 | 7.62136 | 8.02798 | 0.01184266 | 1. 179776907 |
| ENSG00000183963 | SMTN | 9. 14043 | 4.80315 | 4.80519 | 15.9978 | 15.0917 | 24.2586 | 8.24E-08 | 1.580539537 |
| ENSG00000184163 | FAM132A | 1.97648 | 0.689644 | 1. 1273 | 2.89281 | 2.25458 | 3.26742 | 0.01881872 | 1.218557301 |
| ENSG00000184254 | ALDH1A3 | 11.284 | 11.0906 | 9.57325 | 96.2047 | 72.9118 | 163.8 | 5.52E-06 | 3.39895786 |
| ENSG00000184292 | TACSTD2 | 36.5928 | 20.4788 | 18.4802 | 102. 126 | 67. 1937 | 191.901 | 0.00538651 | 2.273730102 |
| ENSG00000184313 | MROH7 | 0. 147238 | 0.24322 | 0.252192 | 1.29737 | 0.701329 | 0.865005 | 3.43E-05 | 2.368148443 |

| ENSG00000184371 | CSF1 | 7.47662 | 7.66335 | 6.99991 | 14.6047 | 15.2902 | 14. 1257 | 1.41E-08 | 1. 103574871 |
| --- | --- | --- | --- | --- | --- | --- | --- | --- | --- |
| ENSG00000184445 | KNTC1 | 12.9675 | 10.0431 | 11. 156 | 23.9247 | 26.6696 | 32. 1481 | 8.37E-10 | 1. 148748459 |
| ENSG00000184557 | SOCS3 | 3.31606 | 1.74207 | 2.85374 | 5.74103 | 4.08396 | 8.3889 | 0.0065888 | 1.447477488 |
| ENSG00000184574 | LPAR5 | 1.71588 | 0.933403 | 1.04086 | 3.75617 | 4.27699 | 4.46412 | 5.72E-07 | 1.833794559 |
| ENSG00000184661 | CDCA2 | 6.50642 | 5.96915 | 5.08946 | 12. 1802 | 14.8122 | 11.3996 | 3.64E-08 | 1.099275225 |
| ENSG00000184900 | SUMO3 | 36.7405 | 31.3368 | 33.8704 | 77.7361 | 74.4698 | 71. 1394 | 8.75E-10 | 1. 142185973 |
| ENSG00000184992 | BRI3BP | 11. 1 188 | 6. 13206 | 5.52143 | 16.7678 | 20. 1203 | 12.9983 | 0.00070704 | 1. 121833179 |
| ENSG00000185614 | FAM212A | 0.694356 | 1.5925 | 1.54415 | 2.53791 | 2.45021 | 3.8846 | 0.01546467 | 1.224758715 |
| ENSG00000185697 | MYBL1 | 1.40968 | 1.04063 | 1. 15212 | 5.5802 | 5.50881 | 4.62477 | 1.37E-12 | 1.962625025 |
| ENSG00000185753 | CXorf38 | 8. 17749 | 8.51604 | 7.78495 | 16.5078 | 15.8335 | 17. 1223 | 2.37E-08 | 1.093795784 |
| ENSG00000185842 | DNAH14 | 4.87899 | 7.9657 | 5.34452 | 15.7113 | 13.4094 | 19. 1843 | 8.42E-06 | 1.26101384 |
| ENSG00000185955 | C7orf61 | 0.432619 | 0.526634 | 1.21449 | 1.89512 | 2. 12207 | 4.81595 | 0.03991554 | 2. 10015379 |
| ENSG00000186185 | KIF18B | 18.0216 | 11.4793 | 11.3949 | 30.0068 | 32. 178 | 33.4508 | 1. 14E-11 | 1.248539869 |
| ENSG00000186480 | INSIG1 | 54.8317 | 28.0217 | 31.7584 | 137.815 | 150.648 | 62.4063 | 0.00611268 | 1.633140512 |
| ENSG00000186529 | CYP4F3 | 0. 116728 | 0.209544 | 0.276742 | 0.528187 | 0.683664 | 0.848318 | 0.00012179 | 2.010107313 |
| ENSG00000186577 | C6orf1 | 29. 1211 | 21.7173 | 29.4099 | 58.2272 | 42.0506 | 62.5237 | 1.22E-06 | 1.057927985 |
| ENSG00000186767 | SPIN4 | 7. 13361 | 6.25349 | 4.54889 | 12.4238 | 12.9546 | 10.5717 | 3.04E-06 | 1.008977361 |
| ENSG00000186871 | ERCC6L | 3. 1899 | 2.42075 | 1.72071 | 7.4754 | 7.60867 | 6.62517 | 3.55E-11 | 1.522910231 |
| ENSG00000187098 | MITF | 1.3646 | 2.284 | 1.3694 | 4.65267 | 3.35709 | 2.90141 | 0.00077799 | 1.056148458 |
| ENSG00000187244 | BCAM | 22.8267 | 25.6804 | 36.5221 | 85.6661 | 65.4147 | 109.419 | 9. 18E-07 | 1.708957884 |
| ENSG00000187583 | PLEKHN1 | 3.74395 | 6. 14716 | 5.4789 | 9.95551 | 7.52088 | 15.291 | 0.01490388 | 1.046717444 |
| ENSG00000187609 | EXD3 | 13.6547 | 13.9917 | 17.5205 | 42.6245 | 25.7713 | 46.9553 | 0.00271175 | 1. 167877161 |
| ENSG00000187688 | TRPV2 | 0.598745 | 0.36395 | 0.202778 | 1.31182 | 0.887376 | 0.602965 | 0.01053864 | 2.068901906 |
| ENSG00000187720 | THSD4 | 5.63611 | 1. 17021 | 0.555668 | 4.44572 | 2.97178 | 2.55422 | 2.51E-09 | 2.046609035 |
| ENSG00000188015 | S100A3 | 6.44278 | 2.53091 | 3.74572 | 15. 1119 | 12.8894 | 20.3603 | 0.00014807 | 1.840425373 |
| ENSG00000188039 | NWD1 | 0.080905 | 0.0924973 | 0.0808924 | 0.374806 | 0.278679 | 0.325124 | 9.94E-05 | 2.0314415 |
| ENSG00000188064 | WNT7B | 1.24009 | 3.37026 | 2.86666 | 6.6651 | 4.85732 | 7. 15174 | 2.69E-05 | 1.485874784 |
| ENSG00000188095 | MESP2 | 0.69148 | 0.751054 | 0.637732 | 1.07475 | 0.850843 | 2.01356 | 0.01207131 | 1.408204727 |
| ENSG00000188100 | FAM25A | 0.595447 | 0 | 0 | 55.2291 | 18.2098 | 205.95 | 0.00911173 | 9.018285835 |
| ENSG00000188177 | ZC3H6 | 1.0529 | 1.43721 | 1.37812 | 2.79006 | 3. 17271 | 2.46016 | 2.35E-06 | 1. 110983726 |
| ENSG00000188229 | TUBB4B | 406.853 | 266.373 | 244.601 | 625.849 | 638. 104 | 864.235 | 1.77E-06 | 1.221065703 |
| ENSG00000188549 | C15orf52 | 1.7101 | 2.86388 | 1.98609 | 6.79529 | 5.74227 | 9.282 | 0.00307352 | 1.719942438 |
| ENSG00000188610 | FAM72B | 0.34513 | 0.455639 | 0.289278 | 0.802229 | 0.993617 | 0.849255 | 0.02190252 | 1.309783273 |
| ENSG00000188613 | NANOS1 | 4. 19941 | 4.71807 | 6.68665 | 11.5889 | 7. 184 | 16.634 | 0.03892329 | 1.466228337 |
| ENSG00000188766 | SPRED3 | 0.52012 | 0.674307 | 0.462411 | 1.32536 | 1.25883 | 1.73594 | 0.00035103 | 1.410364109 |
| ENSG00000188897 | CTD-3088G3 .8 | 0.459142 | 0.284268 | 0.300506 | 0.972997 | 0.883257 | 2. 15082 | 0.00114789 | 1.822444594 |
| ENSG00000188910 | GJB3 | 22.2825 | 11.9654 | 13. 1395 | 98.6719 | 68. 1853 | 138.273 | 1.97E-07 | 2.699499169 |
| ENSG00000189280 | GJB5 | 0.674646 | 0.532306 | 0.483278 | 5.65851 | 5.56294 | 4.67776 | 1.63E-12 | 3.302423018 |
| ENSG00000189410 | SH2D5 | 0. 126066 | 0.0791357 | 0.078387 | 2.79798 | 0.98268 | 2.47794 | 8.39E-08 | 4.039813234 |
| ENSG00000189433 | GJB4 | 0. 179771 | 0.082606 | 0. 1 1855 | 1.57593 | 1.05603 | 1.36517 | 7.63E-11 | 3.43819776 |
| ENSG00000196296 | ATP2A1 | 4.8425 | 4.649 | 4. 13776 | 12.7544 | 9.25414 | 20. 1851 | 0.00289889 | 1.561301219 |
| ENSG00000196337 | CGB7 | 1.92207 | 1. 12823 | 0.967866 | 5. 16796 | 2.9316 | 8.99364 | 0.00139936 | 2.222927313 |
| ENSG00000196535 | MYO18A | 21.5971 | 22.274 | 20.8689 | 53.9006 | 46.666 | 71.2938 | 6.52E-10 | 1.390138161 |

| ENSG00000196550 | FAM72A | 2. 16619 | 1.41322 | 1.54435 | 5.0726 | 4.59062 | 4.60658 | 2.22E-05 | 1.716768865 |
| --- | --- | --- | --- | --- | --- | --- | --- | --- | --- |
| ENSG00000196639 | HRH1 | 1.84311 | 2.07071 | 1.42623 | 3.91756 | 3.42326 | 3.62606 | 0.00053894 | 1.063382336 |
| ENSG00000196754 | S100A2 | 21.8576 | 5.87879 | 9.5303 | 44.778 | 31.5832 | 38.7588 | 7.29E-07 | 1.506585845 |
| ENSG00000197142 | ACSL5 | 36.8013 | 32.8264 | 24.6941 | 68.0107 | 65.7357 | 57.7567 | 1. 17E-07 | 1.002297073 |
| ENSG00000197191 | CYSRT1 | 8.39134 | 6.45239 | 6.59362 | 15.6412 | 11.3428 | 21.8457 | 0.0038066 | 1.21574063 |
| ENSG00000197381 | ADARB1 | 4.97238 | 4.28359 | 3.38289 | 11. 1912 | 11.5515 | 12. 1252 | 5.72E-14 | 1.448782094 |
| ENSG00000197467 | COL13A1 | 1.47965 | 1.42754 | 1.01466 | 12.5164 | 8.93113 | 25.0351 | 1.47E-05 | 3.487610899 |
| ENSG00000197576 | HOXA4 | 1.20397 | 0.815272 | 1.98392 | 3.07091 | 2. 17827 | 3.46292 | 0.00794727 | 1.282082678 |
| ENSG00000197912 | SPG7 | 43. 157 | 30.01 | 27.2301 | 73.2038 | 61. 1 155 | 87.4153 | 7.55E-07 | 1. 100190452 |
| ENSG00000198108 | CHSY3 | 0.016505 | 0.238057 | 0.20427 | 1.77607 | 1.61642 | 1.06681 | 3.43E-11 | 3.324138381 |
| ENSG00000198121 | LPAR1 | 5. 1 1343 | 8.73866 | 7.47423 | 19.8532 | 14.0856 | 23.9549 | 0.0023949 | 1.641943305 |
| ENSG00000198133 | TMEM229B | 0.867467 | 0.48803 | 0.60811 1 | 1.82721 | 1.54632 | 2.05159 | 3.41E-08 | 2.26273979 |
| ENSG00000198168 | SVIP | 6. 13637 | 5.59278 | 5.44815 | 14.6849 | 14.2765 | 11.8555 | 1. 13E-09 | 1.237662055 |
| ENSG00000198211 | TUBB3 | 0.024562 | 0.00075987 | 0.0438225 | 1.48121 | 1.07193 | 3.0529 | 1.24E-06 | 6.438879175 |
| ENSG00000198324 | FAM109A | 6.53935 | 5.83054 | 7.90494 | 19.0675 | 12.719 | 22.8078 | 0.00026462 | 1.311083931 |
| ENSG00000198431 | TXNRD1 | 131.932 | 160. 156 | 124.006 | 290.748 | 326.874 | 212.845 | 7.98E-06 | 1.017594979 |
| ENSG00000198554 | WDHD1 | 7.0269 | 4.62503 | 3.69187 | 11.723 | 14.0965 | 7.767 | 0.00289257 | 1.033332302 |
| ENSG00000198648 | STK39 | 8.91711 | 10.0752 | 9.761 | 33.5346 | 26.8869 | 41. 1179 | 6. 17E-07 | 1.832576079 |
| ENSG00000198695 | MT-ND6 | 4901.55 | 879.937 | 1741.28 | 7028.64 | 7551.95 | 4935.67 | 0.01081869 | 1.357481626 |
| ENSG00000198743 | SLC5A3 | 4.38047 | 5.96124 | 5.08501 | 38.9216 | 43.615 | 35.2097 | 8.84E-52 | 2.935701182 |
| ENSG00000198754 | OXCT2 | 1.69643 | 1.34032 | 1.07293 | 2.00864 | 2.89669 | 3.98554 | 0.00938205 | 1. 107522775 |
| ENSG00000198786 | MT-ND5 | 1832.39 | 569.366 | 830.331 | 2729.42 | 3210.37 | 2096.99 | 0.00443713 | 1.281090254 |
| ENSG00000198796 | ALPK2 | 0.025995 | 0.04911 16 | 0.0396822 | 1.96731 | 1.49872 | 3.06568 | 3.60E-11 | 5.869306203 |
| ENSG00000198826 | ARHGAP11A | 20.8424 | 14.4067 | 13.5653 | 44. 1639 | 47.4535 | 36.5821 | 1.63E-12 | 1.315636564 |
| ENSG00000198873 | GRK5 | 1.71887 | 1.27608 | 1.69785 | 3.61227 | 2.70733 | 3.94612 | 0.00110979 | 1. 136449173 |
| ENSG00000198901 | PRC1 | 74.7135 | 45.7569 | 40.3325 | 113. 15 | 99.7361 | 150.426 | 5.99E-09 | 1.098349503 |
| ENSG00000198959 | TGM2 | 1.23237 | 2.23307 | 1.23636 | 13.8492 | 7.64515 | 16.5598 | 1.22E-06 | 3.276122699 |
| ENSG00000203697 | CAPN8 | 0.971976 | 0.520094 | 0.381271 | 2. 17422 | 1.84981 | 5.02913 | 0.00895236 | 1.802815768 |
| ENSG00000204176 | SYT15 | 0.595482 | 0.263386 | 0.379959 | 1.79705 | 2. 19432 | 2.03728 | 4.92E-10 | 2.318544558 |
| ENSG00000204531 | POU5F1 | 0.518061 | 0.276935 | 0.206748 | 0.608381 | 0.952148 | 1. 14406 | 0.00734728 | 1.475381597 |
| ENSG00000204536 | CCHCR1 | 1. 16224 | 3.62351 | 1.59799 | 4. 13156 | 4.35758 | 4.4863 | 0.01963843 | 1. 170090502 |
| ENSG00000204856 | FAM216A | 10. 1523 | 8.22563 | 8. 13724 | 18.574 | 20.9152 | 22. 1006 | 1.03E-07 | 1.208542225 |
| ENSG00000205220 | PSMB10 | 20.3733 | 14.0567 | 15. 1792 | 37.677 | 36.5204 | 36.6686 | 1.83E-08 | 1. 181257186 |
| ENSG00000205307 | SAP25 | 4.67401 | 4.65285 | 5.71634 | 11.7495 | 10.3994 | 20.8518 | 0.00983144 | 1.537540523 |
| ENSG00000205309 | NT5M | 5.99778 | 4.21804 | 5.80233 | 12. 1 133 | 10.8059 | 14.9025 | 3.04E-06 | 1.256694212 |
| ENSG00000205755 | CRLF2 | 0.312941 | 0.361494 | 0.287901 | 1.54132 | 0.745864 | 1.37969 | 0.00030393 | 1.999290924 |
| ENSG00000206075 | SERPINB5 | 1.02917 | 1.09471 | 0.742174 | 9.25561 | 7.38093 | 10.3382 | 5. 13E-17 | 3.221426855 |
| ENSG00000206281 | TAPBP | 0.566761 | 0.68366 | 0.335052 | 1.51733 | 1.3597 | 1. 14437 | 0.00363185 | 1.427743508 |
| ENSG00000206418 | RAB12 | 7.33734 | 7.99198 | 7.65364 | 16. 1499 | 13.418 | 23.2493 | 0.00515453 | 1.204577464 |
| ENSG00000206450 | HLA-B | 10.6203 | 11.7072 | 10. 1786 | 22.5422 | 17. 1964 | 25.813 | 3.39E-06 | 1.016793512 |
| ENSG00000211445 | GPX3 | 7.0539 | 8.92404 | 7.08144 | 19.6695 | 14.8421 | 21.6426 | 4.52E-07 | 1.215604942 |
| ENSG00000212724 | KRTAP2-3 | 0.658292 | 0.332322 | 0.459295 | 17.8253 | 13. 1 145 | 93.3861 | 0.02461065 | 6.535864107 |
| ENSG00000212901 | KRTAP3-1 | 9.76045 | 7.91239 | 3.58171 | 123.384 | 78.0021 | 194.315 | 6.28E-06 | 4.241916224 |

| ENSG00000213199 | ASIC3 | 2.46868 | 2.07841 | 2.43505 | 6.52567 | 4.84647 | 11.4147 | 0.00307709 | 1.757815592 |
| --- | --- | --- | --- | --- | --- | --- | --- | --- | --- |
| ENSG00000213397 | HAUS7 | 13. 1009 | 5.59585 | 7.21053 | 15.5515 | 14.6314 | 20.9574 | 0.00027314 | 1. 154958604 |
| ENSG00000213445 | SIPA1 | 11.7626 | 11. 161 | 10.8292 | 30.2147 | 29.5632 | 33.8886 | 3.30E-15 | 1.459784385 |
| ENSG00000213599 | SLX1A- SULT1A3 | 2.9689 | 3.96785 | 3. 11674 | 5.88963 | 7.78189 | 11.3698 | 0.00226452 | 1.335105585 |
| ENSG00000213689 | TREX1 | 15.642 | 7.59198 | 7.78939 | 27.5844 | 26. 1833 | 31. 1 163 | 1.71E-06 | 1.273349847 |
| ENSG00000213937 | CLDN9 | 4.76082 | 3.49407 | 4.97537 | 10. 1 101 | 7.47516 | 13.5737 | 0.00041735 | 1.267411859 |
| ENSG00000214226 | C17orf67 | 0.41757 | 0.64298 | 0.640483 | 1.85579 | 1.76673 | 2.38138 | 0.00010743 | 1.83455644 |
| ENSG00000214595 | EML6 | 0.478558 | 0.380258 | 0.36106 | 0.885485 | 0.830703 | 1. 13777 | 0.00110001 | 1.215662488 |
| ENSG00000214944 | ARHGEF28 | 2.22416 | 1.99376 | 2.09815 | 6. 17267 | 5.0259 | 8.08298 | 5.03E-12 | 1.559480359 |
| ENSG00000215784 | FAM72D | 0.821293 | 0.875227 | 0.395222 | 1.79973 | 2.42238 | 2.99391 | 0.00018002 | 1.828342684 |
| ENSG00000221947 | XKR9 | 0. 145059 | 0.467043 | 0.676592 | 0.886251 | 1.85537 | 0.904306 | 0.01993461 | 1.435690507 |
| ENSG00000221963 | APOL6 | 1.62537 | 1.89292 | 1.42119 | 4.83048 | 4.91417 | 3.00683 | 5.86E-08 | 1.346403115 |
| ENSG00000223532 | HLA-B | 86.2773 | 114.362 | 112.611 | 219.906 | 177. 109 | 263. 187 | 1.24E-07 | 1.043199441 |
| ENSG00000224501 | HSPA1B | 1.44482 | 0.419545 | 1.7375 | 3.21314 | 2.73 | 2.37897 | 0.00520338 | 1.235253987 |
| ENSG00000224608 | HLA-B | 8. 10474 | 11.7169 | 12. 1946 | 20.3789 | 20.9328 | 26.8255 | 0.00039148 | 1.022161674 |
| ENSG00000227600 | PPT2 | 0.272537 | 0.333523 | 0.293622 | 0.600819 | 0.557496 | 0.732555 | 0.03542021 | 1.207226487 |
| ENSG00000231274 | SBK3 | 0.519572 | 0.362218 | 0.509987 | 6.75896 | 3.5299 | 7.07298 | 1.01E-09 | 3.691879611 |
| ENSG00000232126 | HLA-B | 5. 16982 | 7.32132 | 6.81243 | 16.3771 | 11.8533 | 18.3839 | 1.30E-05 | 1.213527839 |
| ENSG00000232804 | HSPA1B | 1.22277 | 1.20077 | 1.36965 | 3.29518 | 3. 13372 | 2.86367 | 0.00049663 | 1.295269118 |
| ENSG00000233323 | TNXB | 0.008154 | 0.0316317 | 0.0794182 | 0. 150912 | 0. 121281 | 0. 175056 | 0.00023743 | 1.962472089 |
| ENSG00000238243 | OR2W3 | 1.87249 | 1. 19984 | 0.807116 | 3. 10191 | 2.43784 | 4.06559 | 0.01120695 | 1.384641334 |
| ENSG00000239713 | APOBEC3G | 0.310671 | 0.544948 | 0.599274 | 1.09264 | 0.858254 | 1.78119 | 0.04402719 | 1.516423873 |
| ENSG00000240891 | PLCXD2 | 0.257938 | 0.957235 | 0.739397 | 2.63542 | 2.92953 | 2. 16994 | 1.87E-06 | 2. 143411456 |
| ENSG00000241360 | PDXP | 19. 1682 | 14.2997 | 15.7343 | 36.2685 | 29.829 | 43.7539 | 3.20E-09 | 1. 165766847 |
| ENSG00000241399 | CD302 | 0. 150106 | 0.50328 | 0.244143 | 0.983069 | 0.887138 | 0.887764 | 0.00093184 | 1.688607731 |
| ENSG00000241978 | AKAP2 | 2.98825 | 3. 1 1628 | 2.23403 | 9.72655 | 8.85439 | 10.8625 | 2.92E-18 | 1.825722343 |
| ENSG00000242866 | STRC | 0.66075 | 0.88705 | 0.844797 | 1.57359 | 1.36955 | 2.35758 | 0.00735545 | 1. 149214962 |
| ENSG00000243477 | NAT6 | 16.9348 | 13. 154 | 16.7383 | 36.8061 | 29.498 | 42.9272 | 1.27E-09 | 1.305299451 |
| ENSG00000243811 | APOBEC3D | 0.629396 | 0. 199188 | 0.0900962 | 0.740793 | 1.06586 | 0.854905 | 0.00010166 | 2. 128386852 |
| ENSG00000243927 | MRPS6 | 70.6909 | 56.6126 | 64.941 | 214.57 | 215.346 | 291.364 | 2.47E-10 | 1.909215794 |
| ENSG00000249992 | TMEM158 | 1.71076 | 1.40153 | 1.90146 | 4.87899 | 3.2795 | 10.2165 | 0.02294917 | 1.914045443 |
| ENSG00000250506 | CDK3 | 0.956822 | 0.280857 | 5.60766 | 7.04682 | 7.69523 | 8.06922 | 0.00750641 | 1.826326752 |
| ENSG00000250588 | IQCJ-SCHIP1 | 2.75634 | 2.50514 | 2.20606 | 9.5608 | 4.87317 | 9.45623 | 2.49E-06 | 1.592647962 |
| ENSG00000253276 | CCDC71L | 8.30504 | 7.26314 | 7.78297 | 13.0929 | 12.2988 | 16.2618 | 7. 12E-07 | 1. 11693446 |
| ENSG00000253368 | TRNP1 | 22.7977 | 25.2011 | 23.3325 | 52.4675 | 39.6943 | 62.494 | 9.88E-06 | 1. 194006631 |
| ENSG00000258659 | TRIM34 | 0.454699 | 0.648205 | 0.401643 | 1.45616 | 1.58884 | 1.22144 | 0.00252798 | 1.510115987 |
| ENSG00000260007 | RP11 -  315D16 .2 | 3.80543 | 0.741962 | 1.42432 | 10. 1497 | 8.89136 | 16.4611 | 5.53E-06 | 2.59802548 |
| ENSG00000260238 | PMF1-BGLAP | 2. 1298 | 2.42565 | 2.56735 | 5.55586 | 5.79573 | 3.65785 | 0.01428953 | 1.095660006 |
| ENSG00000263155 | MYZAP | 0.446106 | 0.415128 | 0.695782 | 4.3007 | 4.20891 | 6.62303 | 9.64E-14 | 3.246578352 |
| ENSG00000264230 | ANXA8L1 | 0. 111994 | 0.0318757 | 0. 157716 | 3.73182 | 3.71267 | 4.36502 | 3.41E-21 | 5.20934599 |
| ENSG00000265190 | ANXA8 | 0.739206 | 0.518448 | 0.371749 | 11.9972 | 12.9379 | 19.9201 | 1.21E-15 | 4.801546044 |
| ENSG00000265590 | AP000275 .65 | 0.422576 | 0.63179 | 0.34973 | 2.96384 | 2.30596 | 2.99352 | 3.08E-06 | 2.571642343 |
| ENSG00000265763 | ZNF488 | 0.64052 | 0.534974 | 0.391158 | 9.53817 | 8.56291 | 9.21093 | 2.38E-51 | 4. 138031015 |

| ENSG00000265972 | TXNIP | 6.88943 | 6.30191 | 5.00788 | 33.0687 | 24.0078 | 13.2129 | 0.00294276 | 1.924396815 |
| --- | --- | --- | --- | --- | --- | --- | --- | --- | --- |
| ENSG00000266094 | RASSF5 | 4.695 | 3.26734 | 3.32303 | 8.44259 | 7.92169 | 7.63962 | 9.52E-06 | 1.073477555 |
| ENSG00000271447 | MMP28 | 0.569198 | 0.505259 | 0.929715 | 5.26101 | 4.27768 | 5.4609 | 1. 15E-10 | 2.979989265 |
| ENSG00000275993 | CH507- 42P11 .8 | 1.69971 | 0.823889 | 0.905872 | 2.58748 | 1.89836 | 3.36071 | 0.00578297 | 1.233487263 |
| ENSG00000277758 | SYT15 | 0. 177829 | 0. 146339 | 0.244053 | 1.73844 | 1.55693 | 1.72608 | 1.52E-14 | 3.221601756 |
| ENSG00000278023 | RDM1 | 2.62107 | 2.04107 | 1. 17151 | 5.87605 | 5.05652 | 4.88469 | 0.00510728 | 1.294219289 |
| ENSG00000278540 | ACACA | 20.9767 | 18. 1645 | 21.4087 | 40.7081 | 44. 1179 | 34.2086 | 0.00061686 | 1.068529852 |
| ENSG00000279468 | AC114494 . 1 | 0.249283 | 0. 132248 | 0. 147178 | 1.35237 | 1.2377 | 2.44317 | 4.90E-05 | 3.334490318 |
| ENSG00000280804 | TREX1 | 1.96387 | 1. 15665 | 0.899684 | 2.77005 | 3.05562 | 3.49026 | 0.00225817 | 1.286190573 |

| **Supplementary Table 2. Clinical Characteristics of 10 CRC Patients Involved in This Study** | | | | | |
| --- | --- | --- | --- | --- | --- |
| Characteristics | P01 | P02 | P03 | P04 | P05 |
| Age | 55 | 86 | 48 | 69 | 79 |
| Gender | Female | Male | Female | Male | Female |
| Histological typea | Colon | Colon | Rectum | Rectum | Colon |
|  | ADC | ADC | ADC | ADC | ADC |
| Locationb | LCC | LCC | ReC | ReC | RCC |
| pTNM: T | 3 | 3 | 3 | 3 | 3 |
| pTNM: N | 0 | 0 | 0 | 0 | 1 |
| pTNM: M | 0 | 0 | 0 | 0 | 0 |
| Stage | ⅡA | ⅡA | ⅡA | ⅡA | ⅢB |
| Tumour size | 3.5×3×1.5 cm | 2.2×1.8×0.8 cm | 2.2×1.8×0.7 cm | 4×2.5×1.5 cm | 4.5×3×1 cm |
|  |  |  |  |  |  |
|  |  |  |  |  |  |
| Characteristics | P06 | P07 | P08 | P09 | P10 |
| Age | 56 | 64 | 46 | 55 | 74 |
| Gender | Male | Male | Male | Male | Male |
| Histological typea | Colon | Colon | Colon | Rectum | Rectum |
|  | ADC | ADC | ADC | ADC | ADC |
| Locationb | RCC | RCC | LCC | ReC | ReC |
| pTNM: T | 3 | 3 | 3 | 3 | 3 |
| pTNM: N | 0 | 0 | 0 | 0 | 0 |
| pTNM: M | 0 | 0 | 0 | 0 | 0 |
| Stage | ⅡA | ⅡA | ⅡA | ⅡA | ⅡA |
| Tumour size | 5.5×4×0.8 cm | 3×2×1 cm | 3×2×1.5 cm | 6×3×3 cm | 5×4×1 cm |
|  |  |  |  |  |  |
| a ADC, adenocarcinoma |  |  |  |  |  |
| b RCC, right-sided colon cancer; ReC, rectum cancer. |  |  |  |  |  |

**Supplementary Table 3. Primers for RT quantitative PCR**

| Gene ID | Primers | Sequences（5 ’→3’） |
| --- | --- | --- |
| 8694 | *DGAT1*-RT forward | TATTGCGGCCAATGTCTTTGC |
|  | DGAT1-RT reverse | CACTGGAGTGATAGACTCAACCA |
| 2597 | *GAPDH*-RT forward | GGAGCGAGATCCCTCCAAAAT |
|  | *GAPDH*-RT reverse | GGCTGTTGTCATACTTCTCATGG |
| 3586 | *DGAT2*-RT forward | ATTGCTGGCTCATCGCTGT |
|  | *DGAT2*-RT reverse | GGGAAAGTAGTCTCGAAAGTAGC |
