# Supplementary material for: Autophagic flux-lipid droplet biogenesis cascade sustains mitochondrial fitness in colorectal cancer cells adapted to acidosis
Source: Cell Death Discov. 2025 Jan 25;11:21. doi: 10.1038/s41420-025-02301-6 (PMC11761495; doi:10.1038/s41420-025-02301-6)

Full and Uncropped Western Blots

Figure 1


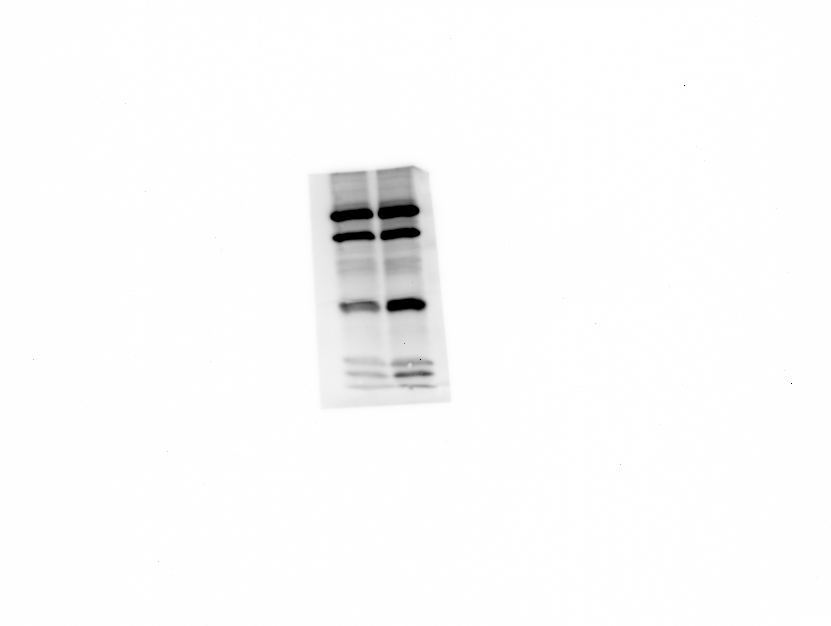

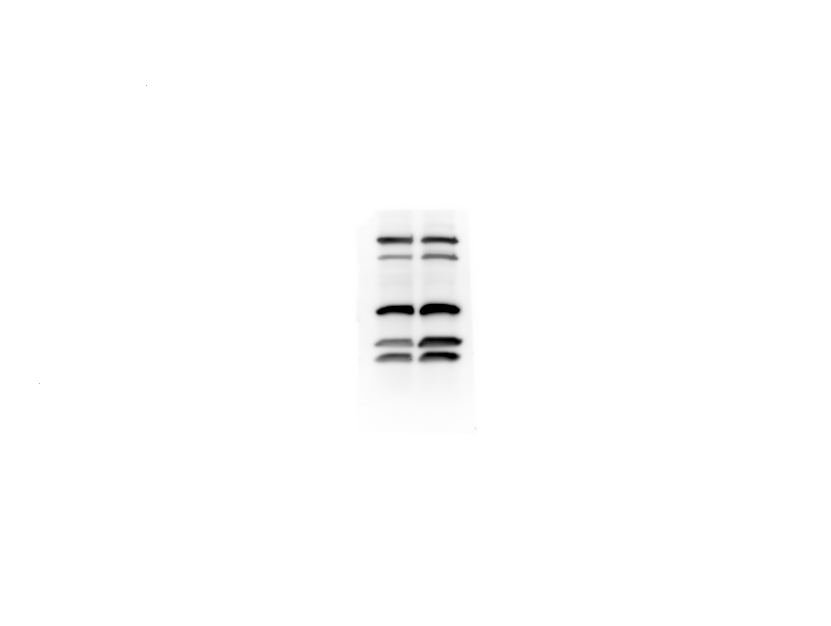

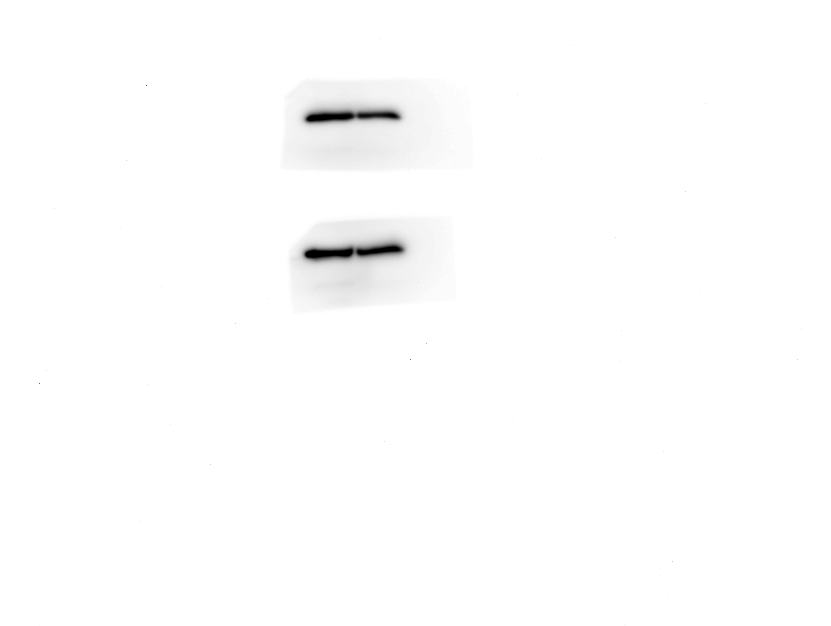


Figure 3


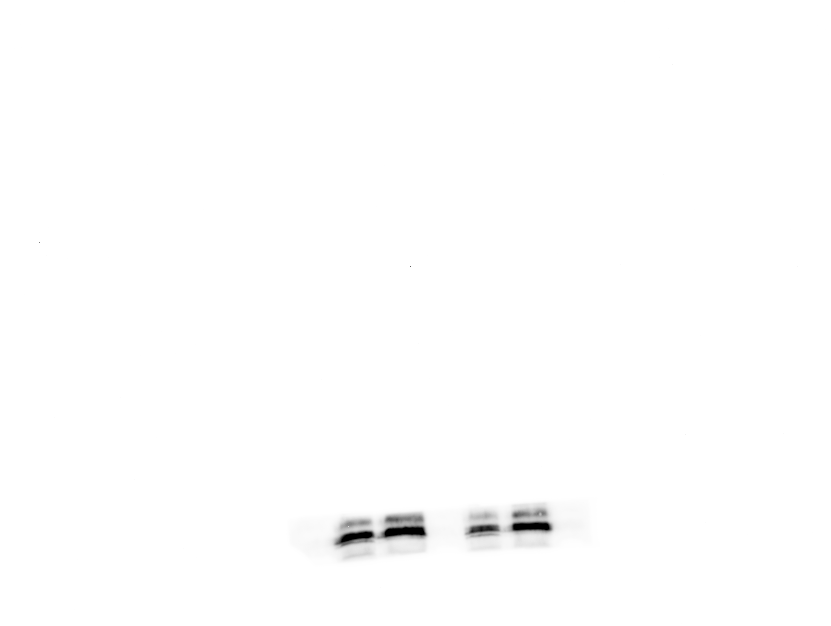


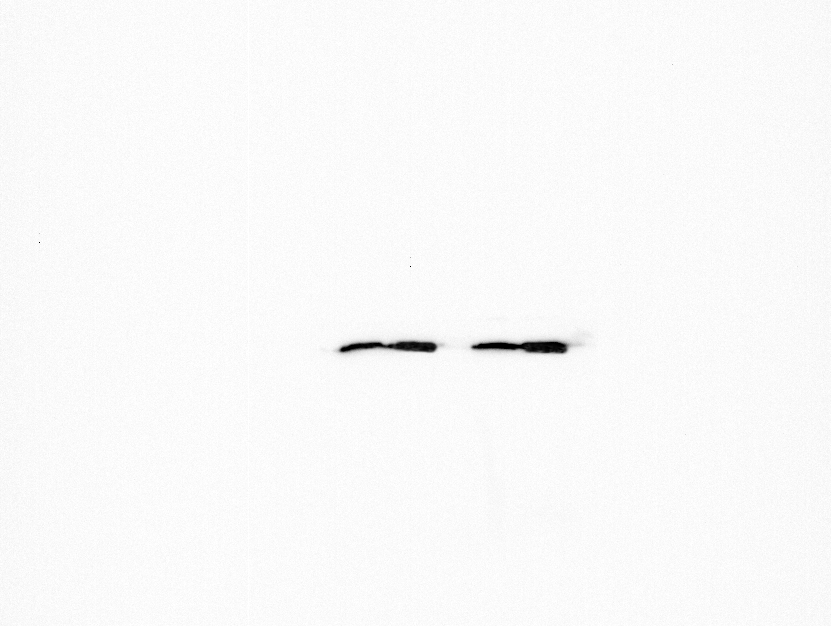


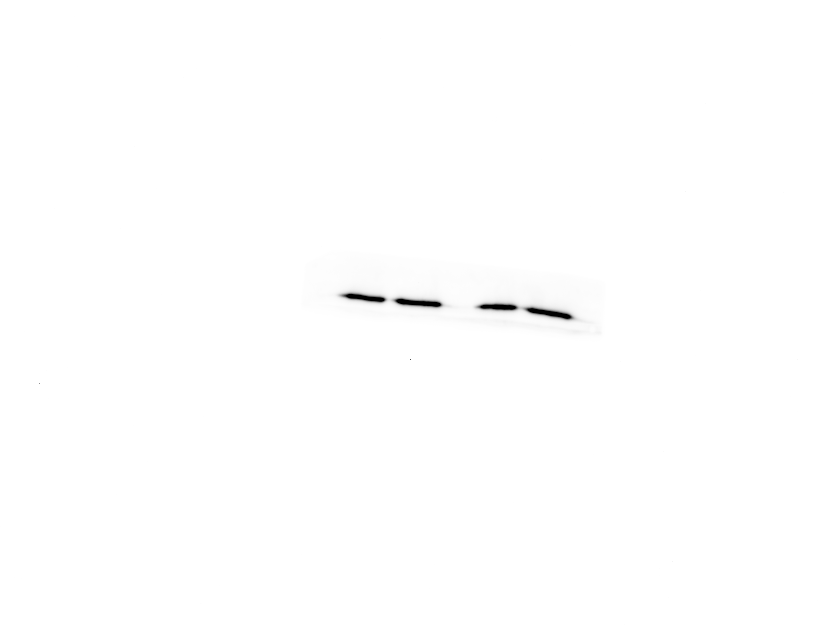

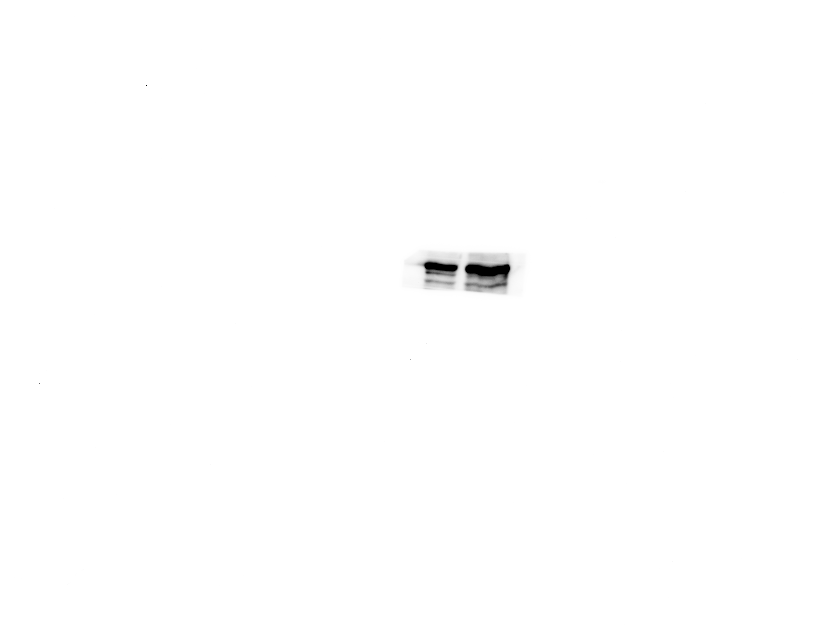

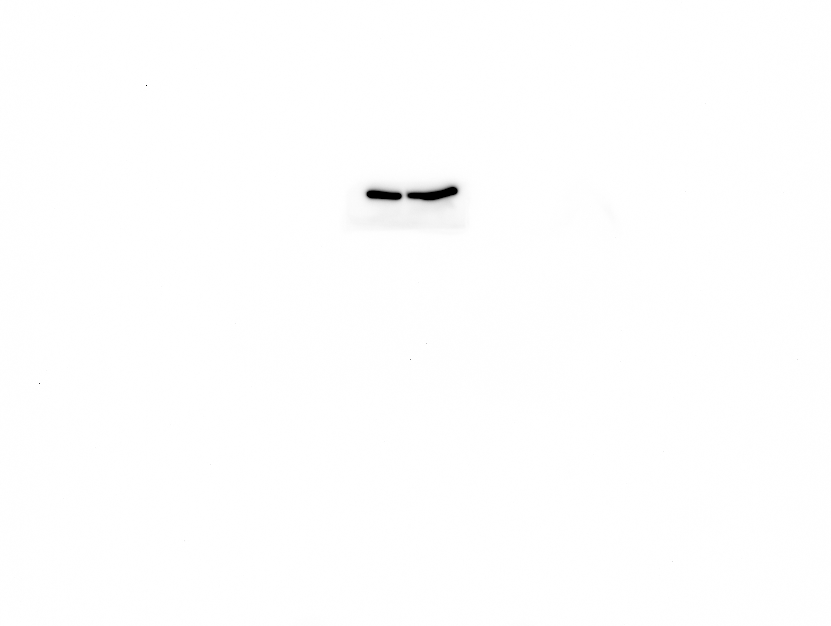

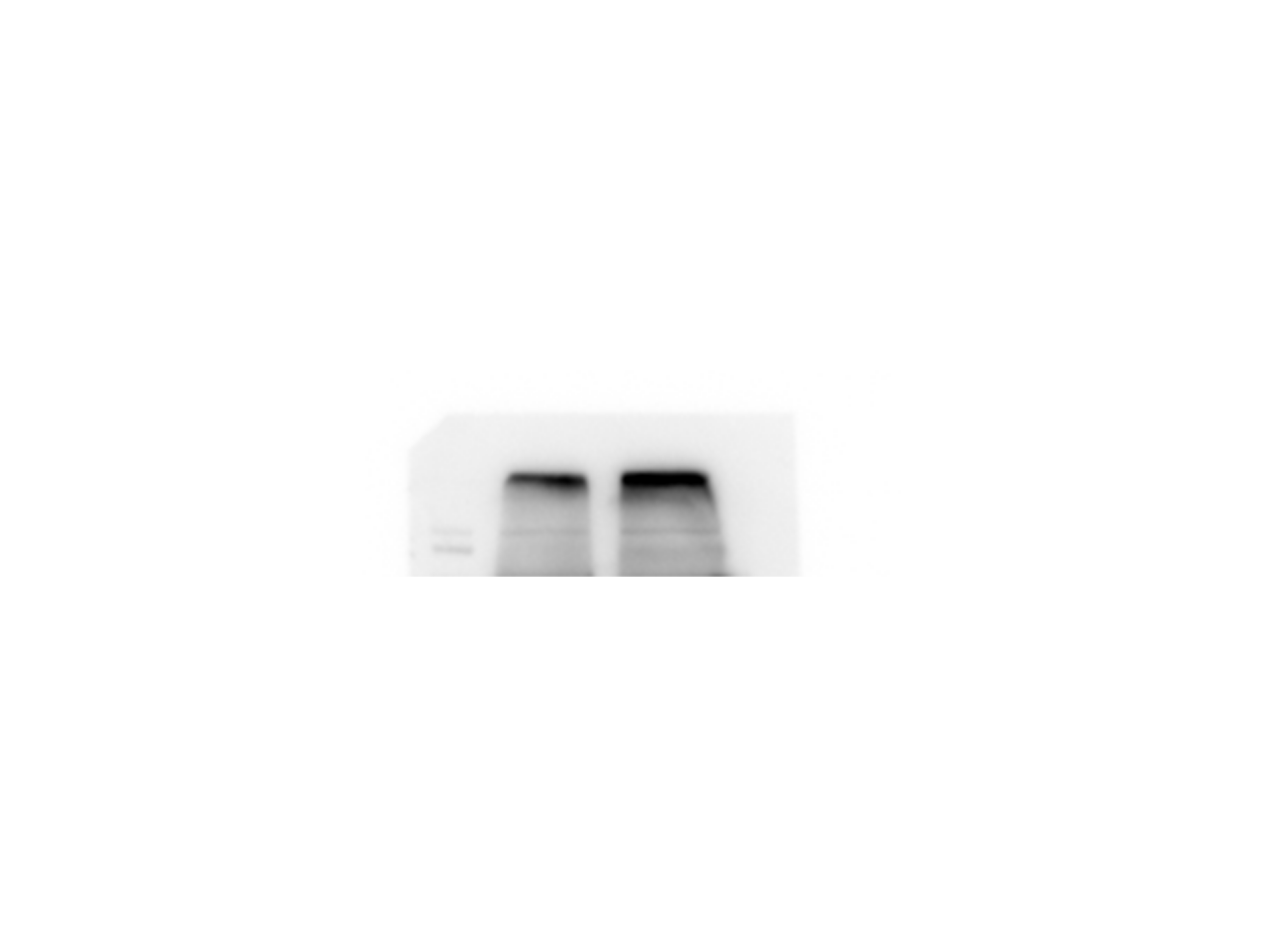

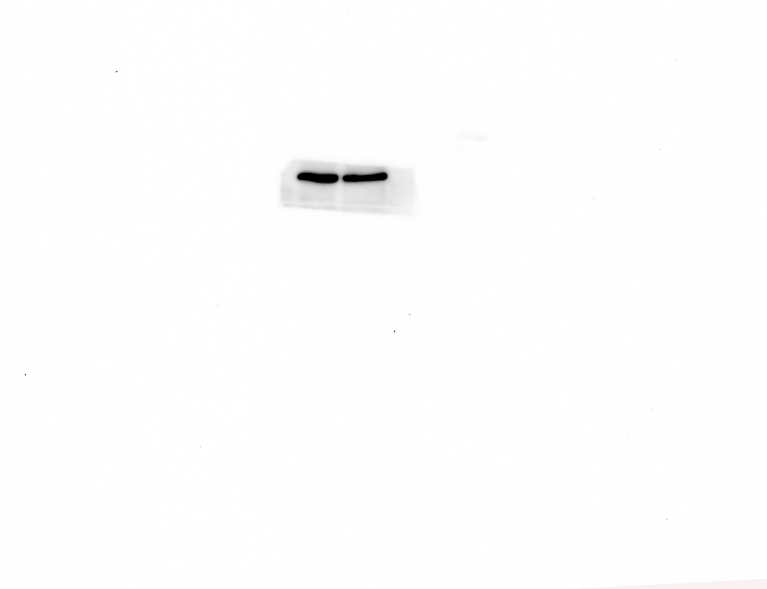


Figure 4


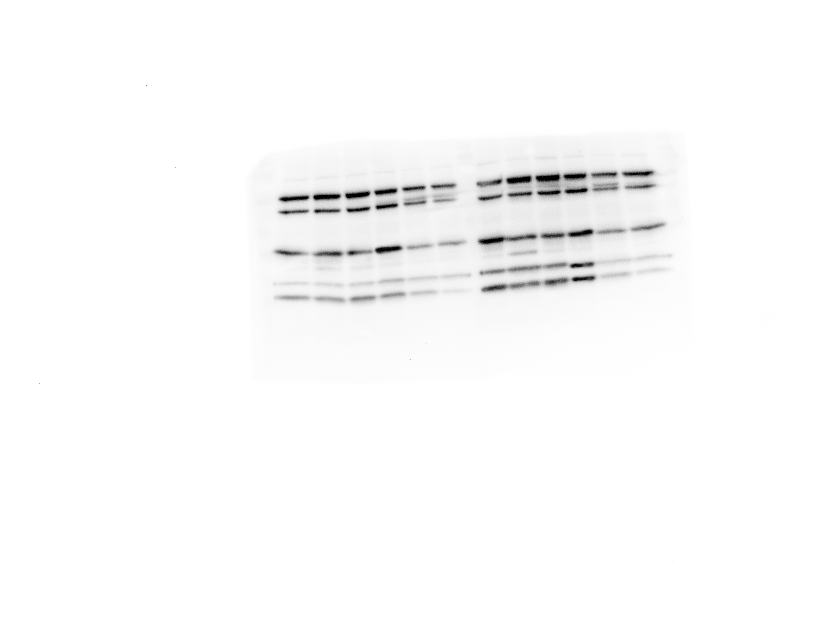


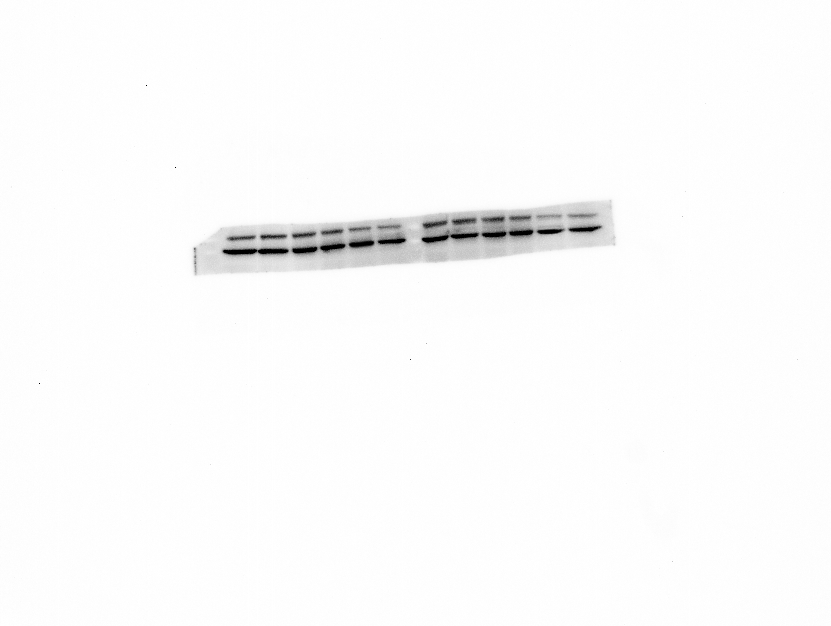


Supplement Figure 2D


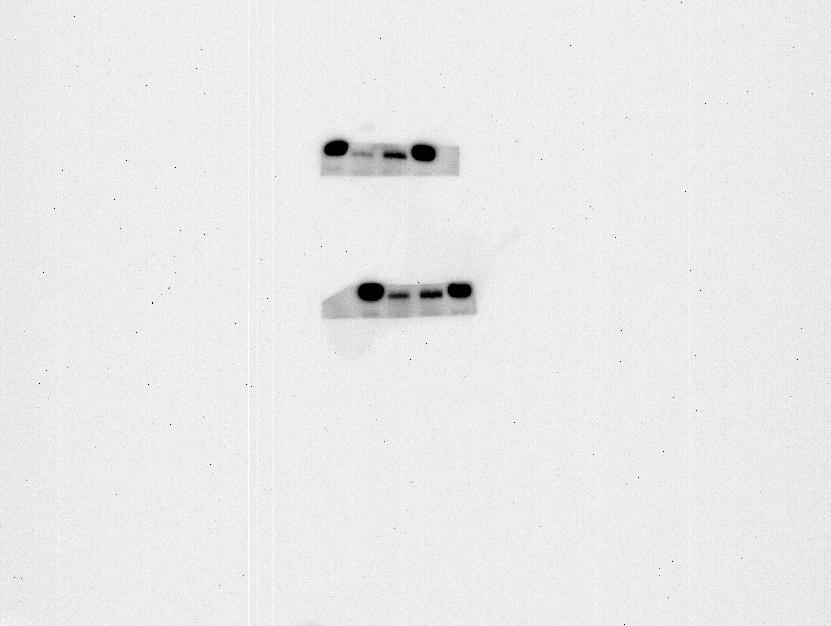


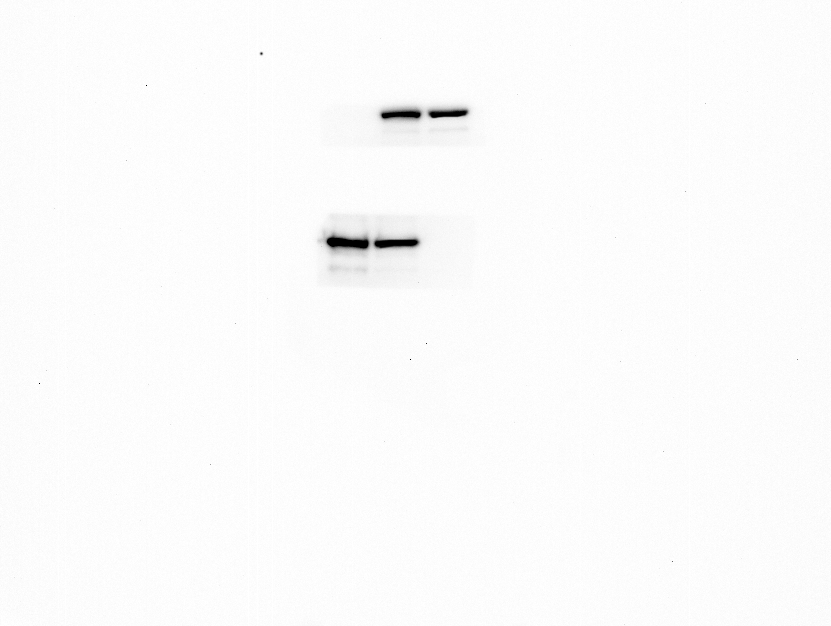

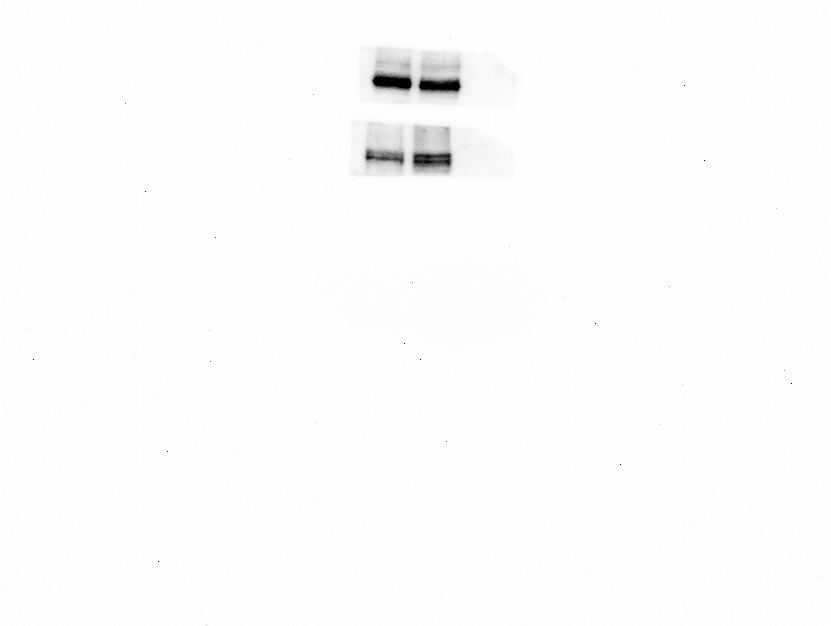

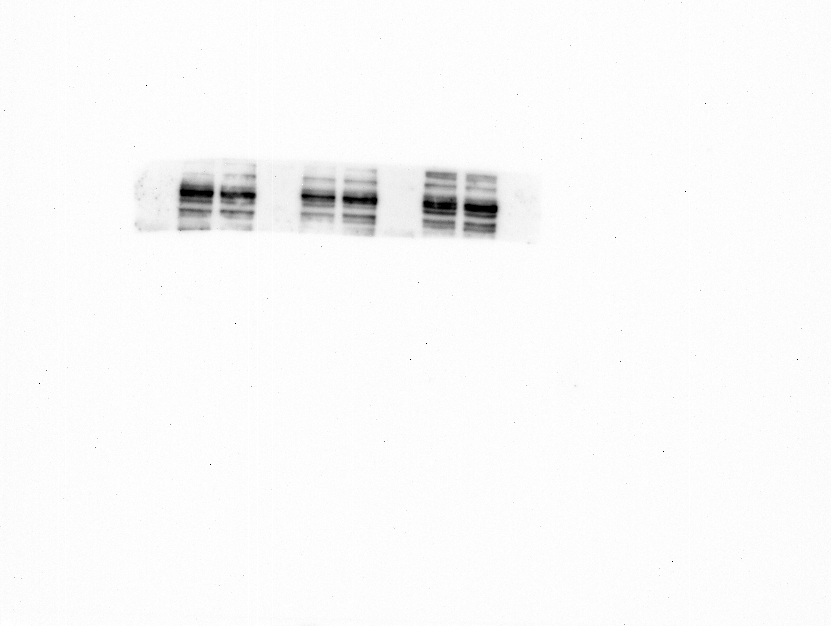

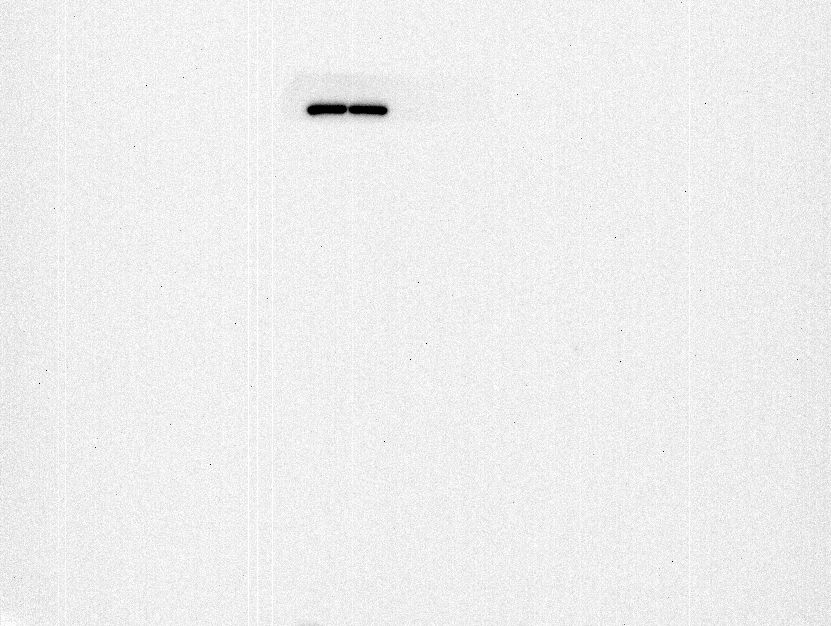


Supplement Figure 2C, 2E


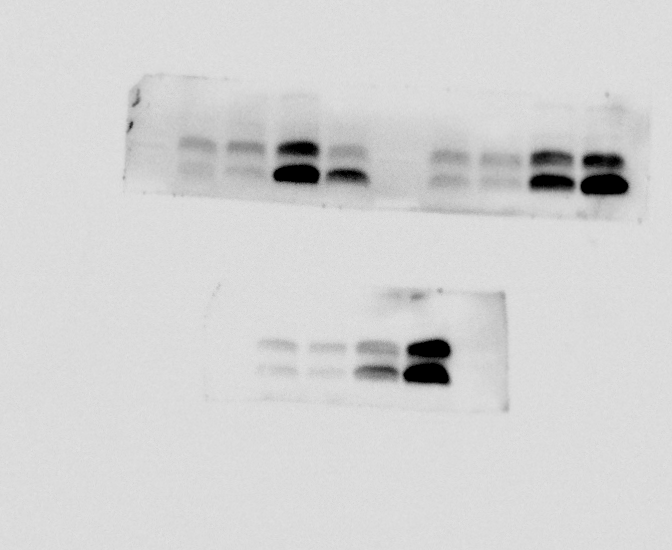


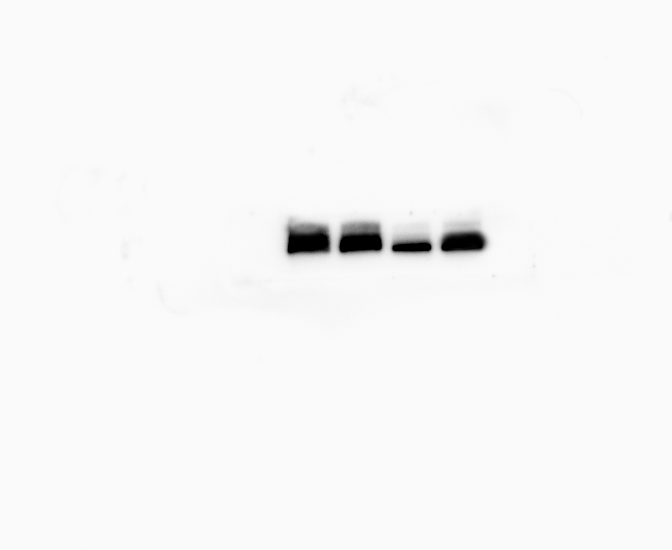

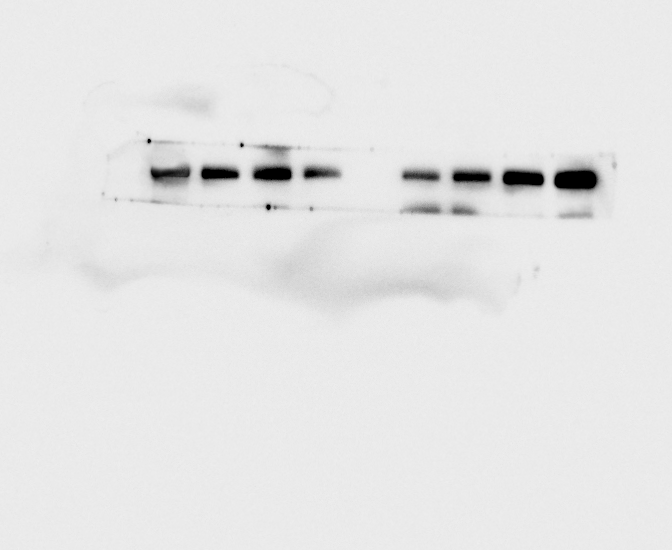

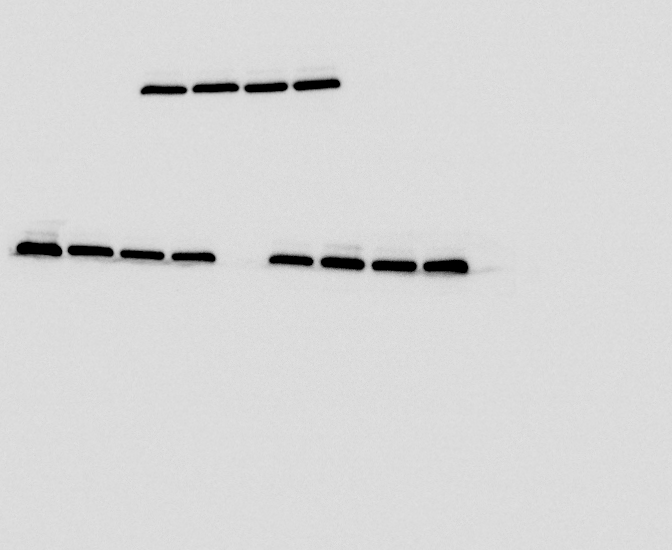

Supplement: Supplementary file 4 — Original Western Blot [file 41420_2025_2301_MOESM4_ESM.docx]
